# BACH1-induced ferroptosis drives lymphatic metastasis by repressing the biosynthesis of monounsaturated fatty acids

**DOI:** 10.1038/s41419-023-05571-z

**Published:** 2023-01-20

**Authors:** Xiufeng Xie, Lusong Tian, Yan Zhao, Fang Liu, Shuyang Dai, Xinglu Gu, Yuxin Ye, Lanping Zhou, Xinmiao Liu, Yulin Sun, Xiaohang Zhao

**Affiliations:** State Key Laboratory of Molecular Oncology, National Cancer Center/National Clinical Research Center for Cancer/Cancer Hospital, Chinese Academy of Medical Sciences and Peking Union Medical College, Beijing, 100021 P. R. China

**Keywords:** Metastasis, Cancer metabolism

## Abstract

Esophageal squamous cell carcinoma (ESCC) is one of the fatal malignancies worldwide. It has an increased propensity to metastasize via lymphogenous routes in an early stage. The prognosis of patients with lymph node metastases (LNM) is often worse than that of patients without metastases. Although several factors have been found to influence metastasis, the mechanisms of preference for specific metastatic routes remain poorly understood. Herein, we provide evidence that the intrinsic hypersensitivity of tumor cells to ferroptosis may proactively drive lymphatic metastasis. Serum autoantibodies associated with LNM of early ESCC were screened using a whole-proteome protein array containing 19 394 human recombinant proteins, and an anti-BACH1 autoantibody was first identified. Pan-cancer analysis of ferroptosis-related genes with preferential lymphatic metastasis and preferential hematogenous metastasis based on The Cancer Genome Atlas data was performed. Only BACH1 showed significant overexpression in tumors with preferential lymphatic metastasis, whereas it was downregulated in most tumors with preferential nonlymphatic metastasis. In addition, it was found that the serum levels of autoantibodies against BACH1 were elevated in early-stage patients with LNM. Interestingly, BACH1 overexpression and ferroptosis induction promoted LNM but inhibited hematogenous metastasis in mouse models. Transcriptomic and lipidomic analyses found that BACH1 repressed SCD1-mediated biosynthesis of monounsaturated fatty acids, especially oleic acid (OA). OA significantly attenuated the ferroptotic phenotypes and reversed the metastatic properties of BACH1-overexpressing cells. OA addition significantly rescued the ferroptotic phenotypes and reversed the metastatic properties of BACH1-overexpressing cells. Importantly, the concentration gradient of OA between primary lesions and the lymph resulted in the chemoattraction of tumor cells to promote invasion, thus facilitating lymphatic metastasis. BACH1-induced ferroptosis drives lymphatic metastasis via the BACH1-SCD1-OA axis. More importantly, this study confirms that ferroptosis is a double-edged sword in tumorigenesis and tumor progression. The clinical application of ferroptosis-associated agents requires a great caution.

## Introduction

Generally, tumor metastases are not randomly distributed among distant organs. Tumor cells spread via three primary routes: hematogenous metastasis, lymphogenous metastasis and peritoneal dissemination. Different tumor types and subtypes demonstrate distinct preferences for metastatic organs and routes. Peritoneal dissemination is exhibited by ovarian cancer and some subtypes of gastrointestinal cancer; it develops when cancer cells are passively released from the primary lesion and implant in the peritoneum. By contrast, most solid tumors undergo active metastasis through hematogenous and/or lymphogenous routes of dissemination. In the clinic, only a few types of solid tumors use lymphatic vessels as the first preferred metastatic route, including head and neck squamous carcinoma, esophageal cancer, skin melanoma and testicular cancer, whereas the other types of tumors have a higher propensity to metastasize via blood vessels [1].

Accumulating evidence suggests that the preference to choose specific metastatic routes can be influenced by several factors, including the circulation patterns of specific organs, cancer cell-intrinsic properties, vascular- or organ-specific niches and the host immune microenvironment [2]. Cancer cell-derived VEGFC induces angiogenesis and lymphangiogenesis and also activates the expression of multiple adhesion molecules and chemokines on lymphatic endothelial cell surfaces, which can recruit and capture specific metastatic tumor cells, leading to lymph node metastasis [3–5]. Primary tumors continuously deliver soluble factors and extracellular vesicles to progressively remodel lymph nodes from a distance [6]. Inflamed lymphatic endothelial cells in lymphatic vessels and lymph nodes build a progressively immunosuppressed microenvironment to promote metastasis [6, 7]. Epithelial–mesenchymal transition (EMT) has been clearly demonstrated to facilitate hematogenous metastasis, but it is not necessary for lymphatic metastasis [8]. Nevertheless, a recent genome sequencing study based on over 25 000 patients with primary and metastatic tumors revealed that there were no significant genomic or pathway alterations associated with organ-specific metastasis at the pan-cancer level [9]. It remains unclear why tumor cells prefer specific metastatic routes.

A recent study has revealed that the lymph appears to be a less-oxidative microenvironment than the blood; specifically, lymph has lower levels of reactive oxygen species (ROS) and free iron and higher levels of the antioxidants glutathione (GSH) and oleic acid (OA) [10]. Further analysis that was concentrated on melanoma, a typical tumor with preferential lymphatic metastasis, has shown that lymph fluid protects melanoma cells from ferroptosis and strengthens their ability to survive during secondary homogenous metastasis via incorporation of OA into phospholipids [10].

Ferroptosis is an iron-dependent form of nonapoptotic regulated cell death that occurs through excessive peroxidation of polyunsaturated fatty acids (PUFAs) during phospholipid-mediated membrane damage [11]. Thus, genes that regulate the iron content inside and outside the cell, the lipid composition in the cell membrane and oxidative stress theoretically affect the sensitivity to ferroptosis sensitivity [12, 13]. Cells are endowed with at least three antioxidant systems to mitigate lipid peroxidation, including the well-known GSH/glutathione peroxidase 4 (GPX4) axis, the ferroptosis suppressor protein 1 (FSP1)/coenzyme Q10 system and the newly reported tetrahydrobiopterin (BH4) system [12]. Transcription factors that control the oxidative stress response also contribute to ferroptosis, including the driver gene NFE2L2 and the transcription factor BTB and CNC homology 1 (BACH1) [14].

Ferroptosis has been found to contribute to various human diseases. Tumor-selective ferroptosis strengthened by the depletion of SLC7A11 delays pancreatic ductal adenocarcinoma development in transgenic mice [15]. Ferroptosis is also a predominant death mechanism underlying the effects of radiotherapy, chemotherapy and immunotherapy [12, 13, 16]. However, ferroptotic cells also release various damage-associated molecules to mediate immunogenic cell death or cause immune suppression. Thus, the pro-tumor roles of ferroptosis have attracted recent attention [17]. Ferroptosis is an important cause of chemoradiotherapy-induced injury and adverse effects. The conditional knockout of GPX4 in the KRAS-mutant mouse model was found to accelerate carcinogenesis of pancreatic cancer via the activation of inflammation in macrophages [18]. Pancreatic cancer cells succumbing to ferroptosis release KRAS mutant proteins for packaging into exosomes that are taken up by macrophages. Mutant KRAS polarizes macrophages into an M2-like protumor phenotype to promote pancreatic cancer progression [19]. Additionally, the spontaneous ferroptosis of tumor neutrophils in the tumor microenvironment limits the activity of infiltrated T cells to promote tumor growth [20]. Therefore, ferroptosis seems to be a double-edged sword in tumorigenesis.

Given recent findings, it seems that the lymph strengthens the ability of disseminated tumor cells to survive via evasion of ferroptosis, whereas blood acts as a filter to select metastatic tumor cells by inducing ferroptosis. For this mechanism, lymphogenous metastasis should be the primary metastatic route. However, hematogenous metastasis is the most important metastatic route for solid tumors in the clinic. This raises the possibility that different tumor types show distinct intrinsic characteristics and sensitivity to ferroptosis, thus actively participating in the selection of metastatic routes.

Esophageal squamous cell carcinoma (ESCC) is one of the fatal malignancies worldwide. Despite advances in early detection, surgical techniques and chemoradiotherapy methods, the prognosis for ESCC remains poor, and the 5-year survival rate is 15–25%. The prognosis of patients with lymph node metastases is often worse than that of patients without metastases. The number and proportion of metastatic lymph nodes have been shown to be important prognostic factors_._ In this study, we performed a pan-cancer analysis of known ferroptosis-related genes based on the transcriptional profiling data of The Cancer Genome Atlas (TCGA). Considering that metastatic cells entering lymph nodes are prone to release tumor antigens to induce humoral immune responses [21, 22], lymphogenous metastatic tumors are more likely to produce tumor autoantibodies at an early stage. We screened for serum autoantibodies against ferroptosis-related genes associated with early lymph node metastasis in ESCC. Comparative analysis showed that BACH1 was highly expressed in tumors with preferential lymphatic metastasis and that its serum autoantibody level was positively correlated with lymph node metastasis in ESCC. Subsequent functional and mechanistic studies deciphered the unique roles of BACH1-induced ferroptosis in lymphatic metastasis.

## Materials and methods

### Cell culture and treatment

The human ESCC cell lines KYSE30 (RRID:CVCL_1351), KYSE150 (RRID:CVCL_1348), KYSE170 (RRID:CVCL_1358), KYSE180 (RRID:CVCL_1349), KYSE410 (RRID:CVCL_1352) and KYSE510 (RRID:CVCL_1354) were generously provided by Dr. Shimada (Hyogo College of Medicine, Hyogo, Japan), and normal esophageal squamous epithelial Het-1A (CRL-2692) cells were obtained from ATCC. Cells were cultured in RPMI 1640 medium supplemented with 10% fetal bovine serum (HyClone), 100 U/mL penicillin and 100 μg/mL streptomycin at 37 °C with 5% CO_2_. All cell lines were authenticated by short tandem repeat analysis, and mycoplasma tests were negative.

cDNA encoding the human BACH1 gene (NM_206866) was synthesized by PCR and then cloned into a lentiviral vector, pLVS-IRES-RFP (ViGene Biosciences, Shandong, China). Two short hairpin RNAs (shRNA-1: TATGCACAGAAGATTCATAGG and shRNA-2: ATATCATGGATACAATCCAGC) targeting BACH1 were inserted into the plent-U6-GFP-Puro vector (ViGene). Subsequently, 4×10^5^ KYSE150 and KYSE170 cells were plated in 6-well plates and cultured until they reached 80% confluence. KYSE150 cells were transfected with a BACH1-expressing pLVS-IRES-RFP vector, and KYSE170 cells were transfected with plent-U6-GFP-Puro containing BACH1-targeting shRNA. After 48 h, cell culture medium containing 4 μg/mL puromycin was added to select transfected cells with stably overexpressed BACH1 (KYSE150-BACH1-OE) or knocked down BACH1 (KYSE170-BACH1-KD).

### Sample collection

Serum samples were collected from the Cancer Hospital of the Chinese Academy of Medical Sciences from February 2009 to June 2017, and all samples were stored at −80 °C until use. A total of 12 healthy controls, 12 patients with early-stage ESCC without lymph node metastasis (ESCC w/o LNM, T1N0M0) and 12 patients with early-stage ESCC with lymph node metastasis (ESCC w/ LNM, T1N1M0) were first selected for autoantibody screening (Supplementary Table S1). Then, additional serum samples were collected from 50 independent patients with ESCC w/o LNM patients, 78 patients with ESCC w/ LNM patients and 122 healthy controls (Supplementary Table S2) for validation by immunoblot analysis.

### RNA extraction, RT–PCR and quantitative PCR (qPCR)

Total RNA was extracted using TRIzol reagent (Cat# 15596018, Invitrogen), and reverse transcription was performed from 2 µg of total RNA utilizing a HiFiScript cDNA synthesis kit (CW2569M, CWbiotech). qPCR was conducted on an ABI QuantStudio 5 device (Applied Biosystems) using the SYBR Green dye and a real-time PCR master mix (RP420A, Takara). Reactions were performed in triplicate and β-actin was used as a control. Relative mRNA expression levels were determined according to the cycle threshold (Ct) and were normalized to ACTB levels using the 2^-ΔΔCt^ formula. The primers used are listed in Supplementary Table S3.

### Western blot analysis

Cells were lysed with RIPA buffer (50 mM Tris-HCl, 150 mM NaCl, 1.0% NP-40, 0.5% sodium deoxycholate, and 0.1% SDS) containing a protease inhibitor cocktail (Cat# 04693459001, Roche) and were centrifuged at 12,000 rpm for 15 min to remove debris. Total protein (25 μg/well) was separated by 4%-20% gradient SDS–PAGE gels, and the separated proteins were transferred to PVDF membranes. After blocking in 5% nonfat dry milk in PBS containing 0.1% Tween 20 (PBST), the membranes were incubated with primary antibodies at 4 °C overnight. The proteins were detected with horseradish peroxidase (HRP)-conjugated secondary antibodies and visualized with Renaissance Plus Reagent (Cat# 93702, CST). The antibodies used are listed in Supplementary Table S4. Full and uncropped western blots are presented in the Supplemental Material.

### Screening serum autoantibody profiling by HuProt proteome array

HuProt proteome arrays (CDI Laboratories, Baltimore, MD) were employed to screen serum autoantibodies of ESCC patients. Each HuProt array is composed of 19 394 individual purified full-length human proteins that are GST-tagged at their N termini and printed in duplicate on a glass substrate (OPEpoxySlide). Thus, the quality of the protein arrays can be verified based on the fluorescence intensity of anti-GST signals detected with anti-GST antibodies and subsequent staining with a Cy5-labeled secondary antibody. The number of detected proteins and consistency of duplicate protein signals were adapted to control the microarray quality.

For serum profiling, each HuProt array was blocked with 3% w/v BSA (Sigma) in PBST buffer for 1.5 h. Next, 300 μL of 1000-fold diluted serum sample (in PBST buffer with 3% w/v BSA) was added, and the array was incubated at 4 °C overnight with gentle shaking. The arrays were then washed in PBST and incubated with 300 μL of Cy3-conjugated goat anti-human IgG and Cy5-conjugated goat anti-human IgM (1:1000; Jackson Laboratory) at room temperature for 1 h with gentle shaking. After rinsing with PBST buffer followed by deionized water, the slides were dried and scanned with a LuxScan-10K Scanner (CapitalBio, Beijing, China).

The image processing and data acquisition steps for the arrays were performed using GenePix Pro 6.0 (Molecular Devices). The mean of the median foreground to the median background signal ratio was calculated for each spot on the arrays. The foreground median intensity divided by the local background median intensity was defined as the signal value, and the mean signal value of each duplicate pair was taken as the antibody’s signal value. Next, within-chip normalization was performed. A positive control with human IgG and IgM and a negative control with GST were used for quality control. The reproducibility was determined to demonstrate interassay consistency, and a high correlation coefficient of R^2^ = 0.93 was obtained for duplicate spots. Candidate autoantibodies were selected according to the threshold fold change>1.5 and high discriminatory ability, which was defined by the mean values of sensitivity and specificity.

### Immunoblot assay of serum autoantibodies

Two micrograms of a recombinant BACH1 protein with a C-terminal DDK tag (TP321628, OriGene) was electrophoresed on SDS–PAGE gels. The proteins were then transferred to PVDM membranes (Millipore) by electroblotting. The membranes were sliced into strips after blocking with nonfat dry milk in PBST (5%, w/v). Each strip containing BACH1 protein was incubated with ESCC serum or anti-DDK-tagged mouse antibody (M185-3L, MBL) as a loading control at 4 °C overnight. The proteins were detected with HRP-conjugated anti-human IgG (ZB-2304, ZSGB-BIO) or anti-mouse IgG (Cat# 7076 S, CST) for 1 h at room temperature and visualized with MaxiLumin Femtogram chemiluminescent substrate (WB002, Biokits). The intensity of the bands in each strip was analyzed using the ImageJ software. The sample intensity divided by the loading control intensity was defined as the relative signal intensity of each band.

### Immunohistochemistry (IHC)

Human ESCC tissue microarrays (Outdo Biotech and Superbiotek, Shanghai, China) containing tumor and corresponding adjacent normal tissues were analyzed by IHC. Briefly, the tissue sections were incubated overnight at 4 °C with anti-BACH1 (1:200, Cat# 5393, Abclonal) or anti-SCD (1:400, ab236868, Abcam) antibodies after deparaffinization and rehydration. No primary or nonspecific antibody was included as a negative control. Antibody binding was detected with a Polink-2 Plus HRP Rabbit Polymer Kit (pv-6001, Golden Bridge), and images were captured with Aperio ScanScope SC software (v8.0, Vista). The results were independently evaluated and scored by two senior pathologists who were blinded to the sample identities. Quantification was based on the percentage of positive cells and staining intensity [23]. In summary, the BACH1 or SCD1 staining intensity scores (0, negative; 1, weak; 2, moderate; and 3, strong) and staining area scores (0, <5%; 1, 5–25%; 2, 26–50%; 3, 51–75%; and 4, ≥75%) were multiplied to generate a total score. A total score of ≤6 or >6 was considered to indicate low or high expression of the targeted proteins, respectively.

### Animal experiments and histological assessments

Animals were housed in a pathogen-free facility, and all experimental procedures were approved by the Institutional Animal Care and Use Committee (IACUC) at the Cancer Hospital of the Chinese Academy of Medical Sciences. The sample size for animal studies had an adequate power to detect a prespecified effect size. For ferroptosis interventions, 1×10^6^ KYSE150-BACH1-OE or control cells were pretreated with 1 µM RSL-3 (Cat# s8155, Selleckchem) for 6 h, and were then injected into 5-week-old female BALB/c nude mice (Huafukang Bioscience) (*n* = 9 per group). The tumor size and volume were measured every 3 days. After 17 days, the mice were euthanized. The xenograft tumors were weighed and fixed with 4% paraformaldehyde for hematoxylin and eosin (H&E) staining and IHC.

For the in vivo lymph node metastasis assay, 5×10^5^ KYSE150-BACH1-OE, KYSE170-BACH1-KD or corresponding control cells were injected into the footpads of 5-week-old female BALB/c nude mice (Huafukang Bioscience) (*n* = 8 per group). Four weeks after injection, the mice were euthanized, and their popliteal lymph nodes were excised and embedded in paraffin. The tissue samples were then stained with H&E and subjected to IHC. For ferroptosis intervention, 2.5×10^5^ KYSE150-BACH1-OE cells were injected into the footpads of 18 BALB/c nude mice until the tumor was visible to the naked eye. Next, the mice were randomly divided into 3 groups by weight and intraperitoneally injected with 30 mg/kg RSL-3 (Cat# s8155, Selleckchem) (*n* = 6), 10 mg/kg liproxstatin-1 (Cat# s7699, Selleckchem) (*n* = 6) or 1% DMSO in PBS (*n* = 6) as a vehicle control once daily for 20 days. After 20 days, the mice were euthanized, and their popliteal lymph nodes were excised and embedded in paraffin for future analysis.

For the in vivo lung metastasis assay, 5×10^5^ KYSE150-BACH1-OE or corresponding control cells were suspended in 100 μL of PBS and injected into the tail veins of 5-week-old female NOD-Prkdc^em26^Il2rg^em26^Nju (NCG) mice (GemPharmatech, Jiangsu, China) (*n* = 6 per group). For ferroptosis intervention with OA, 5×10^5^ KYSE150-BACH1-OE cells, with or without 400 µM OA (Cat# O1383, Sigma–Aldrich) pretreatment were injected into the tail veins of NCG mice (*n* = 6 per group). The body weights of the mice were measured every 3 days, and the mice were euthanized at 17 days after the injection. The lungs were excised by dissection to assess metastasis using an IVIS Lumina XRMS In Vivo Imaging System (PerkinElmer).

### Flow cytometry analysis

To analyze the changes in intracellular lipid ROS levels, cells were treated with 2 μM C11-BODIPY^591/581^ (Cat# D3861, Thermo Fisher) for 30 min. Then, the cells were digested with trypsin, collected and resuspended in PBS containing 2% FBS. The levels of lipid peroxides in cell membranes were measured by flow cytometry based on the fluorescence emission peak of the C11-BODIPY^591/581^ probe after oxidation shifts from ~590 nm to ~510 nm.

### Cell viability assay

Cells were seeded into 96-well plates and treated with a ferroptosis inducer or inhibitor when the confluence reached 80%. Subsequently, 100 µL of serum-free medium containing 10 µL of Cell Counting Kit-8 (CCK-8) reagent (Cat# KGA317, KeyGEN) was added to each well, and the cells were incubated for 2 h. Finally, the absorbance at 450 nm was measured using a microplate reader to evaluate cell viability. The half-maximal inhibitory concentration (IC_50_) value was defined as the drug concentration that inhibited 50% of cell growth compared with that in the untreated control. This value was calculated using the GraphPad Prism software (San Diego) according to the following formula: cell proliferation rate (%) = (OD 450 treatment/OD 450 control) × 100%. This experiment was performed in triplicate, and the results are presented as the mean ± SEM.

### Untargeted lipidomic analysis

In brief, KYSE150-BACH1-OE (BACH1-OE) and KYSE150-vector (vector) cells were collected, frozen in liquid nitrogen and thawed 3 times and sonicated on ice. Then, MTBE:MEOH = 5:1 was added to the samples, followed by incubation at −40 °C for 1 h. After centrifugation at 900× *g* for 15 min at 4 °C, 300 μL of supernatant was transferred to a fresh tube and dried in a vacuum concentrator at 37 °C. The dried samples were reconstituted in 150 μL of 50% methanol in dichloromethane by sonication on ice for 10 min. The constitution was then centrifuged at 16200 ×*g* for 15 min at 4 °C, and 75 μL of supernatant was transferred to a fresh glass vial for LC/MS analysis. The quality control (QC) sample was prepared by mixing equal aliquots of the supernatants from all samples. LC–MS/MS analyses were performed using an UHPLC system (1290, Agilent) equipped with a Kinetex C18 column (2.1 × 100 mm, 1.7 μM, Phenomen). A QE mass spectrometer (Q Exactive Orbitrap, Thermo Fisher Scientific) was used because of its ability to acquire MS/MS spectra in data-dependent acquisition (DDA) mode via control of the acquisition software (Xcalibur 4.0.27, Thermo).

The raw data files were converted to mz XML format using the ‘msconvert’ program from ProteoWizard. Peak detection was first applied to the MS1 data. The CentWave algorithm in XCMS was used for peak detection with the MS/MS spectrum, and lipid identification was achieved through a spectral match using the LipidBlast library (https://www.lipidmaps.org/). Different phospholipid compositions between different groups were defined by the absolute log2-fold change, R1, and the threshold variable importance in the projection (VIP) (VIPR1) value from the OPLS-DA model. A Student’s *t* test was used to assess the significance of differences (*P* < 0.05) in the abundances of metabolites.

### Targeted lipidomic analysis

To identify and quantify lipid species, comprehensive targeted quantitative lipidomic analysis was performed with an ISQ 7000 mass spectrometer (Thermo Fisher Scientific) using multiple reaction monitoring (MRM) by Bioprofile Biotech Ltd. (Shanghai, China). BACH1-OE and vector cells were pretreated with serum-free medium for 12 h to eliminate the interference of exogenous fatty acids. A total of 1×10^7^ cells were collected and immediately inactivated with liquid nitrogen. The samples were added to 2 mL of 1% sulfuric acid-methanol mixed solution under vortex mixing for 1 min. Then, the samples were esterified for half an hour in a water bath at 80 °C. The supernatant was washed with 5 mL of pure water. To remove the water, 500 μL of the upper organic layer was taken and spiked with 100 mg of anhydrous sodium sulfate. Then, 25 μL of internal standard (IS) was added, and the mixture solution was transferred into a GC vial for GC–MS analysis. A calibration curve was constructed, and the concentrations of the corresponding species were calculated by plotting the analyte-to-IS peak area ratio against the theoretical concentration of each individual fatty acid. The experiments were performed with three independent biological replicates. To analyze the significance of the fold difference in each lipid species between the two conditions, we performed a paired two-tailed Student’s *t* test.

### RNA sequencing analysis

Total RNA was extracted using TRIzol reagent (Cat# 15596018, Invitrogen) and stored at −80 °C. Each sample was prepared in triplicate. After purification, the RNA sequencing library was prepared using an Ultra RNA Library Prep Kit (New England Biolabs, Ipswich, MA). Initial quantification was performed using a Qubit 2.0 fluorometer. Then, the library was diluted to 1.5 ng/μL, and the insert size was determined using an Agilent 2100 bioanalyzer. When the insert size met the criteria, the effective library concentration (≥10 nM) was accurately quantified with a CFX 96 fluorescence quantitative PCR instrument (Bio-Rad). The qualified library was sequenced with an Illumina platform using the PE150 sequencing strategy.

### Chromatin immunoprecipitation (ChIP)-PCR assay

The ChIP assay was performed using the SimpleChIP enzymatic chromatin IP kit (Cell Signaling Technology) according to the manufacturer’s instructions. A total of 1×10^7^ KYSE170 cells were used for each ChIP assay, and chromatin DNA was precipitated with an anti-BACH1 antibody (Cat# AF5776, R&D Systems) or normal rabbit IgG. The immunoprecipitated DNA and the input samples were analyzed by PCR using the primers shown in Supplementary Table S3.

### Dual-luciferase reporter assay

The DNA fragments SCD1-Full (hg38, chr10:100348450–100351450), SCD1-1 (chr10:100348450–100349166) and SCD1-2 (chr10:100350099–100350822) or the mutant vectors (SCD1-Mut1, SCD1-Mut2 and SCD1-Mut 3) from the human SCD1 (NM_005063.5) intron 2 sequence were inserted into the pGL3-basic vector (Promega) to construct the pGL3-SCD1 reporters, respectively. Cells were plated into 24-well plates at 5 × 10^4^ cells per well, and the pGL3-SCD1 reporters, pRL-TK and the BACH1 overexpressing plasmid or siRNAs were cotransfected into cells in each well. After 48 h, luciferase activity was measured using a dual-luciferase reporter assay system (Promega) following the manufacturer’s protocol. The luciferase activity was normalized to that of Renilla luciferase activity for each comparison. All experiments were performed in triplicate, and the results are presented as the mean ± SEM.

### Statistical analysis

Statistical analysis was performed using GraphPad Prism v8.0 software (GraphPad Software, San Diego, CA). All quantitative data are presented as the mean ± SEM. Differences between two groups were compared by the two-tailed Student’s *t* test or Mann–Whitney rank test. Differences among three or more groups were compared by two-tailed analysis of variance (ANOVA) followed by multiple comparisons tests. The qualitative data were compared using the chi-square test. Survival analyses were performed using Kaplan–Meier curves combined with the log-rank test. Receiver operating characteristic (ROC) curve analysis was performed to assess the diagnostic performance. A *P* value of less than 0.05 was considered to indicate statistical significance.

This study was approved by the Institutional Review Board of the Ethics Committee of National Cancer Center/Cancer Hospital, Chinese Academy of Medical Sciences and Peking Union Medical College (No. NCC1783) and performed in accordance with the guidelines of the Declaration of Helsinki.

## Results

### Pan-cancer analysis indicates distinct expression patterns of ferroptosis-related genes in different types of tumors

We extracted experimentally validated ferroptosis-related genes from the FerrDB database and divided them into three categories, including 124 driver genes, 106 suppressor genes and 9 genes with dual roles. Subsequently, we downloaded the transcriptional profiles for 8247 RNA sequencing samples covering 17 common cancers from TCGA. Except for skin cutaneous melanoma (SKCM), the remaining tumor types had over 10 paracancerous tissue samples. The SKCM dataset included 368 metastatic lesion samples and 103 primary samples. Thus, the differences between metastases and primary tumors were compared for SKCM, whereas differences between tumors and paracancerous tissues were analyzed for the remaining tumor types. The expression data for ferroptosis-related genes in various tumors were extracted, and only differentially expressed genes were retained for further analysis.

**Fig. 1:**
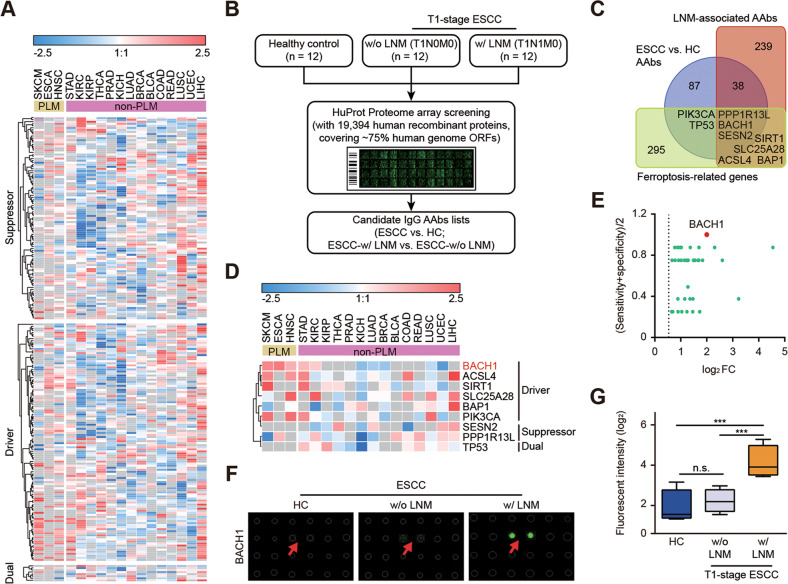
Expression patterns of ferroptosis-related genes in tumors with preferential lymphatic metastasis or nonlymphatic/hematogenous metastasis. **A** Expression patterns of ferroptosis-related genes in tumors with preferential lymphatic metastasis or nonlymphatic/hematogenous metastasis. The red color indicates overexpression in respective tumors, and the sky-blue color indicates downregulation in respective tumors, whereas grey indicates no difference between tumor and nontumor tissues (FDR-adjusted Wilcox test). The blocks beneath the TCGA RNA sequencing datasets indicate the classifications of metastatic types. Some key ferroptosis regulatory genes are labeled; among them, BACH1 was overexpressed in tumors with preferential lymphatic metastasis (PLM). SKCM, skin cutaneous melanoma (metastatic lesions vs. primary tumors); ESCA, esophageal carcinoma; HNSC, head and neck cancer; STAD, stomach adenocarcinoma; KIRC, kidney renal clear cell carcinoma; KIRP, kidney renal papillary cell carcinoma; THCA, thyroid carcinoma; PRAD, prostate adenocarcinoma; KICH, kidney chromophobe; LUAD, lung adenocarcinoma; BRCA, breast invasive carcinoma; BLCA, bladder urothelial carcinoma; COAD, colon adenocarcinoma; READ, rectum adenocarcinoma; LUSC, lung squamous cell carcinoma; UCEC, uterine corpus endometrial carcinoma; LIHC, liver hepatocellular carcinoma; PLM, tumors with preferential lymphatic metastasis; non-PLM, tumors with preferential nonlymphatic metastasis. **B** Whole-proteome screening for serum autoantibodies (AAbs) associated with the tumor and lymph node metastasis (LNM) in esophageal squamous cell carcinoma (ESCC). We employed a discovery strategy to identify serum AAb candidate biomarkers for early-stage ESCC using a proteome array-based approach. The screened serum samples were collected from healthy controls (HC), T1-stage ESCC patients without lymph node metastasis (w/o LNM, T1N0M0) and T1-stage ESCC patients with lymph node metastasis (w/ LNM, T1N1M0). These sera were loaded onto a human proteome microarray of 19,394 recombinant proteins to acquire an AAb repertoire in response to ESCC and LNM. ORF, open reading frame. **C** Overlapping genes among the three groups (ESCC vs. HC AAbs, LNM-associated AAbs and ferroptosis-related genes). BACH1 was the only shared gene among the three groups. **D** Heatmap of overlapping genes in in tumors with preferential lymphatic metastasis or nonlymphatic/hematogenous metastasis. **E** The discriminatory ability (sensitivity + specificity) and fold changes of the upregulated AAbs on the HuProt arrays in the IgG channel. Each dot represents an AAb. Anti-BACH1 AAbs completely distinguished patients with LNM from those without LNM. **F** Typical anti-BACH1 IgG AAb scanning image and fluorescence intensity quantification of the microarray. **G** BACH1 showed a strong anti-human IgG signal in patients with ESCC w/ LNM but a weak signal in healthy controls (HC) and patients with ESCC w/o LNM.

Unsupervised clustering analysis showed that the expression patterns of ferroptosis-related genes in tumors with preferential lymphatic metastasis (with lymph node metastasis as the first metastatic route), including SKCM, esophageal carcinoma (ESCA) and head and neck squamous cell carcinoma (HNSC), were distinct from those in tumors with preferential nonlymphatic metastasis (Fig. 1A). A small group of genes, including BACH1, were overexpressed in all three lymphatic metastasis-dominant tumors. Importantly, there was substantial heterogeneity among hematogenous metastatic tumors. Some tumors, such as kidney cancer, thyroid cancer, and prostate cancer, showed a trend of low expression of both ferroptosis suppressor and driver genes, while other tumors, such as liver cancer, endometrial cancer, and lung squamous cancer, showed a trend of high expression of both ferroptosis suppressor and driver genes. These results suggest that the different ferroptosis sensitivities may be intrinsic features of tumors that preferentially use different metastatic routes.

### Screening for serum autoantibodies associated with lymph node metastasis in patients with early-stage ESCC

ESCC is a typical tumor that prefers the lymphatic metastasis route, which indicates that metastatic cells have earlier and greater opportunities to contact immune cells in lymph nodes to stimulate humoral immunity. We first screened for serum autoantibodies in patients with early-stage ESCC lymph node metastasis. Sera from patients with T1-stage ESCC (i.e., the depth of tumor invasion does not exceed the submucosa), with different statuses of lymph node metastasis and healthy controls were loaded on HuProt proteome arrays, covering approximately 75% of the human proteome. Differential spots were identified using the following criteria: the positive rate in the tumor group of over 50%, and the minimum signal value in the tumor groups greater than the median in the control group. The protein spots with a fold change of greater than 1.5 in the average signal value between the tumor and healthy control groups or between the T1N0M0 and T1N1M0 groups were considered candidate autoantigens (Fig. 1B). Thus, 130 and 284 IgG autoantibodies were identified as being potentially associated with ESCC (Supplementary Table S5) and lymph node metastasis (Supplementary Table S6), respectively. Additionally, 41 serum autoantibodies were highly expressed in all ESCC cases, especially in those with lymph node metastasis (Supplementary Table S7).

### Autoantibodies against the ferroptosis-related gene BACH1 appear early in ESCC patients with lymph node metastasis

Comparison of these two differential autoantibody datasets with the list of ferroptosis-related genes revealed only nine ferroptosis-related proteins that generated serum autoantibodies in ESCC. PPP1R13L, BACH1 and SESN2 were shared by all three datasets (Fig. 1C). We subsequently evaluated the expression characteristics of the nine genes by pan-cancer analysis (Fig. 1D). Only BACH1 showed significant overexpression in tumors with preferential lymphatic metastasis, whereas it was downregulated in most tumors with preferential nonlymphatic metastasis.

Serum autoantibodies against BACH1 showed the highest discriminative capability for lymph node metastasis in ESCC. Regarding the anti-BACH1 test, both the sensitivity and specificity reached 100% in the discovery cohort (Fig. 1E; Supplementary Table S7). The fluorescence intensity was extremely low in healthy individuals and weak in ESCC patients without lymph node metastasis. However, its average level was increased fourfold in patients with lymph node metastasis (Fig. 1F, G). Taken together, the findings suggest that BACH1 is a specific mediator of lymphatic metastasis.

### Independent validation of anti-BACH1 autoantibodies in ESCC

**Fig. 2:**
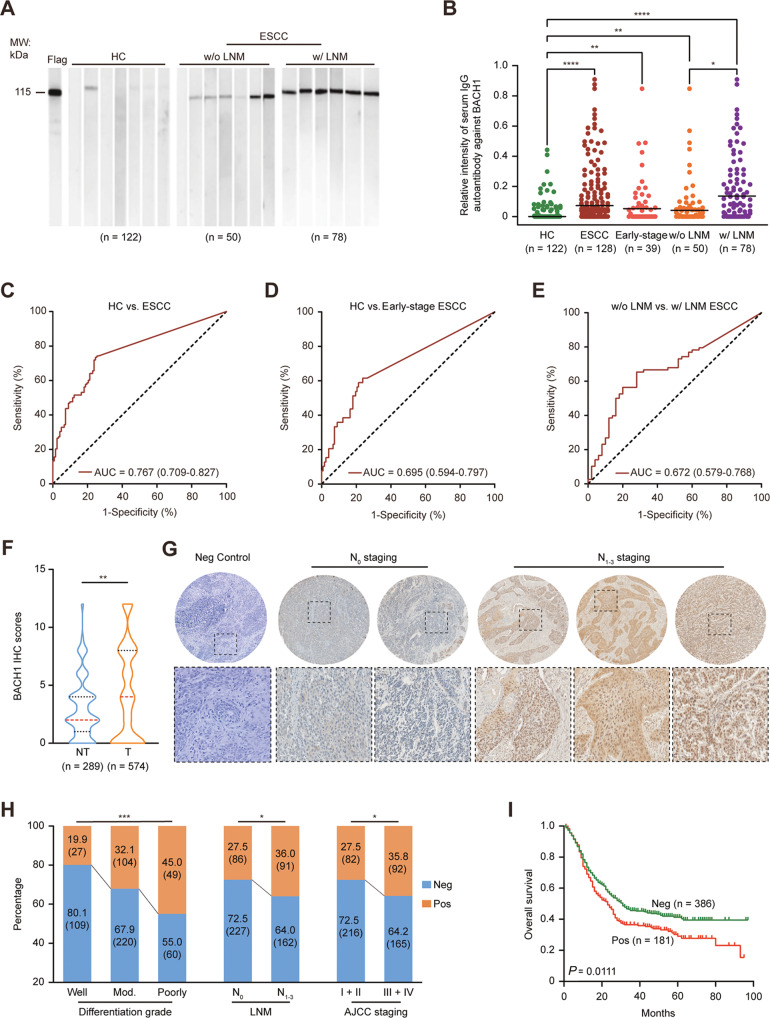
Validation of serum anti-BACH1 autoantibodies and BACH1 antigen in ESCC. **A** BACH1 recombinant proteins were separated by SDS–PAGE, transferred onto PVDF membranes and then incubated with a diluted anti-DDK-flag antibody or human serum. DDK-flag served as a positive control and internal loading control; representative data of immunoblotting experiments are shown. **B** Scatter plot showing the relative intensity of serum anti-BACH1 autoantibodies (AAbs) in healthy controls (HC, *n* = 122) and ESCC patients, including those at an early stage (*n* = 39), without lymph node metastasis (w/o LNM, *n* = 50) and with lymph node metastasis (w/ LNM, *n* = 78). **C-E** Receiver operating characteristic (ROC) curve analysis of the anti-BACH1 AAb levels in HC vs. ESCC, HC vs. early-stage ESCC and ESCC w/o LNM vs. ESCC w/ LNM. The areas under the curve (AUCs) were 0.767, 0.695 and 0.672 for anti-BACH1 AAb-mediated detection of ESCC, early-stage ESCC and ESCC w/ LNM, respectively. **F** The violin plot shows the immunohistochemistry (IHC) scores of BACH1 in nontumor (NT, n = 289) and tumor (T, *n* = 574) tissues. **G** Representative images of BACH1 and negative control staining in the tumor tissues of N0-stage and N1- to N3-stage ESCC. Upper panels, ×40; lower panels, ×200. **H** Positive BACH1 expression was significantly correlated with differentiation grade, lymph node metastasis and the American Joint Committee on Cancer (AJCC) staging in ESCC. Mod, moderate differentiation. **I** Kaplan–Meier curve of overall survival according to BACH1 levels in tumor samples (*n* = 567). The log-rank test was performed. Neg, negative; Pos, positive; *P < 0.05, **P < 0.01, ***P < 0.001.

We validated the anti-BACH1 autoantibody findings by immunoblotting using a larger independent cohort (Fig. 2A), including 122 healthy controls, 39 early-stage ESCC patients, 50 ESCC patients without lymph node metastasis, and 78 ESCC patients with lymph node metastasis. The relative anti-BACH1 autoantibody intensity was significantly higher in patients with ESCC than in healthy controls, especially with early-stage ESCC, than in healthy controls (Fig. 2B). Importantly, it was also significantly higher in patients with lymph node metastasis than in those without lymph node metastasis (Fig. 2B). When the cut-off value was determined as the mean intensity + 3 standard deviations (SDs) in the healthy control group, the anti-BACH1 autoantibody-positive percentage was 2.5% (3/122) in healthy controls, 24.8% (30/128) in ESCC patients, 12.8% (5/39) in early-stage ESCC patients, 12% (6/50) in ESCC patients without lymph node metastasis and 30.8% (24/78) in ESCC patients with lymph node metastasis (Supplementary Table S8). The anti-BACH1 autoantibody level was significantly associated with lymph node metastasis (*P* = 0.0145) and the American Joint Committee on Cancer (AJCC) stage (*P* = 0.0012); however, there was no correlation with age, sex, histological grade, tumor location, tumor size, T stage or M stage (Supplementary Table S9).

Receiver operating characteristic curve analysis was conducted to evaluate the performance of the anti-BACH1 autoantibody for detecting ESCC, early-stage ESCC and ESCC with lymph node metastasis. The areas under the curve (AUCs) were 0.767, 0.695 and 0.672, respectively (Fig. 2C–E). With a specificity of 95%, the sensitivity values were 32.8%, 20.5% and 14.1%, respectively (Supplementary Table S8).

### Elevated BACH1 protein levels in ESCC tissues are associated with lymph node metastasis

To investigate whether elevated anti-BACH1 serum autoantibody levels reflect increased antigen levels in ESCC tumor tissues, we performed an immunohistochemical staining assay of BACH1 using tissue microarrays. The positive rates of BACH1 in the adjacent nontumor and tumor tissues were 9.7% (28/289) and 31.5% (181/574), showing significant overexpression of BACH1 in ESCC (Fig. 2F). Importantly, the BACH1 protein was upregulated in tumors with N1-3 staging compared with those with N0 staging (Fig. 2G, H), which may be the underlying cause of the elevated serum anti-BACH1 autoantibody levels. Moreover, high BACH1 expression was significantly positively correlated with the differentiation grade, AJCC staging and a poor prognosis of ESCC (Fig. 2H, I; Supplementary Table S10). The median overall survival times of the positive and negative expression groups were 23 and 31 months, respectively.

### BACH1 induces ferroptosis in ESCC cells

**Fig. 3:**
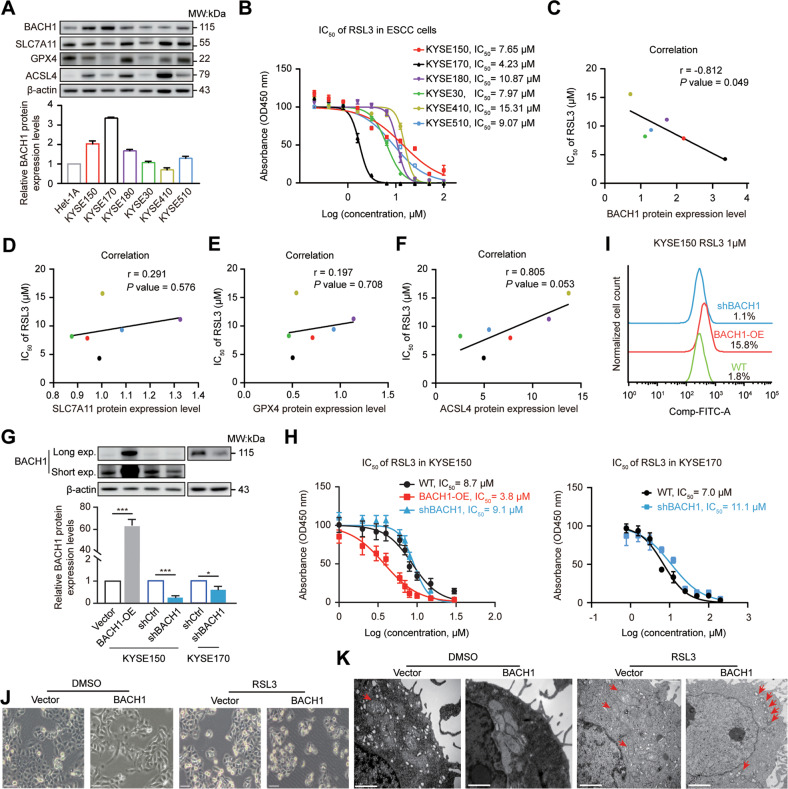
BACH1 affects the sensitivity of ESCC cells to ferroptosis. **A** Relative expression of the BACH1, SLC7A11, GPX4, and ACSL4 proteins in esophageal epithelium Het-1A and ESCC (KYSE150, KYSE170, KYSE180, KYSE30, KYSE410 and KYSE510) cells as determined by western blotting. β-actin was used as a loading control. **B** Concentration–response plot of different ESCC cells to RSL3, a ferroptosis-triggering agent, as determined by CCK-8 assay with measurement of the absorbance at 450 nm. **C-F** Correlations between BACH1 (**C**), SLC7A11 (**D**), GPX4 (**E**), and ACSL4 (**F**) protein expression levels (**A**) and their relevant IC_50_ values of RSL3 (**B**) against ESCC cells. **G** Lentivirus-mediated stable ectopic expression (BACH1-OE and shBACH1) and knockdown (shCtrl and shBACH1) of BACH1 in KYSE150 and KYSE170 cells. The efficiency of overexpression and knockdown was determined by western blotting. Long and short exposures (exp.) of the BACH1 blots are shown for KYSE150 cells. **H** Forced expression of BACH1 decreased the IC_50_ value of RSL3 against KYSE150 cells (left panel), whereas knockdown BACH1 knockdown increased the IC_50_ value of RSL3 against KYSE170 cells (right panel). **I** Flow cytometric analysis after C11-BODIPY^591/581^ staining showed the changes in lipid peroxidation after transient perturbation of BACH1 followed by treatment with 1 µM RSL3 in KYSE150 cells. **J** Phase-contrast microscopic changes in cellular shape in the absence (DMSO) or presence of 1 µM RSL3 for 6 h. **K** Ultrastructural changes in vector control and BACH1-OE cells as determined through transmission electron microscopy after incubation in the absence or presence of 1 µM RSL3 for 6 h. The red arrows indicate mitochondria. Scale bar, 0.5 μm.

We first detected BACH1, SLC7A11, GPX4 and ACSL4 protein expression and the sensitivity to the ferroptosis inducer RSL3 in six ESCC cell lines (KYSE150, KYSE170, KYSE180, KYSE30, KYSE410 and KYSE510) (Fig. 3A, B). The results revealed that BACH1 expression was significantly negatively correlated with the IC_50_ values of RSL3 against ESCC cells (Fig. 3C), whereas the other proteins showed no significant associations (Fig. 3D–F).

Next, we constructed two stable BACH1-knockdown KYSE170 and KYSE150 cell lines and an ectopic BACH1-expressing KYSE150 cell line (Fig. 3G). The IC_50_ values of RSL3 were measured against KYSE150 and KYSE170 cells with different BACH1 expression levels, and the results showed that ectopic expression (BACH1-OE) decreased while stable knockdown (shBACH1) increased the IC_50_ values compared with those against wild-type (WT) cells (Fig. 3H). Flow cytometric analysis after C11-BODIPY^591/581^ staining indicated that compared with the WT cells (1.8%), the lipid ROS percentage in BACH1-OE cells increased to 15.8%, while that in shBACH1 cells decreased to 1.1% (Fig. 3I). Under a phase-contrast microscope, BACH1-OE cells became round and floated after the addition of 1 μM RSL3, whereas the control cells were not significantly affected (Fig. 3J). Furthermore, transmission electron microscopy indicated that BACH1-OE cells in the presence of 1 μM RSL3 exhibited fewer mitochondria, greater membrane density, and fewer cristae than control cells (Fig. 3K), consistent with the morphological changes of ferroptosis [11, 13]. Collectively, these findings indicate that BACH1 increases the sensitivity of ESCC cells to ferroptosis.

### BACH1 facilitates lymph node metastasis but inhibits hematogenous metastasis in ESCC in vivo

**Fig. 4:**
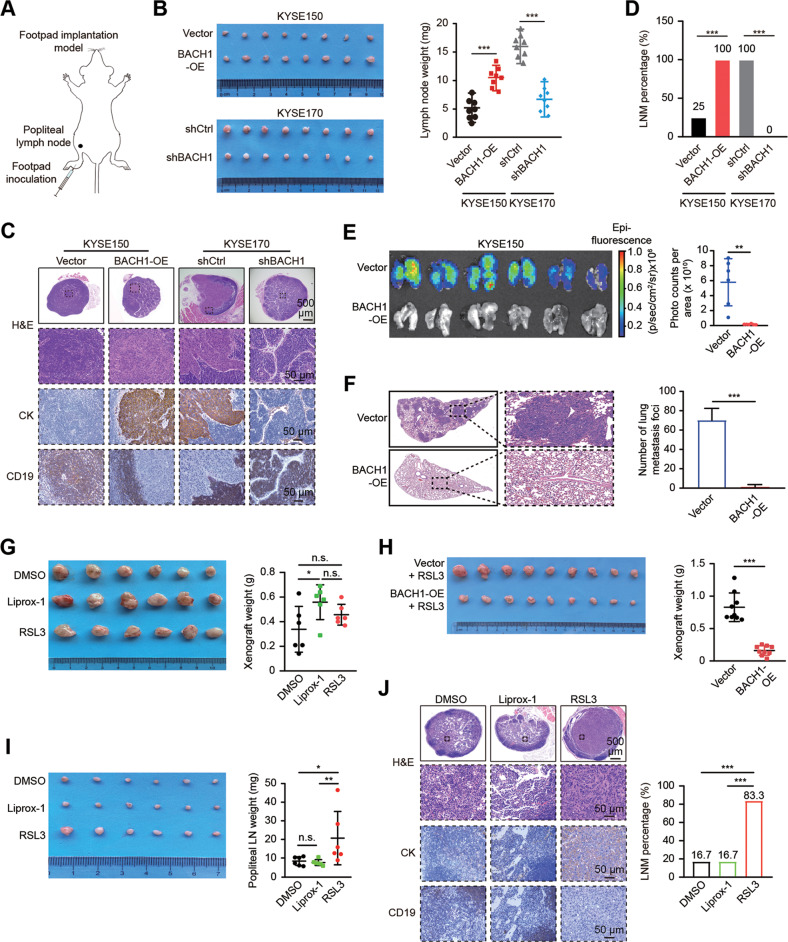
Distinct roles of BACH1-induced ferroptosis in lymphatic and hematogenous metastasis in vivo. **A** Schematic diagram of the footpad implantation nude mouse model. Cells were injected into the footpads on the left side of BALB/c nude mice, and after 4 weeks, the ipsilateral draining popliteal lymph nodes (LNs) of the mice were dissected and examined. **B** Images of popliteal LNs removed from the indicated groups (*n* = 8). The weights of the popliteal LNs were measured and analyzed. **C** Histological examination of popliteal LNs with hematoxylin and eosin (H&E) and immunohistochemical (IHC) staining. Representative results of the indicated groups are shown. Scale bars: upper, 500 μm; lower, 50 μm; Cytokeratin (CK), an epithelial marker; CD19, a B-lymphocyte marker. **D** Quantitative analysis of the metastatic positive percentage in all popliteal LNs in the indicated groups. LNM, lymph node metastasis. **E** Bioluminescence imaging and quantification of photon flux of lung metastatic lesions from NCG mice with intravenous injection of 2.5 × 10^5^ KYSE150-vector or KYSE150-BACH1-OE cells for 20 days. **F** Representative H&E staining and quantitative analysis of lung metastatic foci of the dissected lungs from the NCG mice in panel (**E**). **G** Images of subcutaneous xenografts removed from the indicated groups. The ferroptosis inhibitor liproxstatin-1 (Liprox-1) promoted the growth of subcutaneous xenografts, whereas the ferroptosis inducer RSL3 had no significant effect. KYSE150-BACH1-OE cells (1 × 10^6^) were subcutaneously transplanted into the flanks of nude mice (*n* = 18). After gross observation of tumor formation on day 12, tumor xenografts were established, and the mice were randomly divided into three groups by weight (6 mice per group). Each group of tumor-bearing mice was intraperitoneally injected with control (DMSO), Liprox-1 (30 mg/kg) or RSL3 (30 mg/kg) every two days for 20 days. Xenograft tumors were then removed for imaging and weighing. **H** Images of subcutaneous xenografts removed from the indicated groups (*n* = 18). Pretreatment with 1 µM RSL3 for 6 h significantly inhibited the subcutaneous growth of KYSE150-BACH-OE cells in nude mice compared with that in the vector groups. **I** Images of popliteal LNs removed from the indicated groups. The ferroptosis inducer RSL3 promoted the metastasis of popliteal LNs. A total of 5 × 10^5^ KYSE150-BACH1-OE cells were inoculated into the footpads of nude mice (n = 18). After 10 days, the mice were randomly divided into three groups by weight (6 mice per group). DMSO, Liprox-1 (30 mg/kg) or RSL3 (30 mg/kg) was intraperitoneally injected into each mouse in the three groups every two days for 20 days. Popliteal LNs were then removed for imaging and weighing, which demonstrated that the LNs in the RSL3 group were larger and heavier than those in the DMSO and Liprox-1 groups. **J** Representative images of H&E and IHC staining of popliteal LNs and quantitative analysis of the metastatic positive percentage in the indicated groups. CK cytokeratin, LNM lymph node metastasis; *, *P* < 0.05; **, *P* < 0.01; ***, *P* < 0.001; n.s. not significant.

We established a footpad implantation nude mouse model to evaluate the in vivo role of BACH1 in lymph node metastasis in athymic nude mice (Fig. 4A). The results demonstrated that ectopic BACH1 expression increased the size, weight and positive percentage (100% vs. 25%) of metastatic lymph nodes, whereas stable BACH1 knockdown exerted the opposite effects (Fig. 4B–D). Subsequently, we injected control and KYSE150-BACH1-OE cells via the tail vein to explore the effect of BACH1 on hematogenous metastasis and found that the lung metastatic efficiency of BACH1-OE cells was dramatically reduced (Fig. 4E, F).

To verify the effect of ferroptosis on in vivo tumor growth and metastasis, we first administered 30 mg/kg RSL3 or 10 mg/kg liproxstatin-1 (Liprox-1, a ferroptosis inhibitor) to the subcutaneous xenograft tumor model mice by intraperitoneal injection once every two days for 20 days. Liprox-1 slightly promoted the tumor growth, whereas, as expected, RSL3 had no inhibitory effect (Fig. 4G). In addition, we pretreated both control and KYSE150-BACH1-OE cells with 1 μM RSL3 for 6 h, and the subcutaneous tumorigenesis experiment showed that the in vivo proliferation and the tumor weight of BACH1-OE cells were dramatically attenuated (Fig. 4H). Next, using the footpad implantation nude mouse model, we found that compared with the control (DMSO), intraperitoneal RSL3 injection significantly promoted metastasis to the popliteal lymph nodes (16.7% vs. 83.3%), while the use of Liprox-1 had no significant effect (Fig. 4I, J). Taken together, the results indicate that BACH1 facilitates lymph node metastasis but inhibits hematogenous metastasis of ESCC in a ferroptosis-dependent manner, while ferroptosis itself inhibits the growth of subcutaneous tumors while promoting lymph node metastasis.

### BACH1 inhibits monounsaturated fatty acid (MUFA) biosynthesis to induce ferroptosis

**Fig. 5:**
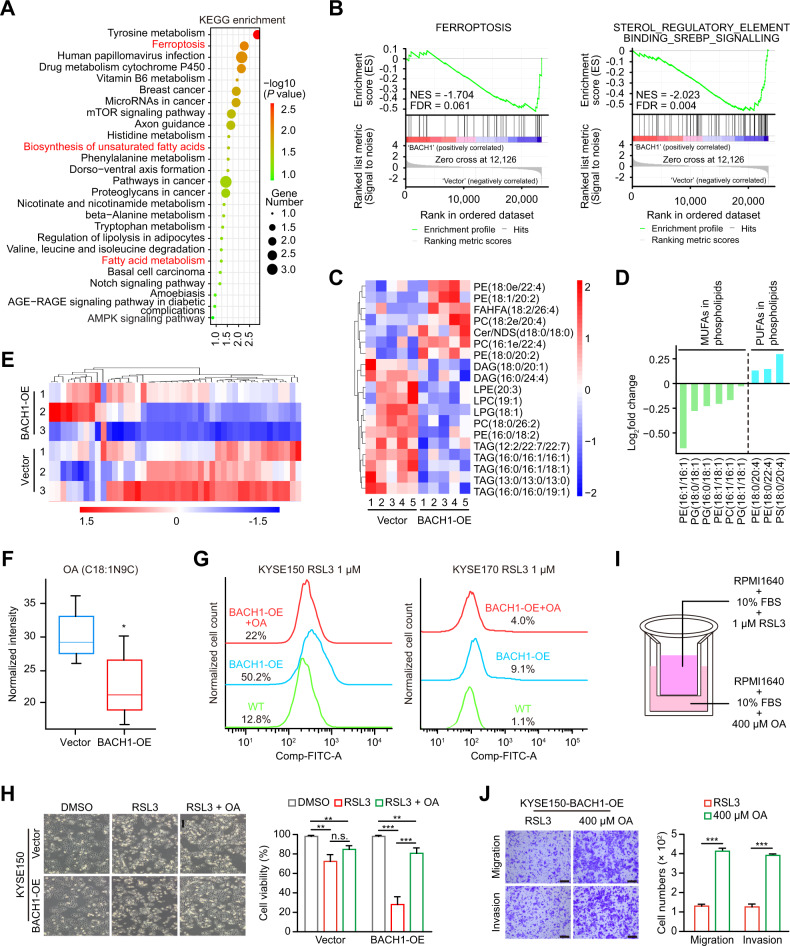
BACH1 inhibits intracellular MUFA synthesis. **A** Kyoto Encyclopedia of Genes and Genomes (KEGG) enrichment analysis of differentially expressed genes between KYSE150-BACH1-OE and KYSE150-vector cells based on RNA sequencing data. **B** Gene set enrichment analysis (GSEA) of BACH1-regulated genes with the normalized enrichment score (NES) and false discovery rate (FDR). BACH1 significantly negatively regulated ferroptosis (mainly as a suppressor) and sterol regulatory element-binding protein (SREBP) signaling. **C** Heatmap showing the differential lipids between KYSE150-BACH1-OE and KYSE150-vector cells based on nontargeted lipidomic analysis. **D** Changes in the phospholipid compositions in KYSE150-BACH1-OE cells showed that MUFAs decreased but PUFAs increased. **E** Heatmap showing the differential fatty acids between KYSE150-BACH1-OE and KYSE150-vector cells based on targeted lipidomic analysis. **F** Oleic acid (OA, C18:1N9C) levels were decreased in KYSE150-BACH1-OE cells. **G** Flow cytometric analysis after C11-BODIPY^591/581^ staining in KYSE150 and KYSE170 cells with transient ectopic expression of BACH1 followed by exposure to 1 μM RSL3 in the absence or presence of 400 μM OA. Compared with that in the WT group, lipid peroxidation was increased in the BACH1-OE groups, whereas OA attenuated the level of lipid peroxidation in the BACH1-OE + OA group. **H** Phase-contrast microscopic images and cell viability measurements in KSYE150-vector and KYSE150-BACH1-OE cells in the absence or presence of 1 μM RSL3 and 400 μM OA for 6 h. **I-J** Chemotaxis assay with OA for KYSE150-BACH1-OE cells exposed to 1 μM RSL3 based on the modified Transwell assays that are schematically represented in (**I**). The OA in the lower chamber was used as a chemoattractant to dramatically strengthen the migration and invasion capabilities of KYSE150-BACH1-OE cells. *, *P* < 0.05; **, *P* < 0.01; ***, *P* < 0.001; n.s. not significant.

To assess the mechanisms underlying BACH1-induced ferroptosis and lymph node metastasis, we performed RNA sequencing analysis on control and KYSE150-BACH1-OE cells. Kyoto Encyclopedia of Genes and Genomes enrichment analysis revealed that differentially expressed genes were enriched in fatty acid metabolism-related pathways in addition to ferroptosis (Fig. 5A). Gene set enrichment analysis also showed that BACH1 overexpression negatively regulated ferroptosis and sterol regulatory element binding protein (SREBP) signaling (Fig. 5B).

Fatty acid synthesis and metabolism pathways have been reported to be related to ferroptosis [12, 13]; thus, we performed lipidomic analysis on KYSE150-BACH1-OE and control cells. Heatmap analysis showed that the intracellular lipid composition significantly changed after BACH1 overexpression (Fig. 5C). Specifically, forced BACH1 expression caused a significant decrease in the production of monounsaturated fatty acid (MUFA)-containing phospholipids, while the production of the proferroptosis phospholipids phosphatidylethanolamine (PE)(18:0/20:4) and PE(18:0/22:4) increased to varying degrees (Fig. 5D), suggesting the dual-faceted impact of BACH1 on ferroptosis-related lipids in ESCC cells. Furthermore, targeted lipidomics was performed to quantitate the changes in fatty acids, and the results indicated significant decreases in medium- and long-chain fatty acids after BACH1 ectopic expression (Fig. 5E). Notably, OA was significantly reduced in BACH1-OE cells (Fig. 5F). OA is a MUFA that inhibits ferroptosis by reducing the amount or density of PUFAs in membranes [24]. Thus, BACH1 suppresses the synthesis of MUFAs, especially OA, to make ESCC cells more susceptible to ferroptosis.

### OA acts as a chemotactic agent in cells ectopically expressing BACH1 under ferroptotic pressure

Subsequently, we conducted recovery experiments on KYSE150 and KYSE170 cells. The addition of 400 μM OA mostly reversed the lipid reactive oxygen species (ROS) accumulation caused by BACH1-OE (Fig. 5G). Cell viability increased significantly in the presence of RSL3 after OA pretreatment (Fig. 5H). Additionally, we modified the traditional Transwell assay to investigate the chemotactic role of OA in the lower chamber and added 1 μM RSL3 to the upper chamber to induce ferroptosis (Fig. 5I). Surprisingly, OA obviously attracted BACH1-OE cells to promote cell migration and invasion compared with the control (Fig. 5J). Therefore, it seems that OA depletion is the main effector of BACH1-induced ferroptosis and that exogenous OA attractants protect sensitive cells from ferroptosis.

### BACH1 inhibits OA synthesis by negatively regulating SCD1

**Fig. 6:**
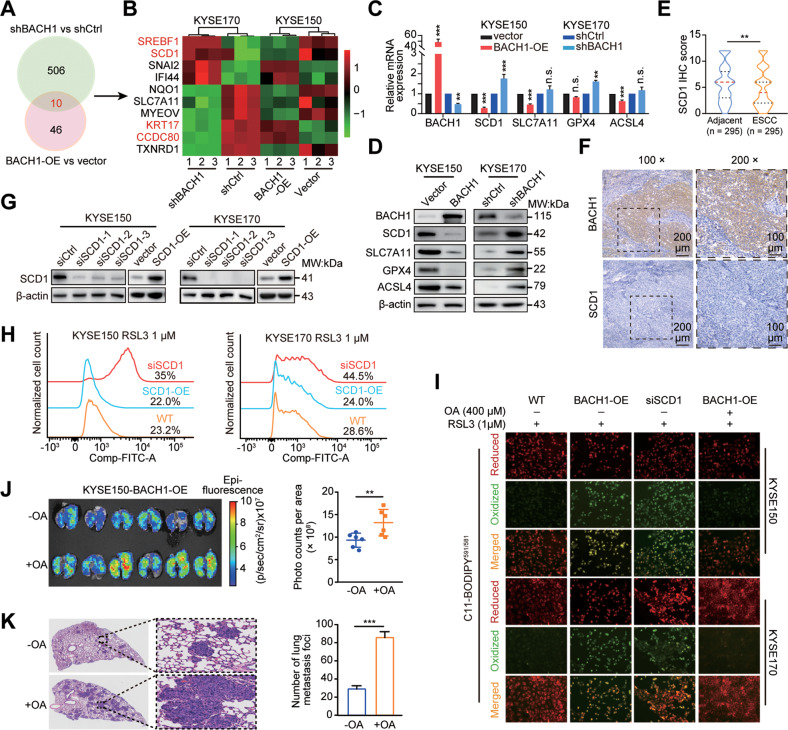
BACH1 inhibits OA synthesis by negatively regulating SCD1. **A** Venn diagram showing overlapping 10 differentially expressed genes between two comparisons, KYSE170-shBACH1 vs. shCtrl (516 genes) and KYSE150-BACH1-OE vs. vector (56 genes), based on RNA sequencing. **B** Heatmap showing the expression of 10 common genes in the three biological replicates of the indicated RNA sequencing data. **C, D** RT–qPCR (**C**) and western blot (**D**) assays validated the mRNA and protein expression of SCD1, SLC7A11, GPX4 and ACSL4 after overexpression or knockdown of BACH1 in KYSE150 and KYSE170 cells. **E** Comparison of the immunohistochemical (IHC) scores of SCD1 in 295 ESCC and paired adjacent nontumor tissues by the Wilcoxon signed rank test. **F** Representative images of BACH1 and SCD1 expression levels in serial sections are shown. 100× scale bar, 200 μm; 200× scale bar, 100 μm. **G** The efficiency of SCD1 knockdown (siSCD1-1, siSCD1-2 and siSCD1-3) or overexpression (SCD1-OE) in KYSE150 and KYSE170 cells was determined by western blotting. **H** Flow cytometric analysis after C11-BODIPY^591/581^ staining indicating the lipid peroxidation levels in KYSE150 and KYSE170 cells that were transiently transfected with SCD1 overexpression plasmid or siRNA oligos followed by treatment with 1 μM RSL3. **I** Lipid peroxidation was stained and visualized with the fluorescent probe C11-BODIPY^591/581^. KYSE150 and KYSE170 cells were transiently transfected with the BACH1 overexpression plasmid or siRNA oligos of SCD1 and then incubated with 1 μM RSL3 alone or RSL3 combined with 400 μM OA. Red and green fluorescence signals show normal lipids (reduced C11-BODIPY) and peroxidative lipids (oxidized C11-BODIPY), respectively. **J** Bioluminescence imaging (left panel) and quantification of photon flux (right panel) of lung metastatic tissues from NCG mice (n = 6 mice per group) with intravenous injection of KYSE150-BACH1-OE cells in the absence or presence of 400 μM OA. OA addition markedly promoted the pulmonary metastasis of ESCC cells. **K** Representative hematoxylin and eosin (H&E) staining and quantitative analysis of lung metastatic foci of dissected lungs from the NCG mice in panel (**J**), showing that OA addition (+OA) generated more and larger metastases than the control (-OA).

To investigate in depth the mechanism of OA synthesis inhibition by BACH1, we reanalyzed the RNA sequencing data. Compared with their respective controls, stable knockdown and forced expression of BACH1 yielded 516 and 56 differentially expressed genes, among which 10 genes were shared (Fig. 6A). Clustering analysis revealed that only four genes, including SREBF1, SCD, KRT17 and CCDC80, showed an opposite trend in two comparisons (Fig. 6B). We selected SCD1 for further analysis because it is the rate-limiting enzyme that catalyzes the conversion of saturated fatty acids, primarily OA, to MUFAs. Subsequent western blot and qPCR assays confirmed that BACH1 negatively regulated SCD1 mRNA and protein levels (Fig. 6C, D). Additionally, we evaluated the expression of SLC7A11, GPX4 and ACSL4 after the perturbations of BACH1 expression. Consistent with the RNA sequencing data, the overexpression and knockdown of BACH1 did not exert the opposite effects on the mRNA levels of these genes (Fig. 6C). However, their protein expression levels showed similar trends with that of SCD1 (Fig. 6D). Thus, it seems that BACH1 did not transcriptionally regulate these three regulators of ferroptosis, whereas BACH1-induced ferroptosis was accompanied by the downregulation of the ferroptosis effectors SLC7A11 and GPX4.

Then, we evaluated the relationship between SCD1 and BACH1 expression in clinical specimens, and an immunohistochemical staining assay showed that SCD1 was significantly downregulated in ESCC tissues (Fig. 6E, *n* = 295). Additionally, the protein level of BACH1 was negatively associated with that of SCD1 (Fig. 6F; *P* = 0.0018, chi-square test).

We knocked down or ectopically expressed SCD1 in KYSE150 and KYSE170 cells (Fig. 6G). SCD1 knockdown significantly increased lipid ROS accumulation in both cell lines, while ectopic expression slightly reduced intracellular lipid ROS levels (Fig. 6H). Cellular lipid ROS levels were also investigated using a fluorescence microscope under different treatment conditions. The tumor cells in both the BACH1-OE and siSCD1 groups showed more oxidized lipids (green fluorescence) than those in the control group, while OA addition significantly attenuated the oxidative phenotype (Fig. 6I). Furthermore, we investigated the in vivo roles of OA in pulmonary metastasis. We injected KYSE150-BACH1-OE cells with OA as a pretreatment, which significantly enhanced the size and number of pulmonary metastases compared with those in the group without OA pretreatment (Fig. 6J, K). These results collectively demonstrate that BACH1-induced ferroptosis is mediated by the inhibition of SCD1-catalysed OA synthesis.

### BACH1 transcriptional repression of SCD1

**Fig. 7:**
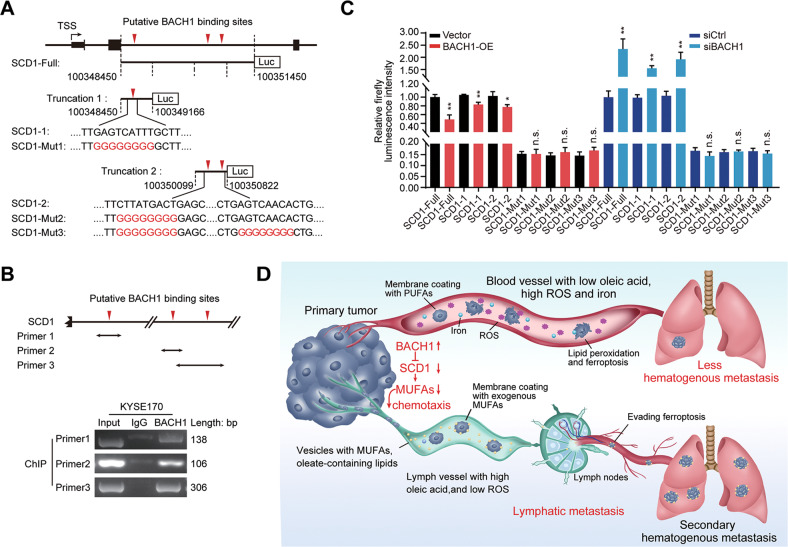
BACH1 transcriptionally inhibited SCD1 promoting ferroptosis and lymph node metastasis. **A** Schematics of luciferase reporters containing the full (3 kb; SCD1-Full), truncated (SCD1-1 and SCD1-2) and mutant (SCD1-Mut1, SCD1-Mut2, and SCD1-Mut3) sequences (3 kb) of three predicted BACH1 binding motifs in the intron 2 region of the SCD1 gene ~1.3–3.5 kb downstream of the transcription start sites (TSSs). The upside-down red triangles represent the predicted putative BACH1 binding motifs based on the JASPAR database. **B** ChIP-PCR assays indicated that BACH1 was bound to the intron 2 region of SCD1 through the three putative binding sites. The potential BACH1-DNA complex was immunoprecipitated from KYSE170 cells with an anti-BACH1 antibody, and the three binding regions in SCD1 were amplified by PCR. The PCR primers are labeled with double arrows in the schematics (top panel). **C** The firefly luciferase activity in KYSE170 cells cotransfected with the indicated plasmids or siRNAs with luciferase reporters, revealing that BACH1 binds to SCD1. **D** The basic mechanism by which BACH1 promotes ferroptosis and lymph node metastasis. In BACH1-overexpressing tumors, BACH1 reduces the content of OA in cell membranes by inhibiting SCD1, the rate-limiting enzyme for MUFA biosynthesis, resulting in tumor cell susceptibility to ferroptosis. Thus, hematogenous metastasis of tumor cells is inefficient because of the high levels of iron ions and oxidative stress in the blood. By contrast, since lymph fluid, which is rich in MUFAs, lymph fluid chemoattracts tumor cells with a low MUFA content to enter the lymphatic tracts. The cells are then wrapped by a protective layer of MUFAs, which enables them to escape ferroptosis. These processes increase the survival ability of tumor cells during subsequent dissemination through the blood, leading to multistation lymph node metastasis as well as secondary hematogenous metastasis. *, *P* < 0.05; **, *P* < 0.01; ***, *P* < 0.001.

To further confirm the transcriptional regulatory role of BACH1 in SCD1, we first predicted the putative BACH1 binding sites in SCD1 genes using the JASPAR database. Interestingly, no binding sites were predicted in the promoter region, while there were three putative sites in intron 2 (Fig. 7A). Then, we designed primers for the three putative binding sites and performed a ChIP-PCR assay. The results showed that BACH1 was physically bound to these sites in intron 2 of the SCD1 gene (Fig. 7B). Furthermore, luciferase reporter vectors were constructed with a 3-kb insertion (Chr10: 100348450–100351450) of the SCD1 intron 2 (SCD1-Full) and two truncations (~700 bp) containing the three putative binding sites (SCD1-1 and SCD1–2). Consistently, the relative luciferase activity was significantly increased in BACH1-knockdown cells and was suppressed in BACH1-OE cells compared with that in the respective controls (Fig. 7C). However, mutating the three putative binding sites of BACH1 led to the loss of its ability to inhibit the transcription of the SCD1 reporters (Fig. 7A). Therefore, these findings demonstrate that BACH1 transcriptionally represses SCD1 in ESCC cells through binding to its intron region.

## Discussion

ESCC and melanoma often form metastases in draining lymph nodes before developing distant metastases [1]. Metastasis to regional lymph nodes is a pivotal prognostic indicator for the outcomes of patients with these tumors [25]. Therefore, it is necessary to find some effective noninvasive biomarkers to assist with endoscopic ultrasound- and imaging-mediated discernment of cases with lymph node metastasis cases from early-stage patients.

It is generally accepted that the immune system recognizes cell abnormalities early in tumorigenesis and initiates a response, autoimmunity, to tumor-associated antigens to produce a unique autoantibody repertoire. These tumor-associated antigens are released into the tissue microenvironment and subsequently reach the lymph nodes via afferent lymphatic vessels, either in solution or after capture by antigen-presenting cells, effectively triggering a humoral immune response [21, 22, 26]. Several IgG and IgM autoantibodies have been used as early diagnostic tools for breast and lung cancers [27, 28]. In this study, we found a novel anti-BACH1 IgG autoantibody that was significantly correlated with early-stage lymph node metastasis. As our pan-cancer analysis revealed the overexpression of the BACH1 antigen in a variety of tumors with preferential lymphatic metastasis but not in most of tumors with preferential hematogenous metastasis, we propose that anti-BACH1 autoantibodies might be general markers for lymph node metastasis, even in other tumors with preferential lymphatic metastasis. However, this possibility needs to be validated in a larger-scale cohort.

Both BACH1 and its competitor NFE2L2 are members of the cap’n’ collar (CNC) and basic region leucine zipper family (CNC-bZip). Under normal physiological conditions, BACH1 forms a heterodimer with the small Maf oncoprotein and binds to Maf-recognition elements (MAREs) to inhibit target genes, including the gene encoding heme oxygenase-1 (HMOX1), a critical protector against oxidative stress [29]. Upon exposure to oxidative stress, BACH1 translocates into the cytoplasm to degrade [30], while NFE2L2 dissociates from its cytoplasmic inhibitor, Kelch-like ECH-associated protein 1 (KEAP1), and then enters the nucleus to bind to MAREs along with small Maf proteins, thus activating its target stress response genes [31].

Aside from oxidative stress, BACH1 has been shown to participate in the cell cycle, angiogenesis, metabolism regulation, and EMT, thus contributing to tumorigenesis and metastasis, although the results are controversial in different tumor types. BACH1 promotes the invasion and metastasis in prostate cancer, colorectal cancer, ovarian cancer, breast cancer, pancreatic cancer, non-small-cell lung cancer and ESCC [32, 33]. Additionally, BACH1 regulates the self-renewal and pluripotency of embryonic stem cells and maintains the characteristics of lung cancer stem cells [34–36].

By contrast, in acute myeloid leukemia cells, BACH1 promotes cell death by inhibiting HMOX1 expression [37]. In endothelial cells, BACH1 also impairs cell proliferation and promotes apoptosis through ROS generation [38]. In renal and pancreatic cancers, BACH1 has been reported to inhibit the proliferation and migration [39, 40]. Moreover, the roles of BACH1 in angiogenesis and lymphangiogenesis also showed controversial results in different studies [39, 41–43]. Collectively, this evidence suggests that the functions of BACH1 are cell or tissue context dependent; therefore, BACH1 expression shows diversity and complexity in different tumors.

Recently, BACH1 was found to induce ferroptosis in mouse embryonic fibroblasts exposed to erastin (an inhibitor of SLC7A11) by transcriptionally repressing genes that combat GSH and labile iron metabolism to accumulate iron-mediated damage, including GCLM, GCLC, SLC7A11, FTH1, FTL1, and SLC40A1 [44]. A previous study showed that BACH1 repressed the transcription of PPARG, a key regulator of adipogenesis, in immortalized mouse embryonic fibroblasts, to inhibit adipocyte differentiation [45]. PPARG-mediated ferroptosis in dendritic cells was found to limit antitumor immunity in mice [46].

Our study demonstrated the associations of both BACH1 protein antigen and BACH1 autoantibodies with lymph node metastasis in patients with ESCC. It appears that BACH1 overexpression is a common feature in tumors with preferential lymphatic metastasis based on the pan-cancer analysis of TCGA data. Additionally, a recent study has discovered a difference in the ferroptosis-related microenvironment between blood and lymph fluid [10]. These results led us to delve into the specific roles of BACH1, a ferroptosis inducer, in the selection of lymphatic or hematogenous metastatic routes.

Importantly, our results showed that BACH1-induced ferroptosis promoted lymph node metastasis but inhibited subcutaneous growth and hematogenous metastasis in vivo. This suggests that ferroptosis plays a two-faced role in primary lesion proliferation and tumor metastasis, consistent with the previous observation in melanoma that the rich OA content and low oxidative stress in the lymph result in an increased metastatic efficiency of tumor cells through evasion of ferroptosis [10]. These findings raise the questions of whether tumor cells have equal opportunity to enter the blood and lymph and whether the blood acts only as a filter by eliminating most metastatic cells via ferroptosis because of the high oxidative stress and iron content. Our subsequent functional studies revealed that BACH1 inhibited OA biosynthesis, and the low OA content in the cell membrane and the high OA content in lymph fluid formed a concentration gradient, leading to the chemoattraction of tumor cells and metastasis via lymphatic vessels. OA can be incorporated into the cell membrane to increase ferroptosis resistance [10, 24]. These results demonstrate that lymphatic metastasis is a proactive and smart choice for cells with high BACH1 expression because lymph is a sanctuary that enhances the survival ability and enables ferroptosis evasion (Fig. 7D).

To clarify the downstream targets of BACH1, we performed RNA sequencing analysis in two ESCC cell lines after a perturbation of BACH1 expression. Unfortunately, the previously reported ferroptosis-related targets of BACH1 (SLC40A1 and PPARG) were either not expressed in ESCC cells or showed no consistent alterations upon depletion or overexpression of BACH1 (Supplementary Fig. S1). Therefore, we suspected that BACH1-induced ferroptosis in ESCC cells was different from that in MEFs exposed to erastin. Furthermore, we found, for the first time, that BACH1 suppressed MUFA biosynthesis, especially OA biosynthesis. Our RNA sequencing and mechanistic studies revealed that BACH1 inhibited OA synthesis through transcriptional repression of SCD1. SCD1 is an endoplasmic reticulum-related enzyme involved in the de novo synthesis of fatty acids by catalyzing the desaturation of saturated fatty acids, such as stearic acid(18:0) and palmitic acid(16:0), to Δ9 MUFAs, such as OA(18:1) and palmitoleic acid(16:1) [47]. MUFAs can effectively inhibit ferroptosis by replacing PUFAs in lipid membranes and reducing the accumulation of lipid peroxidation products [24]. SCD1 has been found to protect tumor cells from ferroptotic cell death in ovarian, lung and gastric cancers [48–50]. Thus, BACH1 affects the sensitivity of cells to ferroptosis by regulating the fatty acid composition in cells, which results in cell preference to metastasize to lymph nodes first and may provide a new perspective for clinical treatment.

Notably, the BACH1-SCD1-OA axis is a novel signaling pathway in BACH1-induced ferroptosis. Our rescue assays revealed that either OA addition or SCD1 overexpression alleviated most of the alterations in lipid peroxidation, suggesting that there are other molecules involved in BACH1-induced ferroptosis, e.g., the known targets of BACH1 mentioned above.

Currently, the influence of ferroptosis on tumor metastasis remains unclear. The present and previous studies have confirmed that ferroptosis directly facilitates lymphatic metastasis but inhibits hematogenous metastasis [10]. BACH1 is the critical inducer in chemotactic tumor cells actively seeking refuge in the lymph. OA wraps around tumor cells, forming a protective layer to increase the secondary distant metastasis ability. Thus, it appears that the hypersensitivity to ferroptosis drives tumor cells to preferentially select the routes of lymphatic metastasis. Disrupting ferroptosis may help prevent lymph node metastasis and secondary systemic dissemination in clinical therapy. Heme can promote BACH1 degradation, and therefore, inhibiting lymph node metastasis in ESCC by increasing heme levels may be a promising therapeutic strategy. Additionally, although SCD1 acts as a main negative effector of BACH1-induced ferroptosis, it is a poor target because high SCD1 expression also promotes tumor cell proliferation [51]. Notably, because ferroptosis also inhibits primary tumor growth and hematogenous metastasis, the design of clinical trials with ferroptosis-associated agents requires a great caution. It is very challenging to determine the precise targets and appropriate timing of dosing.

In conclusion, we discovered that there were natural differences in ferroptosis sensitivity in solid tumors and that hypersensitive tumor cells were prone to preferentially select the routes of lymphatic metastasis. We also found that serum anti-BACH1 autoantibodies are promising biomarkers for detecting early lymph node metastasis. BACH1-induced ferroptosis promotes lymphatic but suppresses hematogenous metastasis via a novel BACH1-SCD1 axis in OA-mediated chemotaxis of tumor cells. These findings highlight the role of BACH1 in metastatic route selection, clarify the adverse effects of ferroptosis by promoting tumor metastasis, and provide a reference for the clinical application of ferroptosis-associated agents.

## Supplementary information

Supplementary Information

Supplementary tables

Original Data File

Checklist

## Data Availability

The datasets generated and/or analyzed during the current study are available from the corresponding author upon reasonable request.
