# Supplementary material for: BACH1-induced ferroptosis drives lymphatic metastasis by repressing the biosynthesis of monounsaturated fatty acids
Source: Cell Death Dis. 2023 Jan 20;14(1):48. doi: 10.1038/s41419-023-05571-z (PMC9860034; doi:10.1038/s41419-023-05571-z)
Supplement: Supplementary file 1 — Supplementary Information [file 41419_2023_5571_MOESM1_ESM.pdf]

# BACH1-induced ferroptosis drives lymphatic metastasis by repressing the biosynthesis of monounsaturated fatty acids

Xiufeng Xie[#], Lusong Tian[#], Yan Zhao, Fang Liu, Shuyang Dai, Xinglu Gu, Yuxin Ye, Lanping Zhou, Xinmiao Liu, Yulin Sun, Xiaohang Zhao

State Key Laboratory of Molecular Oncology, National Cancer Center/National Clinical Research Center for Cancer/Cancer Hospital, Chinese Academy of Medical Sciences and Peking Union Medical College, Beijing 100021, P. R. China

**Supplementary Figure:**

**Supplementary Fig. S1.** RT–qPCR assays validated the mRNA expression of SLC7A11, GCLM, FTH1 and FTL genes after overexpression or knockdown of BACH1 in KYSE150 and KYSE170 cells.

**Supplementary Tables:**

**Supplementary Table S1** Clinical characteristics of study populations used in screening of HuProt Proteome arrays

**Supplementary Table S2** Clinical characteristics of study populations used in immunoassay

**Supplementary Table S3** Primers information

**Supplementary Table S4** Antibodies information

**Supplementary Table S5** The upregulated IgG autoantibodies detected in patients with T1-stage ESCC

**Supplementary Table S6** The differentially expressed IgG autoantibodies between T1-stage ESCC that with or without lymph node metastasis

**Supplementary Table S7** The 41 overlapped tumor-related IgG autoantibodies in T1-stage ESCC with LNM compared with those without LNM

**Supplementary Table S8** P Percentage of anti-BACH1 IgG autoantibody positive cases by immunoblotting

**Supplementary Table S9** Clinical significance of anti-BACH1 autoantibody detected in patients with ESCC using immunoassay

**Supplementary Table S10** Clinicopathological characteristics of BACH1 protein expression in 574 ESCC specimens

## Supplementary Figure:

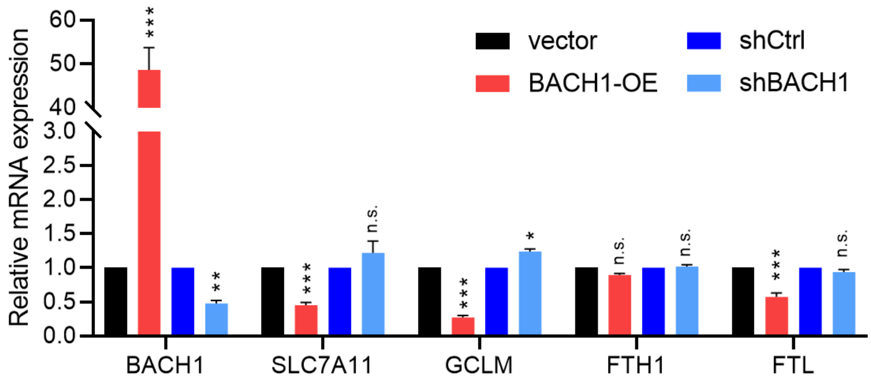


**Supplementary Figure S1.** RT–qPCR assays validated the mRNA expression of SLC7A11, GCLM, FTH1 and FTL genes after overexpression or knockdown of BACH1 in KYSE150 and KYSE170 cells. *, *P* < 0.05; **, *P* < 0.01; ***, *P* < 0.001; N.s., not significant.

## Supplementary Tables:

**Supplementary Table S1**. Clinical characteristics of study populations used in screening of HuProt Proteome arrays

| Patient Characteristics | ESCC w/ LNM (n = 12) | ESCC w/o LNM (n = 12) | Health control (n=12) | *P* value |
|---|---|---|---|---|
| Age in years | | | | |
| Mean ±SD | 59.5 ±8.93 | 60 ±9.93 | 59.41 ±8.60 | 0.98[a] |
| Range | 47-76 | 43-70 | 46-70 | |
| Gender | | | | |
| Male | 10 | 9 | 10 | 0.838[b] |
| Female | 2 | 3 | 2 | |
| TNM stage | | | | |
| T1 | 12 | 12 | | 1.00 [b] |
| T2 | 0 | 0 | | |
| T3 | 0 | 0 | | |
| T4 | 0 | 0 | | |
| Histological grade | | | | |
| High | 0 | 0 | | 0.881 [b] |
| Middle | 4 | 4 | | |
| Low | 4 | 5 | | |
| unknow | 4 | 3 | | |

a: *P* values were calculated by one-way ANONA

b: *P* values were calculated by *Chi* square test

**Supplementary Table S2**. Clinical characteristics of study populations used in immunoassay

| Patient Characteristics | ESCC w/ LNM (n = 78) | ESCC w/o LNM (n = 50) | Health control (n = 122) | *P* value |
|---|---|---|---|---|
| Age in years | | | | |
| Mean ±SD | 60.58 ±9.45 | 59.58 ±8.35 | 62.3 ±7.79 | 0.122[a] |
| Range | 37-79 | 41-77 | 43-77 | |
| Gender | | | | |
| Male | 69 | 37 | 102 | 0.104[b] |
| Female | 9 | 13 | 20 | |
| TNM stage | | | | |
| T1 | 6 | 10 | | 0.017[b] |
| T2 | 10 | 13 | | |
| T3 | 45 | 22 | | |
| T4 | 17 | 5 | | |
| Histological grade | | | | |
| High | 6 | 9 | | 0.164[b] |
| Middle | 32 | 22 | | |
| Low | 15 | 10 | | |
| unknow | 25 | 9 | | |

a: *P* values were calculated by one-way ANONA

b: *P* values were calculated by *Chi* square test

**Supplementary Table S3**. Primers information

| Primer sequences and oligonucleotides |
|---|
| BACH1 qPCR primer, Forward: AGACGACTCTGAGACGGACA |
| BACH1 qPCR primer, Reverse: CGCTGTGCAGCAATTCTGTT |
| SREBF1 qPCR primer, Forward: ACAGTGACTTCCCTGGCCTAT |
| SREBF1 qPCR primer, Reverse: GCATGGACGGGTACATCTTCAA |
| SCD1 qPCR primer, Forward: TTCCTACCTGCAAGTTCTACACC |
| SCD1 qPCR primer, Reverse: CCGAGCTTTGTAAGAGCGGT |
| ATP5J qPCR primer, Forward: GTTCTCCTCTGTCATTCGGTCA |
| ATP5J qPCR primer, Reverse: TCACTCCAGATGTCTGTCGCTTAGAT |
| ATP5G qPCR primer, Forward: CCAGAGTTGCATACAGACCAAT |
| ATP5G qPCR primer, Reverse: CCCATTAAATACCGTAGAGCCCT |
| UCP3 qPCR primer, Forward: TGTTTTGCTGACCTCGTTACC |
| UCP3 qPCR primer, Reverse: GACGGAGTCATAGAGGCCGAT |
| TIMM8B qPCR primer, Forward: TCACTTCATGGAGTTATGTTGGG |
| TIMM8B qPCR primer, Reverse: AGACAATTTTCAGTGCGAGAGTC |
| SLC25A22 qPCR primer, Forward: GCCAGCCAAGCTCATCAATG |
| SLC25A22 qPCR primer, Reverse: GAGGCAGTCGGACATGCTC |
| PDHX qPCR primer, Forward: TTGGGAGGTTCCGACCTGT |
| PDHX qPCR primer, Reverse: CAACCACTCGACTGTCACTTG |
| OCT1 qPCR primer, Forward: GTGTGTAGACCCCCTGGCTA |
| OCT1 qPCR primer, Reverse: GTGTAGCCAGCCATCCAGTT |
| NDUFB6 qPCR primer, Forward: CCACAGAAGATGGGGCCTATG |
| NDUFB6 qPCR primer, Reverse: TCCAGACAGGTACAAGTACATGA |
| SLC7A11 qPCR primer, Forward: TCTCCAAAGGAGGTTACCTGC |
| SLC7A11 qPCR primer, Reverse: AGACTCCCCTCAGTAAAGTGAC |
| GPX4 qPCR primer, Forward: GAGGCAAGACCGAAGTAAACTAC |
| GPX4 qPCR primer, Reverse: CCGAACTGGTTACACGGGAA |
| ChIP-PCR primer 1, Forward: CCTTGTGCTTGTGGGCTTTG |
| ChIP-PCR primer 1, Reverse: TCCCAGGTAGAGGGGTTTGA |
| ChIP-PCR primer 2, Forward: TAGCAACTGGTCGTGATTCCAG |
| ChIP-PCR primer 2, Reverse: GAGGCATTCTCCTAAGGGCT |
| ChIP-PCR primer 3, Forward: AATGCCTCTGGCAACACTGA |
| ChIP-PCR primer 3, Reverse: AGCAAGGGAGCTTCTAGGGT |
| ACTB qPCR primer, Forward: CTCACCATGGATGATGATATCGC |
| ACTB qPCR primer, Reverse: TAGGAATCCTTCTGACCCATGC |
| siRNA negative control sense (5'-3'):UUCUCCGAAGGUGUCACGUTT |
| siRNA negative control antisense (5'-3'):ACGUGACACGUUCGGAGAATT |

| |
|---|
| BACH1 silencing siRNA -1 sense (5'-3'): GGACCAGAGGGAUCUAGAATT |
| BACH1 silencing siRNA -1 antisense (5'-3'): UUCUAGAUCCCUCUGGUCCTT |
| SCD1 silencing siRNA -1 sense (5'-3'): GGAGAAACAUCAUCCUUAUTT |
| SCD1 silencing siRNA -1 antisense (5'-3'): AUAAGGAUGAUGUUUCUCCTT |
| SCD1 silencing siRNA -2 sense (5'-3'): GCUUGCUGAUGAUGUGCUUTT |
| SCD1 silencing siRNA -2 antisense (5'-3'): AAGCACAUCAUCAGCAAGCTT |
| SCD1 silencing siRNA -3 sense (5'-3'): GCGAUAUGCUGUGGUGCUUTT |
| SCD1 silencing siRNA -3 antisense (5'-3'): AAGCACCACAGCAUAUCGCTT |

**Supplementary Table S4**. Antibodies information

| Antibodies | Source | Catalog number/ identifier |
|---|---|---|
| BACH1 for WB, IHC & IF | Abclonal | A5393/ RRID: AB_2766202 |
| BACH1 for IP | R&D Systems | AF5776/ RRID: AB_2061974 |
| SLC7A11 | Proteintech | 26864-1-AP, RRID: AB_2880661 |
| GPX4 | Abcam | ab41787, RRID: AB_941790 |
| ACSL4 | Abcam | ab205197 |
| AMPK | CST | 5831/ RRID: AB_ 10622186 |
| p-AMPK | CST | 2535/ RRID: AB_331250 |
| SREBF1 | Proteintech | 14088-1-AP/ RRID: AB_2255217 |
| SCD1 | Abcam | ab236868 |
| β-Actin | Sigma-Aldrich, St. Louis, MO | A5316, RRID: AB_476743 |
| Flag | MBL, Sakae Naka-ku Nagoya, Japan | M185-3L/ RRID:AB_11123930 |
| HA | CST, Danvers, MA | 3724s/ RRID:AB_1549585 |
| HRP-conjugated mouse secondary antibody | CST | 58802/ RRID:AB_2799549 |
| HRP-conjugated rabbit secondary antibody | CST | 93702/ RRID:AB_2800208 |

WB, western blot analysis; IHC, immunohistochemistry; IF, immunofluorescence taining; IP, immunoprecipitation; MBL, Medical & Biological Laboratories; CST, Cell Signaling Technology; RRID, Research Resource Identifier (https://scicrunch.org/resources)

**Supplementary Table S8**. Percentage of anti-BACH1 IgG autoantibody positive cases by immunoblotting

| | Negative | Positive | *P* value | AUC | Sensitivity% | Specificity% |
|---|---|---|---|---|---|---|
| Healthy controls | 119 (97.5%) | 3 (2.5%) | <0.001 | 0.767 | 32.8 | 95 |
| ESCC | 98 (76.6%) | 30 (23.4%) | | | | |
| Healthy controls | 119 (97.5%) | 3 (2.5%) | 0.0095 | 0.695 | 20.5 | 95 |
| Early ESCC | 34 (87.2%) | 5 (12.8%) | | | | |
| ESCC w/o LNM | 44 (88%) | 6 (12%) | 0.0145 | 0.672 | 14.1 | 95 |
| ESCC w/ LNM | 54 (69.2%) | 24 (30.8%) | | | | |

LNM, lymph node metastasis.

The cut-off value was determined as the mean+3SDs of healthy controls.

*P* values were calculated by *Chi* square test.

**Supplementary Table S9**. Clinical significance of anti-BACH1 autoantibody detected in patients with ESCC using immunoassay

| | Negative, n (%) | Positive, n (%) | *P* value |
|---|---|---|---|
| Age | | | 0.880 |
| >=60 | 54 (76.1%) | 17 (23.9%) | |
| <60 | 44 (77.2%) | 13 (22.8%) | |
| Gender | | | 0.058 |
| Male | 78 (73.6%) | 28 (26.4%) | |
| Female | 20 (90.9%) | 2 (9.1%) | |
| Histological grade | | | 0.568 |
| High | 12 (80%) | 3 (20%) | |
| Middle | 44 (81.5%) | 10 (18.5%) | |
| Low | 17 (68%) | 8 (32%) | |
| Unknown | 25 (73.5%) | 9 (26.5%) | |
| Tumor location | | | 0.1848 |
| Upper | 7 (87.5%) | 1 (12.5%) | |
| Middle | 19 (63.3%) | 11 (36.7%) | |
| Lower | 29 (82.9%) | 6 (17.1%) | |
| Unknown | 43 (78.2%) | 12 (21.8%) | |
| Tumor size | | | 0.0924 |
| <5 cm | 41 (87.2%) | 6 (12.8%) | |
| >= 5cm | 37 (71.2%) | 15 (28.8%) | |
| Unknown | 20 (69%) | 9 (31%) | |
| T stages | | | 0.290 |
| T1 | 14 (87.5%) | 2 (12.5%) | |
| T2 | 20 (87%) | 3 (13%) | |
| T3 | 49 (73.1%) | 18 (26.9%) | |
| T4 | 15 (68.2%) | 7 (31.8%) | |
| N staging | | | 0.0145 |
| Negative | 44 (88%) | 6 (12%) | |
| Positive | 54 (69.2%) | 24 (30.8%) | |
| M staging | | | 0.99 |
| Yes | 7 (77.8%) | 2 (22.2%) | |
| No | 91 (76.5%) | 28 (23.5%) | |
| AJCC | | | 0.0012 |
| Stage 0-II | 45 (91.8%) | 4 (8.2%) | |
| Stage III-IV | 53 (67.1%) | 26 (32.9%) | |

*P* values were calculated by *Chi* square test.

**Supplementary Table S10**. Clinicopathological characteristics of BACH1 protein expression in 574 ESCC specimens

| | BACH1 expression | | |
|---|---|---|---|
| | Negative, n (%) | Positive, n (%) | *P* value |
| Gender | | | 0.14119 |
| Male | 360 (86.33%) | 57 (13.67%) | |
| Female | 140 (90.91%) | 14 (9.09%) | |
| Age | | | 0.624601 |
| <65 | 269 (88.20%) | 36 (11.80%) | |
| >= 65 | 231 (86.84%) | 35 (13.16%) | |
| Histological grade | | | 0.000079 |
| I | 35 (25.74%) | 101 (74.26%) | |
| I-II+II | 175 (55.03%) | 143 (44.97%) | |
| II-III+III | 82 (71.93%) | 32 (28.07%) | |
| Tumor size | | | 0.65449 |
| <5cm | 120 (78.95%) | 32 (21.05%) | |
| >= 5cm | 103 (81.10%) | 24 (18.90%) | |
| T staging | | | 0.901433 |
| T1 | 27 (90.00%) | 3 (10.00%) | |
| T2 | 95 (86.36%) | 15 (13.64%) | |
| T3 | 349 (87.47%) | 50 (12.53%) | |
| T4 | 13 (86.67%) | 2 (13.33%) | |
| N staging | | | 0.03646 |
| N0-2 | 481 (88.10%) | 65 (11.90%) | |
| N3 | 15 (71.43%) | 6 (28.57%) | |
| M staging | | | 1 |
| M0 | 493 (87.57%) | 70 (12.43%) | |
| M1 | 7 (87.50%) | 1 (12.50%) | |
| TNM staging | | | 0.251737 |
| I | 54 (93.10%) | 4 (6.90%) | |
| II | 208 (86.67%) | 32 (13.33%) | |
| III | 196 (87.89%) | 27 (12.11%) | |
| IV | 26 (78.79%) | 7 (21.21%) | |
