# Supplementary material for: BACH1-induced ferroptosis drives lymphatic metastasis by repressing the biosynthesis of monounsaturated fatty acids
Source: Cell Death Dis. 2023 Jan 20;14(1):48. doi: 10.1038/s41419-023-05571-z (PMC9860034; doi:10.1038/s41419-023-05571-z)
Supplement: Supplementary file 2 — Supplementary tables [file 41419_2023_5571_MOESM2_ESM.pdf]

Supplementary Table S5. The upregulated IgG autoantibodies detected in patients with T1-stage ESCC

Gene Symbol	ID	HC1	HC2	HC3	HC4	w/o LNM1	w/o LNM2	w/o LNM3	w/o LNM4	w/ LNM1	w/ LNM2	w/ LNM3	w/ LNM4	FC (ESCC vs. HC)
TP53	NM_000546.2	2.021	1.940	2.364	2.488	18.935	11.426	6.076	3.785	3.341	2.191	44.063	2.810	5.255
MARCKSL1	BC066915.1	2.107	2.113	1.738	2.414	3.501	8.852	3.186	4.864	2.159	1.959	2.045	2.276	1.723
MED22	NM_133640.3	3.620	2.536	2.410	2.926	6.189	9.994	4.341	5.227	17.923	5.751	7.606	17.238	3.232
CDCA7L	NM_018719.2	3.249	2.522	2.109	2.866	3.559	4.708	3.911	4.429	4.713	7.508	2.790	2.983	1.610
ERBB4	NM_001042599.1	3.687	2.331	2.667	3.428	6.751	6.294	29.899	3.769	3.253	12.651	3.555	3.263	2.866
FAM131C	NM_182623.1	3.470	2.939	2.601	3.892	4.451	16.001	9.146	10.507	2.665	9.139	2.686	7.747	2.416
RAP1GDS1	BC098334.1	3.787	2.186	1.950	3.652	12.782	3.899	5.109	4.487	4.395	9.271	2.285	2.671	1.939
PNMA2	BC036489.1	3.289	2.806	2.525	3.668	3.799	4.311	10.668	3.954	4.728	6.842	22.827	4.372	2.503
SLC25A26	BC012852.1	3.450	3.253	2.206	3.582	5.240	10.479	3.685	5.496	4.897	6.221	3.262	3.080	1.696
LGALS8	NM_201543.1	4.602	6.611	3.461	5.637	7.843	16.278	12.722	10.923	10.645	9.897	15.162	3.912	2.151
SPATC1	NM_198572.1	2.090	2.079	1.805	2.156	4.264	3.010	4.166	2.860	2.237	3.061	5.895	2.218	1.704
KLHL3	BC034035.1	2.852	2.444	2.315	2.449	5.670	3.401	2.896	4.285	2.970	10.512	4.447	2.061	1.801
SBSN	NM_198538.1	2.445	2.518	2.058	2.228	2.902	6.642	2.601	3.340	3.461	4.268	2.320	2.226	1.501
DEFB126	NM_030931.2	2.371	3.317	2.101	5.021	18.619	14.375	3.643	9.652	6.034	7.946	12.052	6.568	3.079
EMCN	BC017781.1	2.933	3.692	2.212	7.990	14.774	6.802	18.901	9.702	33.074	9.889	4.959	11.743	3.264
GORASP1	NM_031899.2	2.437	2.906	3.496	6.294	17.425	7.444	7.271	5.509	8.343	10.214	2.925	9.209	2.258
ITGB8	NM_002214.2	2.851	3.956	1.958	4.509	7.755	7.231	3.454	7.831	3.622	5.045	4.792	2.821	1.603
PODNL1	NM_024825.2	3.093	2.865	2.319	4.371	4.289	4.445	5.664	4.703	7.649	17.810	6.058	3.552	2.142
NRG3	NM_001010848.3	5.420	4.952	2.892	4.431	20.419	6.008	3.868	11.596	3.415	8.899	8.476	2.762	1.849
STAT5A	NM_003152.2	4.202	3.102	2.177	3.054	6.538	11.821	5.341	3.808	6.857	4.946	2.781	2.529	1.780
PROL1	NM_021225.4	6.479	3.162	2.208	4.335	10.474	8.234	3.951	8.716	3.911	6.482	5.514	4.074	1.587
RXRA	BC007925.1	3.700	3.496	2.123	3.239	4.081	10.437	3.273	4.741	5.141	6.679	3.092	2.941	1.608
SIGLEC8	BC053319.1	3.135	3.156	2.494	3.601	8.072	3.453	3.605	6.634	3.410	6.250	6.243	3.838	1.676
PRM1	NM_002761.1	4.702	4.184	2.408	4.274	5.764	11.117	3.512	6.308	5.917	7.160	3.577	3.428	1.503
ST6GAL2	BC008680.1	4.069	3.333	2.563	4.304	8.516	4.448	4.043	5.680	4.061	6.680	5.786	4.526	1.533
PTGES3L-AARSD1	NM_025267.2	3.210	3.363	3.163	4.656	4.961	22.644	4.535	16.902	4.584	3.457	4.534	2.234	2.218
PALM2	NM_001037293.1	3.877	14.004	13.889	7.242	29.660	35.582	3.811	45.031	41.168	8.780	6.586	5.157	2.253
PAGE5	NM_001013435.1	2.512	2.220	1.746	4.326	3.845	4.611	3.433	9.463	12.826	11.111	1.954	79.497	5.866
IL21R	NM_021798.2	3.252	2.821	3.158	5.262	13.125	3.755	4.051	8.158	3.793	7.691	8.575	3.805	1.827

Gene Symbol	ID	HC1	HC2	HC3	HC4	w/o LNM1	w/o LNM2	w/o LNM3	w/o LNM4	w/ LNM1	w/ LNM2	w/ LNM3	w/ LNM4	FC (ESCC vs. HC)
ZG16B	ENST00000382280	4.403	7.976	5.505	5.385	6.705	16.451	7.452	9.108	6.767	12.480	8.403	9.572	1.653
RPSA	BC066941.1	2.879	2.183	2.158	2.249	2.026	3.722	5.462	11.275	4.252	2.985	6.109	6.261	2.223
TEAD3	BC027877.1	2.714	2.618	2.091	2.764	4.358	4.504	2.244	9.708	4.607	3.856	2.260	3.141	1.702
Sp3	BC079874	2.167	1.875	2.584	2.558	2.237	5.943	3.830	4.220	2.675	2.143	2.593	10.596	1.864
KIAA0317	BC032944.2	2.617	2.830	2.222	2.891	6.224	4.504	2.681	3.265	4.175	12.334	2.462	2.951	1.827
OFD1	BC099658.1	3.656	1.937	2.226	2.158	11.908	2.720	9.437	5.305	5.507	10.248	2.435	2.708	2.519
THUMPD1	NM_017736.3	2.773	3.143	1.854	2.944	3.901	18.712	2.675	3.677	473.087	2.088	150.213	50.600	32.899
LRRC6	BC027589.1	2.864	2.595	1.928	3.863	10.807	6.857	2.807	5.642	5.563	6.893	2.350	2.963	1.950
CTAG1A	NM_139250	2.363	3.105	1.965	2.233	4.134	3.829	9.575	2.578	3.074	2.940	2.293	2.528	1.601
KCNA3	NM_002232.3	3.236	2.387	2.393	2.588	2.351	4.738	5.481	8.816	4.288	3.288	7.150	4.034	1.893
PPP1R13L	BC064913.1	2.374	2.193	3.050	3.073	12.552	19.951	2.481	3.376	2.699	4.196	7.853	2.527	2.602
SNX1	NM_003099.3	3.572	2.069	2.593	4.510	2.054	8.848	21.533	12.537	4.678	3.603	2.770	5.323	2.407
MAGEB6	NM_173523.2	3.028	2.669	2.272	3.115	3.312	2.988	11.986	3.225	3.773	3.110	2.838	3.420	1.563
CTRB2	NM_001025200.3	2.344	3.330	2.015	3.525	7.665	3.812	2.684	4.312	3.958	4.727	3.564	2.935	1.501
C2	BC029781.1	3.274	3.865	2.223	4.371	8.125	6.800	2.914	5.365	5.912	5.592	4.329	3.624	1.553
TMEM25	BC051841.1	6.959	3.126	5.776	4.692	2.341	42.395	15.286	11.438	4.216	3.252	3.023	33.097	2.799
C6orf106	NM_024294.2	1.502	1.746	1.579	1.335	7.198	19.021	1.801	1.744	11.275	8.813	1.743	1.780	4.331
SMEK1	BC072409.1	2.013	2.143	2.075	1.893	2.600	23.613	1.959	3.379	6.965	4.856	45.027	2.259	5.580
VPS35	BC010362.1	2.622	2.494	2.104	2.215	6.437	4.266	2.455	2.778	3.194	14.703	2.431	2.637	2.062
FUT6	BC061700.1	1.683	2.313	1.735	2.360	4.811	2.298	2.484	3.530	2.396	3.221	3.103	2.446	1.501
PIK3CA	NM_006218.2	2.247	2.301	1.918	3.180	2.879	5.543	2.616	6.250	2.714	3.415	2.297	4.301	1.556
PAGE2	NM_207339.1	4.772	1.953	1.754	3.290	3.333	5.065	6.629	9.190	17.335	3.414	2.725	46.111	3.985
ERMP1	BC031630.1	2.792	3.700	2.415	4.322	11.543	4.365	3.315	6.355	4.216	5.501	6.820	4.256	1.753
ZNF720	BC055408.1	4.862	5.120	2.366	3.891	5.790	13.004	4.048	5.207	7.098	7.843	3.649	3.189	1.534
ANKHD1	NM_017978.1	2.979	3.868	1.877	1.694	3.052	3.018	12.204	35.719	2.588	4.807	2.257	34.375	4.704
PICK1	NM_012407.3	3.474	2.553	2.260	4.914	4.709	6.955	3.162	19.712	3.326	3.545	27.301	5.075	2.795
ZNF641	BC018090.1	3.430	3.748	2.375	2.154	4.464	8.209	3.512	3.385	5.656	4.194	4.469	2.599	1.558
TCOF1	NM_001008657.1	3.544	3.370	2.054	5.391	4.931	71.191	9.302	3.200	34.229	24.672	20.500	3.845	5.985
GBP5	NM_052942.2	5.527	4.678	2.489	4.623	3.861	28.221	3.009	42.038	5.435	8.200	13.848	2.672	3.098
ST3GAL3	NM_006279.2	4.763	3.811	2.646	6.063	15.148	5.865	4.508	9.486	5.667	5.814	8.642	4.475	1.724
SPDL1	NM_017785.2	3.493	3.101	3.901	2.761	3.089	8.244	10.735	3.411	7.247	17.435	31.742	5.579	3.299

Gene Symbol	ID	HC1	HC2	HC3	HC4	w/o LNM1	w/o LNM2	w/o LNM3	w/o LNM4	w/ LNM1	w/ LNM2	w/ LNM3	w/ LNM4	FC (ESCC vs. HC)
LAMP1	NM_005561.3	5.649	6.432	2.409	9.832	14.443	7.300	6.416	16.127	15.690	6.624	8.533	6.946	1.687
CLDN8	NM_199328.1	4.075	3.240	2.394	4.882	10.362	4.025	4.243	6.274	4.792	3.963	7.239	4.254	1.547
P2RX4	BC033826.1	4.382	7.329	2.828	12.585	14.126	10.705	4.960	15.305	12.252	9.026	10.196	9.181	1.581
GIF	NM_005142.2	3.802	3.909	2.639	4.902	6.289	4.795	4.014	7.853	3.866	6.957	6.088	27.069	2.194
TPM2	NM_003289.3	5.233	5.397	4.170	9.241	5.369	4.870	15.191	14.909	44.130	6.207	39.102	7.845	2.862
LAT	NM_001014989.1	3.978	3.045	2.581	11.386	81.343	5.501	5.337	9.297	3.879	13.632	6.021	6.146	3.124
ZNF428	BC045799.1	4.886	9.844	3.578	4.432	5.887	6.027	5.931	89.333	5.118	10.237	7.025	7.183	3.007
FAM43A	NM_153690.4	2.335	2.258	2.034	3.170	3.444	2.229	3.867	18.768	3.577	11.170	11.461	2.196	2.894
DCDC2	Q9UHG0	4.382	2.498	2.047	2.267	5.682	4.923	2.483	13.302	5.376	3.060	5.488	2.394	1.908
KIR3DS1	NM_001083539.1	2.196	2.966	2.039	3.077	9.991	3.239	2.361	3.496	3.602	5.292	3.048	2.237	1.618
TNNC2	NM_003279.2	2.670	6.130	2.000	3.464	239.006	2.162	6.772	6.243	15.673	2.824	2.398	25.067	10.521
IFNLR1	NM_170743.2	3.063	2.254	2.473	3.527	12.958	2.615	3.540	4.663	2.781	2.721	5.612	4.517	1.741
PUF60	NM_014281.3	3.224	3.060	2.304	2.339	3.379	8.697	2.290	3.461	3.316	14.607	4.147	2.983	1.962
PAG1	NM_018440.3	2.078	1.933	2.243	5.571	30.320	1.951	5.166	13.961	2.644	4.421	10.764	11.434	3.411
ATF6	BC014969.1	2.298	3.903	2.614	2.624	26.634	16.387	2.167	3.648	6.683	6.242	2.648	2.070	2.906
TMSB10	NM_021103.3	3.263	1.935	2.296	2.102	1.838	3.137	11.521	6.610	4.562	3.158	17.189	2.007	2.607
MZB1	NM_016459.3	4.322	4.020	2.282	2.929	5.998	10.290	2.849	3.956	7.860	6.013	3.224	2.498	1.575
NSG1	NM_014392.3	2.908	5.300	2.964	4.996	9.164	9.450	2.974	4.410	3.723	14.917	12.721	3.260	1.875
TIFA	NM_052864.2	3.222	2.985	3.178	3.072	3.582	3.147	58.909	2.718	3.300	3.659	2.956	2.422	3.239
FOLH1	NM_001014986.1	3.104	3.352	2.330	4.579	9.593	4.311	2.976	6.255	5.244	5.671	6.071	4.540	1.671
FAM131B	BC045611.1	4.230	4.026	3.910	5.135	2.734	18.114	5.745	4.528	11.066	3.370	2.492	13.221	1.771
PTMA	NM_002823.2	2.334	1.903	2.042	1.922	1.542	1.648	129.886	4.638	3.628	3.081	4.423	2.693	9.239
RBM12	NM_006047.4	2.551	2.872	2.010	2.657	2.700	6.542	2.579	6.162	5.442	3.175	2.441	5.741	1.724
SCGB1D1	NM_006552.1	2.210	2.226	2.012	2.965	3.897	2.410	2.348	7.292	3.046	8.444	3.024	2.358	1.743
GCG	NM_002054.2	2.840	2.123	2.262	2.917	2.186	2.215	7.866	4.903	2.080	2.565	3.894	9.021	1.712
FAM153A	NM_173663.1	2.419	2.594	1.723	3.692	22.058	104.620	1.887	2.751	4.450	3.433	3.146	2.981	6.968
STX10	BC017237.2	2.713	2.933	2.420	3.012	2.941	19.395	2.366	3.158	11.019	4.347	8.081	2.369	2.423
CPLX2	NM_001008220.1	2.221	4.593	1.851	1.721	5.444	7.402	2.065	2.326	5.646	4.793	2.078	2.250	1.541
TRIM24	NM_015905.2	2.098	3.955	1.954	2.129	4.533	6.730	2.493	2.577	4.651	4.745	3.673	3.512	1.624
RAD23B	BC020973.2	2.458	2.901	2.366	6.002	2.640	7.305	69.280	2.806	7.316	9.120	11.904	37.760	5.396
BACH1	BC063307	2.889	3.030	8.966	2.515	7.873	2.935	4.564	4.592	13.341	38.809	16.844	10.877	2.869

Gene Symbol	ID	HC1	HC2	HC3	HC4	w/o LNM1	w/o LNM2	w/o LNM3	w/o LNM4	w/ LNM1	w/ LNM2	w/ LNM3	w/ LNM4	FC (ESCC vs. HC)
SH3GL2	NM_003026.1	2.809	3.192	3.167	2.130	3.075	2.787	26.636	2.699	6.121	7.791	6.331	3.324	2.601
ATXN3	BC033711.1	11.373	5.177	4.120	3.540	5.214	29.918	3.568	12.114	92.147	34.882	25.934	11.438	4.445
ZPR1	NM_003904.3	4.668	3.860	2.990	4.430	3.550	6.365	3.584	3.039	4.884	35.133	6.189	6.535	2.172
USH1C	BC016057.1	2.844	2.279	2.850	2.011	2.371	5.727	4.343	2.254	4.275	9.847	14.711	3.488	2.354
MAGEA2	BC063681.1	2.263	2.144	1.796	2.409	3.137	2.036	2.422	2.638	3.098	3.240	2.618	8.597	1.613
PI4K2B	BC051749.1	2.260	2.171	2.015	3.624	3.257	2.058	2.570	4.522	3.376	4.171	3.849	13.751	1.865
RNASET2	NM_003730.3	2.115	2.311	2.478	2.922	3.219	2.940	2.615	3.059	3.059	3.119	11.202	2.920	1.635
DPH2	NM_001384.4	2.666	2.691	2.149	2.925	4.321	2.493	4.355	2.589	4.201	4.776	2.900	7.426	1.585
ALPP	NM_001632.3	2.118	2.188	2.043	2.684	3.824	2.245	2.697	4.321	2.661	4.587	4.015	3.961	1.567
FAF1	NM_007051.2	7.225	2.693	2.458	5.838	2.871	7.518	6.437	20.149	11.238	3.502	62.420	26.805	3.869
IVNS1ABP	BC067739	4.184	4.611	3.231	3.678	2.725	6.602	3.440	3.577	19.697	22.579	6.850	3.939	2.210
SPA17	NM_017425.2	1.874	2.185	1.710	2.003	1.836	3.714	1.941	1.891	4.030	5.428	1.777	12.951	2.160
CRYL1	NM_015974.2	2.916	2.807	1.871	2.589	3.945	3.664	2.141	2.771	3.119	5.862	18.161	2.461	2.068
LAX1	BC069650.1	3.307	1.746	3.097	4.061	3.157	2.249	2.229	9.260	2.502	6.099	18.716	5.322	2.028
GPI	NM_000175.2	3.572	2.291	1.915	2.557	2.441	3.309	3.600	2.705	2.542	6.195	9.301	5.893	1.741
SESN2	NM_031459.3	2.253	2.282	2.209	2.652	2.096	2.684	2.260	3.271	8.118	5.254	3.689	2.295	1.579
ZSCAN20	BC011404.2	2.285	2.788	2.018	2.057	2.664	3.565	2.370	2.174	6.717	3.314	6.365	2.232	1.607
E2F6	NM_212540.1	2.870	2.421	2.083	2.885	5.970	3.577	2.371	2.443	3.561	4.602	2.740	7.388	1.591
C11orf73	BC018080.1	3.660	3.598	2.321	3.537	44.015	3.129	2.757	3.624	4.297	4.185	8.612	3.111	2.811
WDR66	BC036233.1	2.215	2.819	1.899	2.108	3.864	2.986	3.474	3.154	15.747	3.789	2.967	2.497	2.128
WRAP53	BC002336.2	2.698	2.734	2.150	3.415	7.962	2.494	2.534	3.958	3.336	4.368	3.234	8.300	1.645
SNRNP70	NM_003089.4	2.460	2.457	2.285	2.209	2.953	4.446	2.465	3.015	2.878	5.832	4.692	2.264	1.517
FAM84A	BC026346.1	27.762	34.167	9.797	14.456	39.402	33.482	14.316	17.838	71.358	112.712	37.048	23.380	2.028
BTN1A1	BC096312.3	2.786	2.151	1.912	3.064	2.837	2.772	2.830	3.975	3.071	3.167	2.847	10.610	1.619
PSMA3	NM_002788.2	4.406	4.016	2.381	4.364	6.410	4.937	3.521	4.043	7.781	99.246	5.133	4.404	4.466
C1orf94	NM_032884.2	3.641	3.888	2.711	11.565	5.539	4.214	5.038	5.059	41.171	57.236	4.912	4.501	2.928
KRTAP19-3	NM_181609	3.139	4.039	2.984	7.552	4.722	4.314	5.153	3.627	20.944	20.927	4.014	4.007	1.911
MPPED2	NM_001584.1	2.672	2.374	2.059	3.744	4.154	2.747	2.822	2.938	9.385	10.322	2.675	3.156	1.760
TRIM55	NM_184086.1	2.363	2.661	2.751	4.279	2.999	3.091	3.084	4.118	3.069	10.156	8.087	3.509	1.581
AMBN	NM_016519.4	2.210	2.665	1.936	3.760	6.053	3.110	2.429	3.370	2.902	7.149	6.003	3.753	1.644
DLG4	NM_001365.1	1.766	2.706	1.924	2.364	2.269	3.662	3.371	3.133	4.702	2.171	2.852	6.605	1.642

Gene Symbol	ID	HC1	HC2	HC3	HC4	w/o LNM1	w/o LNM2	w/o LNM3	w/o LNM4	w/ LNM1	w/ LNM2	w/ LNM3	w/ LNM4	FC (ESCC vs. HC)
SYAP1	NM_032796.2	2.543	3.453	2.337	2.709	2.724	11.687	2.718	2.995	4.093	5.433	2.647	9.269	1.882
TOM1	BC046151.1	3.887	4.926	2.777	4.868	8.820	7.535	3.082	4.194	47.771	4.591	14.211	2.745	2.824
SPSB2	NM_032641.1	3.065	2.747	2.167	2.913	4.324	4.504	2.578	2.811	3.494	10.948	2.833	2.292	1.551
MAGEA1	BC017555	5.730	4.220	2.171	11.147	8.791	5.427	149.141	7.948	5.233	6.595	5.075	114.449	6.504
NASP	NM_002482.2	3.975	3.003	2.981	13.443	14.783	6.757	4.955	4.404	11.837	4.871	4.765	22.874	1.608
ND	BC013935.1	2.788	3.307	3.036	5.210	4.179	5.444	2.931	4.354	4.161	12.649	8.700	3.882	1.614
IGHG1	BC075842.1	18.785	32.346	17.771	12.735	29.520	34.780	27.732	22.588	43.408	41.017	31.411	17.194	1.517
ZNF606	BC037209.1	3.564	3.026	2.293	3.316	5.412	5.064	2.891	3.284	4.161	12.052	2.568	2.875	1.570

Supplementary Table S6. The differentially expressed IgG autoantibodies between T1-stage ESCC that with or without lymph node metastasis

Gene Symbol	ID	w/o LNM1	w/o LNM2	w/o LNM3	w/o LNM4	w/ LNM1	w/ LNM2	w/ LNM3	w/ LNM4	FC (w/ LNM vs. w/o LNM)
BACH1	BC063307	7.873	2.935	4.564	4.592	13.341	38.809	16.844	10.877	4.001
UBL7	NM_032907.3	1.789	2.037	2.228	1.367	2.524	7.404	15.848	3.808	3.986
NUB1	NM_016118.3	2.277	2.297	2.168	2.101	6.935	2.909	3.756	2.691	1.842
PSMA3	NM_002788.2	3.306	4.843	3.932	3.981	5.604	81.807	4.639	5.279	6.060
LMOD3	BC039202.1	1.907	2.347	2.217	2.570	3.624	5.174	6.962	2.505	2.020
AKAP5	NM_004857.2	1.959	1.959	1.986	2.318	4.753	3.093	3.250	2.235	1.622
THUMPD1	NM_017736.3	3.901	18.712	2.675	3.677	473.087	2.088	150.213	50.600	23.338
ELP5	BC002762.2	4.516	2.626	2.301	3.722	8.889	45.068	21.211	3.862	6.003
RAB11FIP1	NM_001002233.1	12.756	14.134	5.013	22.019	98.583	52.963	96.092	12.579	4.826
SH3BP1	BC008282	1.786	7.088	8.790	6.772	19.018	30.835	44.588	6.982	4.151
RNF25	NM_022453.2	2.584	2.516	2.267	2.863	2.508	3.539	31.160	3.230	3.953
UBE2G1	NM_003342.4	2.289	2.372	2.384	2.802	28.828	3.028	3.710	2.140	3.829
NAP1L3	BC034954.2	3.587	3.101	2.323	2.334	13.847	6.739	5.898	2.242	2.532
ZNF503	NM_032772.3	2.350	2.039	2.584	2.568	6.047	9.334	2.332	6.078	2.493
KCNRG	NM_173605.1	2.823	2.649	2.776	3.172	5.461	7.378	11.545	2.808	2.381
TXLNB	AL834248	3.548	3.775	2.569	2.315	15.424	4.183	2.925	6.443	2.374
EPN1	NM_013333.2	3.629	5.179	6.868	7.348	10.033	10.259	25.069	3.235	2.111
EYA1	BC121798	5.990	11.779	13.301	9.684	40.958	19.492	19.623	5.912	2.110
GPI	NM_000175.2	2.441	3.309	3.600	2.705	2.542	6.195	9.301	5.893	1.985
GNPTG	BC014592.1	3.510	1.895	2.352	2.529	4.084	5.503	8.062	2.056	1.915
SESN2	NM_031459.3	2.096	2.684	2.260	3.271	8.118	5.254	3.689	2.295	1.877
PPAPDC3	NM_032728.2	2.839	2.548	2.598	3.165	3.523	4.824	2.811	8.108	1.728
C4BPB	NM_001017367.1	2.333	1.523	2.434	2.089	3.936	4.932	3.105	2.140	1.685
NAP1L5	NM_153757.1	1.979	2.453	2.614	2.613	3.266	3.167	5.663	2.601	1.522
MYLK	NM_053031.2	3.199	5.016	3.886	4.892	8.265	6.441	3.579	7.504	1.518
PDIA2	NM_006849	4.180	3.429	4.459	5.800	7.088	7.336	8.829	3.620	1.504
FADD	NM_003824.2	1.876	2.244	2.271	2.058	49.218	2.270	2.621	3.981	6.876
ZPR1	NM_003904.3	3.550	6.365	3.584	3.039	4.884	35.133	6.189	6.535	3.189
CCNJL	BC013353.2	2.297	2.431	3.253	2.349	7.184	13.524	2.736	2.682	2.529
KRTAP26-1	NM_203405.1	1.844	1.463	2.211	1.919	4.916	6.995	2.071	2.084	2.160

Gene Symbol	ID	w/o LNM1	w/o LNM2	w/o LNM3	w/o LNM4	w/ LNM1	w/ LNM2	w/ LNM3	w/ LNM4	FC (w/ LNM vs. w/o LNM)
PIAS2	NM_004671.2	2.081	1.690	1.881	1.854	7.717	2.656	3.633	2.060	2.140
JAM2	NM_021219.2	2.758	2.454	2.234	3.480	2.947	7.606	8.709	3.049	2.042
C20orf112	BC065370.1	1.673	1.620	1.921	1.695	1.773	3.981	1.901	4.991	1.831
CLGN	NM_004362.1	2.010	1.773	2.377	2.204	2.299	3.698	2.263	6.564	1.773
SLC25A39	NM_016016.1	1.860	1.940	2.016	1.785	3.601	5.820	2.074	1.963	1.771
ARSA	BC014210.2	1.875	1.520	2.028	1.615	3.609	4.342	1.958	2.028	1.696
GPX1	BC070258.1	1.909	1.785	1.957	1.879	3.238	4.296	2.035	1.944	1.529
MLST8	BC020499.1	2.039	1.997	2.306	1.859	3.593	4.406	2.169	2.171	1.505
GGA1	NM_001001561.1	2.220	2.531	2.367	2.051	1.960	49.593	2.925	4.730	6.457
BCL2L11	NM_138627.2	1.682	2.429	2.345	2.149	2.591	18.022	8.601	2.205	3.651
BAP1	NM_004656.2	2.145	2.108	2.037	2.071	17.390	4.528	2.287	2.036	3.138
ZBTB7A	BC084568.1	1.920	2.308	1.855	1.958	2.028	6.438	5.904	2.534	2.102
CTBP2	BC037900.2	2.039	1.779	2.252	2.251	4.690	2.492	2.223	7.006	1.972
ANGPT1	BC029406.1	1.759	1.452	1.871	1.873	3.462	5.725	1.911	1.830	1.859
AAGAB	BC058886.1	2.747	2.647	2.648	2.195	2.969	7.419	4.956	2.429	1.736
PEX7	BC031606.1	2.540	2.025	2.516	2.485	3.682	6.741	2.649	2.343	1.611
ART4	NM_021071.2	2.256	1.837	2.204	2.185	3.562	4.770	2.328	2.161	1.512
MAGI1	NM_004742	7.942	15.006	7.436	4.631	10.341	12.839	12.031	34.887	2.002
IVNS1ABP	BC067739	2.725	6.602	3.440	3.577	19.697	22.579	6.850	3.939	3.247
ZSCAN5A	BC043232.1	2.474	2.965	3.995	2.758	2.887	5.743	4.305	6.734	1.613
PAGE5	NM_001013435.1	3.845	4.611	3.433	9.463	12.826	11.111	1.954	79.497	4.936
SPA17	NM_017425.2	1.836	3.714	1.941	1.891	4.030	5.428	1.777	12.951	2.578
RBPJ	NM_203284.1	14.436	23.153	14.671	19.265	24.926	68.074	34.968	8.152	1.903
PODNL1	NM_024825.2	4.289	4.445	5.664	4.703	7.649	17.810	6.058	3.552	1.836
AFAP1L2	NM_001001936.1	5.565	4.294	1.947	2.346	6.482	8.483	5.567	2.650	1.638
ATP5B	NM_001686.3	5.218	3.802	3.096	4.788	6.024	5.410	2.968	57.335	4.244
MGC34796	BC034822.1	3.924	2.889	3.838	3.471	20.780	28.706	4.021	3.176	4.014
PPM1G	NM_002707.3	3.883	3.588	2.196	2.853	12.550	9.585	3.887	1.852	2.226
LASP1	NM_006148.1	2.225	2.200	1.944	5.202	2.724	55.473	19.498	2.382	6.921
BOP1	NM_015201.3	2.427	1.857	2.238	1.982	2.434	3.134	28.766	2.139	4.288
TRIM22	NM_006074.3	1.610	1.509	1.926	1.749	1.781	18.444	6.742	1.879	4.246

Gene Symbol	ID	w/o LNM1	w/o LNM2	w/o LNM3	w/o LNM4	w/ LNM1	w/ LNM2	w/ LNM3	w/ LNM4	FC (w/ LNM vs. w/o LNM)
GUK1	NM_000858.4	1.772	3.249	2.676	2.308	2.722	13.381	18.149	2.829	3.706
ATXN3	BC033711.1	5.214	29.918	3.568	12.114	92.147	34.882	25.934	11.438	3.235
TCF7L1	TCF7L1	2.796	2.412	2.522	3.740	3.190	4.915	2.846	15.686	2.322
USH1C	BC016057.1	2.371	5.727	4.343	2.254	4.275	9.847	14.711	3.488	2.200
ACSL4	NM_022977.1	5.349	3.436	2.410	3.451	3.860	16.053	3.569	7.798	2.136
C12orf45	BC032326.1	1.472	4.628	3.975	1.703	7.225	3.332	11.170	3.387	2.132
BCOR	NM_020926.2	3.103	2.045	2.654	2.176	6.887	8.589	2.490	2.575	2.059
RAB27A	NM_004580.3	1.792	2.671	1.947	1.698	6.839	2.011	4.892	1.972	1.938
MED22	NM_133640.3	6.189	9.994	4.341	5.227	17.923	5.751	7.606	17.238	1.884
IRAK1BP1	NM_001010844.1	2.038	3.446	2.469	2.529	2.880	6.091	7.469	2.563	1.813
METTL15	BC030997.1	2.065	1.757	1.906	1.798	3.585	5.805	1.975	1.854	1.757
MAGEA2	BC063681.1	3.137	2.036	2.422	2.638	3.098	3.240	2.618	8.597	1.715
KLHL26	BC026319	2.595	1.800	2.045	2.054	2.267	6.889	3.241	2.141	1.712
RPAIN	BC046349.1	2.079	2.295	2.640	2.757	4.854	5.579	2.595	2.616	1.601
TXNDC2	BC050132	3.398	6.796	3.029	3.052	6.407	7.926	8.028	3.393	1.582
HSF2	BC121050	1.801	1.894	2.532	2.039	2.459	4.726	3.128	2.155	1.508
ATP6V1B1	BC035978.1	2.763	2.114	2.779	3.572	4.390	6.535	3.062	2.935	1.507
WBSCR27	BC030295.2	2.684	2.313	2.300	1.968	13.555	21.005	2.600	2.147	4.243
GDI1	NM_001493.1	2.348	3.420	5.401	3.356	6.270	20.152	4.570	2.415	2.300
DTNBP1	NM_183041.1	2.098	4.700	3.194	2.577	4.216	14.357	4.802	2.049	2.023
ASMTL	BC002508.2	2.693	3.113	2.756	2.784	2.939	8.975	8.233	2.767	2.020
WDR4	NM_018669.4	2.354	2.126	1.901	2.113	8.883	2.354	3.901	2.015	2.019
CD83	BC030830.1	2.313	2.563	2.175	2.259	2.545	6.619	7.474	2.078	2.010
UROS	NM_000375.1	1.645	2.352	2.187	2.154	3.472	7.859	2.086	2.341	1.890
TRIM55	NM_184086.1	2.999	3.091	3.084	4.118	3.069	10.156	8.087	3.509	1.867
GNB1L	NM_053004.1	2.463	2.838	2.600	2.480	5.724	7.991	2.723	2.485	1.823
ZSCAN20	BC011404.2	2.664	3.565	2.370	2.174	6.717	3.314	6.365	2.232	1.729
PSMC3	NM_002804.3	4.499	5.713	2.844	4.351	5.273	10.947	4.096	8.907	1.679
KLK13	NM_015596.1	1.872	1.850	2.374	2.042	1.856	4.400	4.558	2.355	1.618
PDSS1	BC063635.1	1.937	1.982	2.086	1.914	1.942	4.532	3.781	2.080	1.558
TWF1	NM_002822.3	2.359	2.467	2.359	2.664	2.749	5.880	4.158	2.108	1.513

Gene Symbol	ID	w/o LNM1	w/o LNM2	w/o LNM3	w/o LNM4	w/ LNM1	w/ LNM2	w/ LNM3	w/ LNM4	FC (w/ LNM vs. w/o LNM)
MAGEA12	NM_005367.4	2.614	3.069	2.695	3.121	2.724	3.433	2.855	64.327	6.378
C1orf94	NM_032884.2	5.539	4.214	5.038	5.059	41.171	57.236	4.912	4.501	5.432
SIRT1	BC012499.1	2.970	13.285	17.076	6.468	117.484	5.359	65.943	14.080	5.097
TARBP2	NM_134323.1	2.420	2.504	2.485	2.933	41.323	3.755	2.084	2.204	4.774
SNAP91	NM_014841.1	2.688	3.098	2.042	2.037	5.245	36.853	1.846	1.803	4.638
TIRAP	NM_148910.2	3.202	2.460	2.424	3.453	2.536	26.475	17.666	2.361	4.250
KCTD5	NM_018992.1	2.213	2.179	2.089	2.086	29.085	3.245	1.853	1.798	4.200
AK6	NM_016283.4	3.508	4.814	6.305	3.908	5.135	15.427	2.855	50.832	4.006
TTK	NM_003318.3	2.343	2.123	2.397	2.604	2.122	3.663	28.686	2.261	3.880
PSMB4	BC000331.1	3.474	3.016	4.001	2.491	17.464	25.843	3.050	2.885	3.793
PDXK	BC003651.2	3.563	3.368	2.676	2.491	14.625	21.473	2.532	2.219	3.376
FMN1	BC103692.1	2.377	15.749	12.122	5.623	11.716	69.891	4.005	23.119	3.031
SPACA7	BC016750.2	4.905	5.359	2.519	3.222	33.928	3.295	8.233	2.984	3.027
TOM1	BC046151.1	8.820	7.535	3.082	4.194	47.771	4.591	14.211	2.745	2.933
PAGE2	NM_207339.1	3.333	5.065	6.629	9.190	17.335	3.414	2.725	46.111	2.873
KRTAP19-3	NM_181609	4.722	4.314	5.153	3.627	20.944	20.927	4.014	4.007	2.800
GARS	ENST00000389266	2.804	4.357	3.250	3.753	3.258	5.913	25.833	3.057	2.687
SEMA4G	BC020960.1	3.103	2.528	2.807	2.357	10.493	13.681	2.526	2.232	2.680
STK33	BC031231.1	2.084	1.952	2.606	2.040	2.237	3.316	14.984	1.891	2.583
KIAA1598	BC022348.1	2.724	6.603	7.551	4.565	34.887	4.471	4.502	11.190	2.567
TNS1	BC051304.1	2.847	5.762	2.267	2.395	9.870	2.583	2.428	18.284	2.499
SERPINI2	NM_006217.3	2.528	2.710	2.207	2.249	2.544	3.539	2.332	15.695	2.487
SPDL1	NM_017785.2	3.089	8.244	10.735	3.411	7.247	17.435	31.742	5.579	2.434
TPM2	NM_003289.3	5.369	4.870	15.191	14.909	44.130	6.207	39.102	7.845	2.412
UBE3A	BC002582.2	2.215	2.117	2.763	2.989	2.522	4.773	14.925	2.092	2.411
MBIP	BC005197.1	3.601	2.685	2.131	2.333	13.696	7.006	2.771	2.139	2.383
SNX9	NM_016224.3	3.730	3.100	3.985	5.259	7.653	23.221	4.349	2.950	2.375
CRYL1	NM_015974.2	3.945	3.664	2.141	2.771	3.119	5.862	18.161	2.461	2.364
SLC7A6OS	BC013778.1	2.269	6.680	6.723	8.625	14.395	3.985	6.223	32.350	2.344
LOC402578	XM_380135.2	3.344	2.173	2.171	1.833	5.724	12.465	1.917	2.154	2.338
AK7	BC035256.1	2.571	5.685	2.122	2.549	11.613	3.736	12.660	2.110	2.330

Gene Symbol	ID	w/o LNM1	w/o LNM2	w/o LNM3	w/o LNM4	w/ LNM1	w/ LNM2	w/ LNM3	w/ LNM4	FC (w/ LNM vs. w/o LNM)
FAM84A	BC026346.1	39.402	33.482	14.316	17.838	71.358	112.712	37.048	23.380	2.328
RBFOX2	BC013115.1	3.512	2.412	2.897	3.328	8.787	13.251	3.279	2.959	2.327
SMUG1	BC000417.2	3.052	2.367	2.797	2.260	7.991	11.232	2.623	2.140	2.290
SPRTN	BC015740.1	2.265	1.946	2.921	2.155	4.998	11.320	2.423	2.208	2.256
C6orf1	BC047919.1	2.883	2.728	3.317	2.775	8.362	12.466	2.761	2.666	2.243
TRIM16	NM_006470.3	2.715	1.965	2.693	3.268	6.475	1.975	12.665	2.535	2.222
TGIF2LX	NM_138960.3	2.008	2.327	2.017	2.281	2.166	6.616	8.292	1.893	2.197
LYPD3	NM_014400.2	2.400	2.175	2.249	2.071	2.228	8.125	6.818	1.981	2.153
SLC25A28	NM_031212.2	2.187	1.775	2.148	2.001	5.047	8.245	1.928	2.061	2.131
C9orf24	NM_032596.3	6.986	3.231	3.680	3.843	12.027	19.194	3.382	2.684	2.102
ARL2BP	BC094878.1	1.538	2.866	2.645	2.165	2.063	7.931	7.193	2.168	2.101
LOC494141	BC034967.1	2.799	2.278	2.334	2.263	6.307	8.930	2.249	2.148	2.029
MPPED2	NM_001584.1	4.154	2.747	2.822	2.938	9.385	10.322	2.675	3.156	2.017
BCAS2	NM_005872.2	6.025	3.369	2.826	2.326	9.537	12.809	4.257	2.264	1.984
Ucp3	BC008392.1	2.556	2.006	2.639	2.950	5.378	9.131	2.683	2.448	1.935
MTMR4	NM_004687.3	2.069	2.284	2.033	1.774	1.822	7.566	1.878	4.378	1.917
KRTAP13-1	BC113538.1	2.211	1.859	2.466	2.001	4.440	7.842	1.990	2.031	1.910
RHOXF2	NM_032498.1	5.030	4.271	3.492	4.343	11.283	13.974	3.889	3.568	1.909
C2orf15	NM_144706.2	3.782	2.599	2.766	3.771	13.425	4.688	3.032	2.853	1.858
CABP4	BC033167.1	2.419	2.606	2.299	1.984	2.312	7.017	5.729	2.227	1.857
MPZL1	BC019890.1	2.568	3.050	2.314	2.298	6.603	7.893	2.234	2.180	1.848
NCAPG2	BC020560.1	2.209	1.974	2.355	2.360	4.332	7.239	2.335	2.278	1.819
LOC389687	XM_374281.1	3.567	2.219	2.673	3.064	5.532	9.307	3.138	2.505	1.778
CTDP1	NM_004715.4	3.193	2.974	2.287	2.407	7.966	6.328	1.984	2.931	1.769
C1orf158	NM_152290.1	4.778	2.611	3.094	3.293	6.732	10.879	3.680	2.753	1.745
NOV	BC015028.1	2.350	2.653	2.452	2.706	9.453	3.014	2.700	2.461	1.735
USP4	NM_003363.2	3.490	3.079	2.442	1.963	7.814	6.289	2.017	2.410	1.689
THEM5	NM_182578.1	2.382	2.330	2.435	3.238	2.627	7.493	2.334	4.981	1.679
CYB5D1	NM_144607.2	2.286	2.586	2.152	1.926	4.149	6.536	2.231	2.098	1.678
CCDC108	BC047637.1	4.375	2.617	2.837	3.741	7.111	9.637	2.969	2.687	1.651
TOR1AIP2	NM_022347.1	2.670	1.902	2.068	2.055	4.320	5.735	2.170	2.026	1.639

Gene Symbol	ID	w/o LNM1	w/o LNM2	w/o LNM3	w/o LNM4	w/ LNM1	w/ LNM2	w/ LNM3	w/ LNM4	FC (w/ LNM vs. w/o LNM)
ARFGAP1	NM_175609.1	2.345	3.509	4.008	3.072	2.854	7.041	8.616	2.360	1.614
CCT8	NM_006585.2	2.819	3.266	2.939	2.627	2.646	6.129	7.406	2.509	1.604
TIMP4	NM_003256.2	3.146	2.511	2.143	2.445	4.979	6.866	2.458	2.085	1.600
GYG2	BC023152.1	3.271	3.288	2.551	3.272	10.209	4.594	2.573	2.389	1.596
TIMP1	BC007097.1	3.213	2.252	2.151	2.316	4.336	7.380	2.095	1.954	1.587
PSMB8	NM_004159.4	2.631	2.434	2.418	2.500	5.051	5.842	2.561	2.214	1.569
NIT1	BC046149.1	2.972	2.303	2.867	3.144	5.182	7.079	2.872	2.500	1.562
ZFP91	ZFP91	2.066	3.323	2.422	2.300	2.157	6.324	1.985	5.321	1.561
PLSCR3	BC011735.2	2.633	2.478	2.361	2.778	4.524	6.461	2.555	2.297	1.545
NOTCH2NL	NM_203458.2	2.191	2.507	2.318	2.071	4.075	5.856	1.986	2.109	1.544
NAP1L1	NM_004537.3	2.142	2.437	3.386	4.028	2.926	5.156	8.357	2.040	1.541
GTF2E1	NM_005513.2	1.798	1.710	2.029	1.959	3.582	1.678	4.219	1.912	1.520
SLC25A42	NM_178526.1	3.218	2.271	2.389	2.383	5.185	5.845	2.262	2.296	1.519
GALK2	NM_002044.2	2.430	2.275	2.112	2.331	5.111	4.338	2.282	2.095	1.511
Smarca2	BC075641	1.647	1.456	1.786	2.396	1.697	3.576	3.814	1.845	1.501
PI4K2B	BC051749.1	3.257	2.058	2.570	4.522	3.376	4.171	3.849	13.751	2.027
CAST	NM_173060.2	3.593	27.277	3.064	11.025	24.898	17.410	28.864	11.162	1.831
PNMA2	BC036489.1	3.799	4.311	10.668	3.954	4.728	6.842	22.827	4.372	1.705
ALDH1L1	BC027241.1	2.897	3.516	2.370	2.370	2.666	9.551	3.405	3.117	1.680
BTN1A1	BC096312.3	2.837	2.772	2.830	3.975	3.071	3.167	2.847	10.610	1.586
RDH10	NM_172037.2	3.279	4.974	4.047	7.320	7.007	14.507	6.778	3.905	1.641
AAMP	BC014122.2	3.888	8.933	3.799	169.080	3.204	3.768	3.386	3.703	0.076
PTPN12	NM_002835.2	6.238	24.136	3.596	10.586	2.951	3.561	2.721	2.104	0.254
MUC15	NM_145650.2	32.817	7.924	7.820	13.350	5.450	4.238	6.340	4.280	0.328
KIZ	BC045826.1	33.612	10.431	8.035	7.782	2.949	7.085	6.717	4.475	0.355
SCG2	BC022509.1	5.326	9.973	6.773	3.752	2.896	2.136	2.593	2.053	0.375
MARCKSL1	BC066915.1	3.501	8.852	3.186	4.864	2.159	1.959	2.045	2.276	0.414
HSCB	NM_172002.3	8.289	11.117	6.616	5.806	3.928	4.569	5.308	4.240	0.567
CAMKK1	NM_032294.2	3.114	6.604	4.862	3.212	2.406	2.709	2.542	2.510	0.571
OXR1	BC032710	4.626	5.683	2.914	2.902	1.657	1.601	1.885	1.828	0.432
LYSMD4	BC084545.1	8.052	8.318	4.808	16.981	3.919	4.016	3.274	5.438	0.436

Gene Symbol	ID	w/o LNM1	w/o LNM2	w/o LNM3	w/o LNM4	w/ LNM1	w/ LNM2	w/ LNM3	w/ LNM4	FC (w/ LNM vs. w/o LNM)
CAAP1	NM_024828.2	5.035	6.272	6.867	4.523	1.995	2.238	2.857	4.592	0.515
ZNF330	NM_014487.2	7.278	4.364	3.035	2.461	2.317	2.699	2.264	2.165	0.551
PRMT3	PRMT3	4.336	5.208	2.437	3.227	2.137	2.592	2.237	2.133	0.598
LDLRAD4	NM_181481.2	6.977	3.913	3.256	4.948	3.774	2.519	2.867	2.772	0.625
LIX1	NM_153234.3	6.069	3.520	2.986	5.245	2.941	3.052	2.802	2.469	0.632
FAM129B	BC067366.1	3.530	7.679	2.699	91.764	3.088	3.027	2.821	2.714	0.110
STXBP4	ENST00000299341	48.487	3.125	6.144	26.705	3.489	3.767	3.994	3.072	0.170
DUSP14	NM_007026.1	12.669	1.759	10.084	7.024	2.344	2.372	2.003	1.961	0.275
PTGES3L-AARSD1	NM_025267.2	4.961	22.644	4.535	16.902	4.584	3.457	4.534	2.234	0.302
SNX1	NM_003099.3	2.054	8.848	21.533	12.537	4.678	3.603	2.770	5.323	0.364
NUDT16	BC031215.1	16.311	4.590	6.879	2.680	2.767	3.931	2.812	3.180	0.417
HDGFL1	NM_138574.2	16.138	4.974	5.181	2.032	3.244	3.220	2.096	4.190	0.450
NBPF22P	BC050328.1	12.332	3.691	4.231	2.537	2.837	3.168	2.478	2.748	0.493
SOX6	BC047064.1	12.607	4.235	5.346	2.373	3.082	3.746	2.954	3.072	0.523
ZKSCAN8	BC096284.3	7.485	3.087	5.920	2.348	2.071	2.537	2.577	2.826	0.531
CTAG1A	NM_139250	4.134	3.829	9.575	2.578	3.074	2.940	2.293	2.528	0.539
STARD4	NM_139164.1	2.116	4.794	6.163	3.963	2.580	2.923	2.441	2.043	0.586
UBA5	NM_024818.2	4.974	2.280	5.308	3.628	1.984	2.983	2.697	1.985	0.596
ITGB8	NM_002214.2	7.755	7.231	3.454	7.831	3.622	5.045	4.792	2.821	0.620
ACSBG1	BC009289.2	4.849	3.027	6.854	4.525	3.197	3.080	2.906	3.047	0.635
PROL1	NM_021225.4	10.474	8.234	3.951	8.716	3.911	6.482	5.514	4.074	0.637
CD69	NM_001781.1	3.746	3.438	3.051	8.427	2.801	3.044	3.150	3.074	0.647
MUC3A	XM_003846410.1	4.772	5.452	2.552	6.167	3.000	3.391	2.700	3.261	0.652
DEFB118	DEFB118	3.342	2.136	7.412	2.796	1.984	1.945	2.280	2.073	0.528
EIF4EBP1	NM_004095.2	2.075	4.951	2.775	4.633	1.865	2.206	1.917	1.915	0.548
C10orf118	BC030557.1	11.483	5.523	7.185	5.390	2.412	6.570	2.982	4.234	0.548
HELQ	BC011863.2	2.072	2.628	3.233	27.679	2.431	2.417	2.207	2.255	0.261
MUC20	BC029267.1	2.718	7.902	2.154	3.091	2.050	2.329	2.173	2.053	0.542
WBP11	NM_016312.2	1.859	2.642	5.436	4.431	2.013	2.235	2.193	1.840	0.576
FARSB	BC017783.1	4.103	1.878	2.395	4.815	1.945	2.174	1.978	2.027	0.616

Gene Symbol	ID	w/o LNM1	w/o LNM2	w/o LNM3	w/o LNM4	w/ LNM1	w/ LNM2	w/ LNM3	w/ LNM4	FC (w/ LNM vs. w/o LNM)
IL1RAPL1	BC111918	3.071	5.654	2.152	2.610	1.965	2.332	2.336	2.246	0.658
MBLAC1	BC031288.1	15.081	4.514	2.352	3.530	3.316	2.749	2.277	2.185	0.413
FAM131C	BC016848.1	29.561	20.685	6.045	10.244	5.840	9.755	5.514	7.143	0.425
PRH2	BC095488.1	3.443	20.109	5.040	5.249	3.560	4.865	3.614	4.035	0.475
PUS7L	NM_031292.2	2.538	5.529	17.142	6.228	4.654	3.944	5.020	2.916	0.526
RNF157	NM_052916	4.932	2.568	7.887	9.696	3.427	4.366	3.497	2.304	0.542
NUDT5	NM_014142.2	5.984	9.041	2.866	4.359	4.092	3.559	3.032	3.432	0.634
FNIP2	NM_020840	3.761	4.325	11.415	2.554	3.630	2.924	2.219	3.656	0.564
ECI2	NM_206836.1	3.777	8.742	2.461	3.521	2.699	3.304	2.703	3.306	0.649
SMYD3	NM_022743.1	40.768	8.023	117.115	5.686	3.988	3.495	30.180	4.788	0.247
ZNF671	BC025728.1	20.974	3.707	2.916	14.436	2.804	4.035	3.005	2.567	0.295
PRRT2	BC053594.1	7.303	13.377	5.520	34.697	8.575	4.580	4.496	5.671	0.383
SOX9	NM_000346.2	5.580	11.610	2.366	2.198	2.138	2.249	2.677	2.021	0.418
ANP32A	BC007200.1	6.028	12.652	19.702	4.972	11.047	3.214	2.357	3.041	0.453
CSF1	NM_000757.3	14.558	4.533	4.173	6.838	4.442	3.412	4.648	3.600	0.535
VPS4B	NM_004869.3	2.225	3.129	2.402	7.374	2.437	2.101	1.996	2.325	0.586
SPN	NM_003123.3	124.525	5.833	33.592	75.512	17.279	6.897	35.109	8.557	0.283
PRRG1	NM_000950.1	25.662	2.411	6.301	16.459	3.290	2.726	7.384	4.204	0.346
BCL7B	NM_138707.1	2.393	6.291	5.620	2.223	2.135	2.252	2.231	2.405	0.546
TMEM129	NM_138385.2	4.593	6.646	2.598	1.932	2.600	2.119	1.851	2.104	0.550
G3BP2	NM_203504.1	2.316	2.086	4.802	5.854	2.212	2.295	2.114	1.917	0.567
PRLR	NM_000949.2	6.272	2.287	2.881	5.283	2.372	2.029	2.706	2.759	0.590
LETM1	NM_012318.1	6.439	4.961	1.945	3.057	3.198	1.942	2.207	2.458	0.598
FAM153A	NM_173663.1	22.058	104.620	1.887	2.751	4.450	3.433	3.146	2.981	0.107
PPM1B	NM_177968.2	34.139	4.098	1.993	2.024	2.123	2.017	3.017	2.071	0.218
Supt6	BC072657	65.696	12.241	88.074	2.035	8.328	5.776	19.507	9.165	0.255
PAF1	NM_019088.2	24.333	29.831	3.972	4.194	3.183	4.144	3.815	4.849	0.257
ZYX	NM_001010972.1	7.688	14.668	2.000	2.005	2.140	2.417	2.641	1.937	0.347
WASL	NM_003941.2	4.225	19.888	1.899	2.909	1.877	3.229	2.739	2.322	0.352
ATF6	BC014969.1	26.634	16.387	2.167	3.648	6.683	6.242	2.648	2.070	0.361
C14orf37	NM_001001872.2	50.621	2.608	8.143	23.309	5.915	4.332	12.687	7.926	0.364

Gene Symbol	ID	w/o LNM1	w/o LNM2	w/o LNM3	w/o LNM4	w/ LNM1	w/ LNM2	w/ LNM3	w/ LNM4	FC (w/ LNM vs. w/o LNM)
PMEPA1	NM_199169.1	183.083	15.470	46.774	169.545	36.225	16.209	76.911	31.066	0.387
GBP5	NM_052942.2	3.861	28.221	3.009	42.038	5.435	8.200	13.848	2.672	0.391
JUNB	NM_002229.2	322.623	135.141	6.261	78.915	3.338	101.945	88.959	37.469	0.427
FAM53C	NM_016605.1	9.292	11.589	1.855	2.019	4.283	2.237	2.215	2.028	0.435
PPP1R13L	BC064913.1	12.552	19.951	2.481	3.376	2.699	4.196	7.853	2.527	0.450
SLX4	BC036335.1	3.694	12.561	1.842	2.208	2.550	2.250	2.478	1.925	0.453
ELL3	NM_025165.2	7.060	24.159	9.671	31.006	6.293	10.301	12.475	3.864	0.458
RSC1A1	NM_006511.1	4.308	1.744	1.813	8.386	1.843	1.692	2.035	1.902	0.460
PRRG2	NM_000951.1	14.001	2.201	3.318	5.912	3.697	2.305	3.544	2.350	0.468
PPRC1	BC063829	6.834	2.511	12.124	1.903	2.887	3.103	2.671	2.364	0.472
HIP1	NM_005338.4	5.283	1.757	2.031	8.156	1.809	1.984	2.233	2.281	0.482
STAT4	BC031212.1	8.918	9.600	2.584	3.231	2.778	3.664	2.596	2.970	0.493
SORBS2	NM_021069.3	9.641	4.761	1.854	2.392	1.680	2.751	2.682	2.152	0.497
LIME1	BC017016.1	8.276	1.614	2.338	3.824	1.904	1.841	2.255	2.279	0.516
CXCR3	NM_001504.1	9.759	4.783	2.161	2.427	2.712	2.733	2.218	2.238	0.518
GPBP1	BC000267.1	21.078	16.018	2.341	1.936	4.241	2.901	5.411	9.109	0.524
NLN	BC004985.1	6.160	14.820	3.027	3.900	3.328	3.935	4.181	3.221	0.525
ERVFRD-1	BC068585.1	2.225	3.682	3.482	13.833	2.949	3.387	3.552	2.670	0.541
EPB41	BC039079.1	29.499	6.088	28.011	3.975	13.705	16.960	3.105	2.883	0.542
DDX19A	NM_018332.3	3.938	11.695	4.023	11.234	4.414	4.213	4.992	3.171	0.544
BAALCOS	ENST00000436771	5.905	13.771	3.352	3.730	3.042	4.011	3.984	3.656	0.549
KDM5A	JARID1A	1.684	4.246	2.030	5.415	1.810	1.961	1.840	2.009	0.570
ZFYVE27	NM_001002262.1	3.949	1.622	6.188	1.951	1.871	2.207	1.987	1.994	0.588
ZDHHC9	NM_001008222.1	5.410	10.762	2.834	3.791	4.086	3.758	3.230	2.998	0.617
CDH13	NM_001257.3	7.039	1.718	2.508	4.234	1.981	1.945	3.082	2.660	0.624
PPIP5K1	BC050263.1	2.041	3.017	6.476	2.006	2.103	2.462	1.961	1.929	0.624
DENND1C	NM_024898.2	2.152	2.234	5.929	4.172	2.250	2.399	2.360	2.079	0.627
TEX12	NM_031275.3	4.372	8.070	2.661	2.778	2.865	3.264	2.563	2.562	0.629
ECE1	NM_001397.1	12.688	40.114	10.232	41.741	23.866	18.181	11.913	12.518	0.634
FSCN1	BC006304.2	3.808	9.009	2.432	2.962	2.690	3.046	3.118	2.733	0.636
KDM4C	JMJD2C	7.591	6.260	2.152	2.674	4.266	4.022	1.991	2.071	0.661

Gene Symbol	ID	w/o LNM1	w/o LNM2	w/o LNM3	w/o LNM4	w/ LNM1	w/ LNM2	w/ LNM3	w/ LNM4	FC (w/ LNM vs. w/o LNM)
LRP10	NM_014045.3	8.245	2.727	2.839	4.426	2.953	2.676	3.487	3.036	0.666
RTN4	NM_207521.1	7.922	50.283	5.132	5.358	5.643	12.610	2.350	2.979	0.343
PRKAR2B	BC075800.1	3.503	52.329	20.679	4.053	22.414	4.262	2.629	2.621	0.396
ERBB4	NM_001042599.1	6.751	6.294	29.899	3.769	3.253	12.651	3.555	3.263	0.486
PRKCZ	NM_002744.4	3.271	5.946	32.239	3.128	2.874	2.907	14.126	3.165	0.518
BZW2	NM_014038.1	4.220	8.618	3.662	4.702	3.516	4.717	3.262	2.431	0.657
PALM2	NM_001037293.1	29.660	35.582	3.811	45.031	41.168	8.780	6.586	5.157	0.541

Supplementary Table S7. The 41 overlapped tumor-related IgG autoantibodies in T1-stage ESCC with LNM compared with those without LNM

Gene Symbol	Protein location	FC (w/ LNM vs. w/o LNM)	Sensitivity	Specificity
BACH1	nucleus/cytoplasm/cytosol	4.00	1	1
PODNL1	cytoplasm	1.84	0.75	1
ERBB4	Nucleus/Mitochondrion	2.06	0.5	0
FAM84A	nucleus/nucleoplasm	2.33	0.5	1
PTGES3L-AARSD1	Nuclear membrane/Cytosol	3.31	0.25	0.25
FAM153A	nucleus/cytosol	9.37	0.25	0.5
ATF6	nucleus	2.77	0.25	0.5
IVNS1ABP	nucleoplasm	3.25	0.75	1
MPPED2	nucleoli/vesicles/mitochondria	2.02	0.5	1
PPP1R13L	Cytosol	2.22	0.25	0.5
CTAG1A	plasma membrane/cytosol/nucleus	1.86	0.25	0.25
MARCKSL1	nucleus/cytosol	2.42	1	0
BTN1A1	predicted membrane proteins	1.59	0.75	0.75
MAGEA2	nucleus	1.72	0.5	1
SNX1	Endosomes/Lysosomes	2.75	0.25	0.25
ZPR1	nucleolus/nucleoplasm/nucleus/cytoplasm	3.19	0.5	1
TPM2	cytosol	2.41	0.5	1
PSMA3	nucleoplasm/nucleus/cytoplasm/cytosol	6.16	0.5	1
PNMA2	nucleolus	1.71	1	0.75
CRYL1	nucleolus/nucleus/cytoplasm/cytosol	2.36	0.5	1
FAM131C	Nucleoli/Intermediate filaments/Cytosol	2.36	0	0.5
SPDL1	nucleus/cytosol	2.43	0.5	1
ATXN3	nucleoplasm/nucleus/cytoplasm/cytosol	3.24	0.5	1
PAGE5	cytoplasm	4.94	0.75	1
C1orf94	nucleoplasm/cytosol	5.43	0.5	1
PAGE2	predicted intracellular proteins	2.87	0.5	1
ITGB8	Plasma membrane/Cytosol	1.61	0.25	0.25
TOM1	cytoplasm/cytosol	2.93	0.5	1
GBP5	golgi apparatus/endoplasmic reticuium	2.56	0.25	0.5
GPI	nucleoplasm/cytoplasm/cytosol	1.99	0.75	1
MED22	cytoplasm	1.88	0.5	1

Gene Symbol	Protein location	FC (w/ LNM vs. w/o LNM)	Sensitivity	Specificity
ZSCAN20	nucleus	1.73	0.5	1
SESN2	nucleus/cytoplasm/cytosol	1.88	0.75	1
PALM2	Nucleoplasm/Nuclear bodies/Plasma membrane	1.85	0.5	0.25
PI4K2B	cytoplasm/cytosol	2.03	1	0.75
USH1C	cytoplasm/cytosol	2.2	0.5	1
SPA17	cytoplasm	2.58	0.75	1
KRTAP19-3	cytosol	2.8	0.5	1
TRIM55	nucleus/cytoplasm	1.87	0.5	1
PROL1	extracellular/plasma membrane/cytosol	1.57	0.25	0.25
THUMPD1	nucleoplasm	23.34	0.75	1
