# Supplementary material for: BACH1-induced ferroptosis drives lymphatic metastasis by repressing the biosynthesis of monounsaturated fatty acids
Source: Cell Death Dis. 2023 Jan 20;14(1):48. doi: 10.1038/s41419-023-05571-z (PMC9860034; doi:10.1038/s41419-023-05571-z)

Figure 2A:

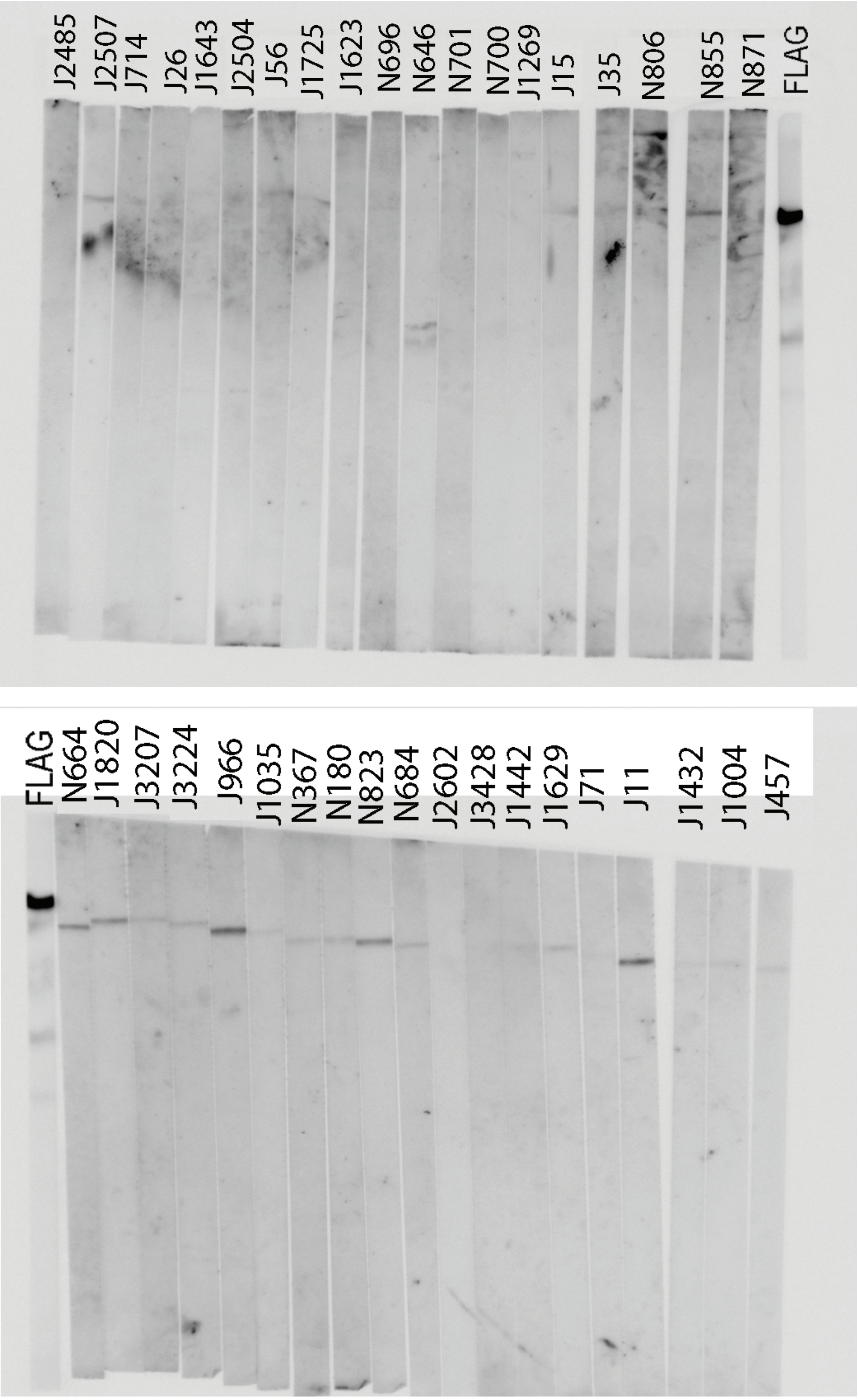
J2485
J2507
J714
J26
J1643
J2504
J56
J1725
J1623
N696
N646
N701
N700
J1269
J15
J35
N806
N855
N871
FLAG
FLAG
N664
J1820
J3207
J3224
J966
J1035
N367
N180
N823
N684
J2602
J3428
J1442
J1629
J71
J11
J1432
J1004
J457

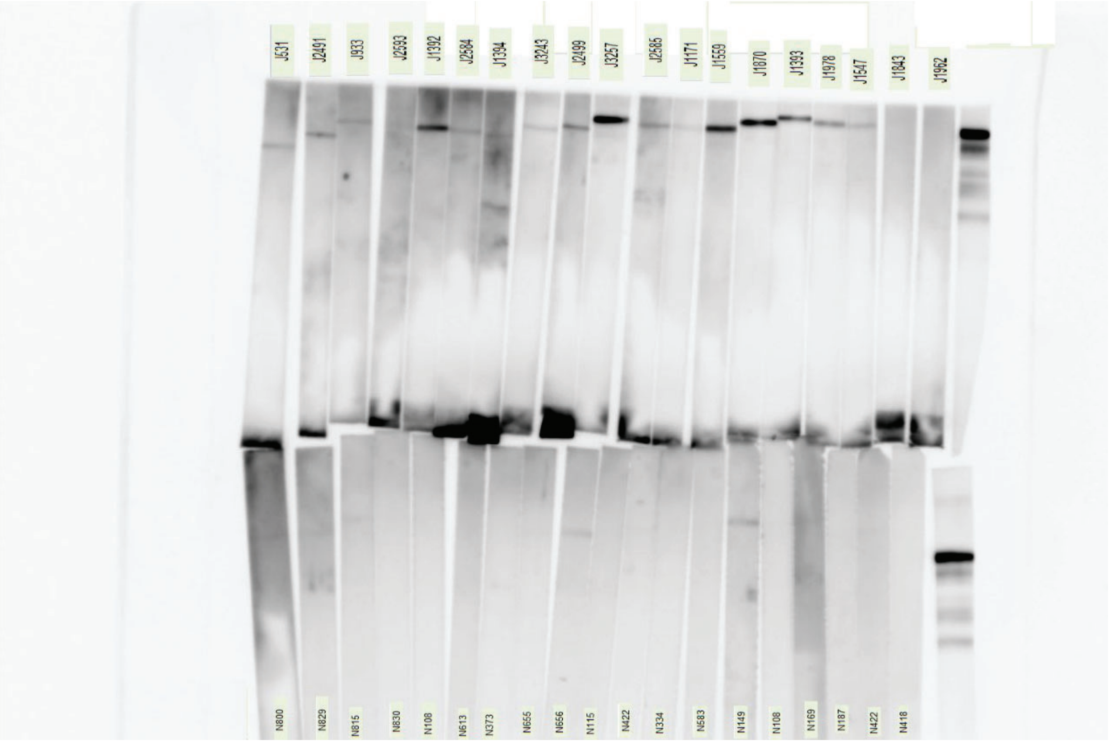

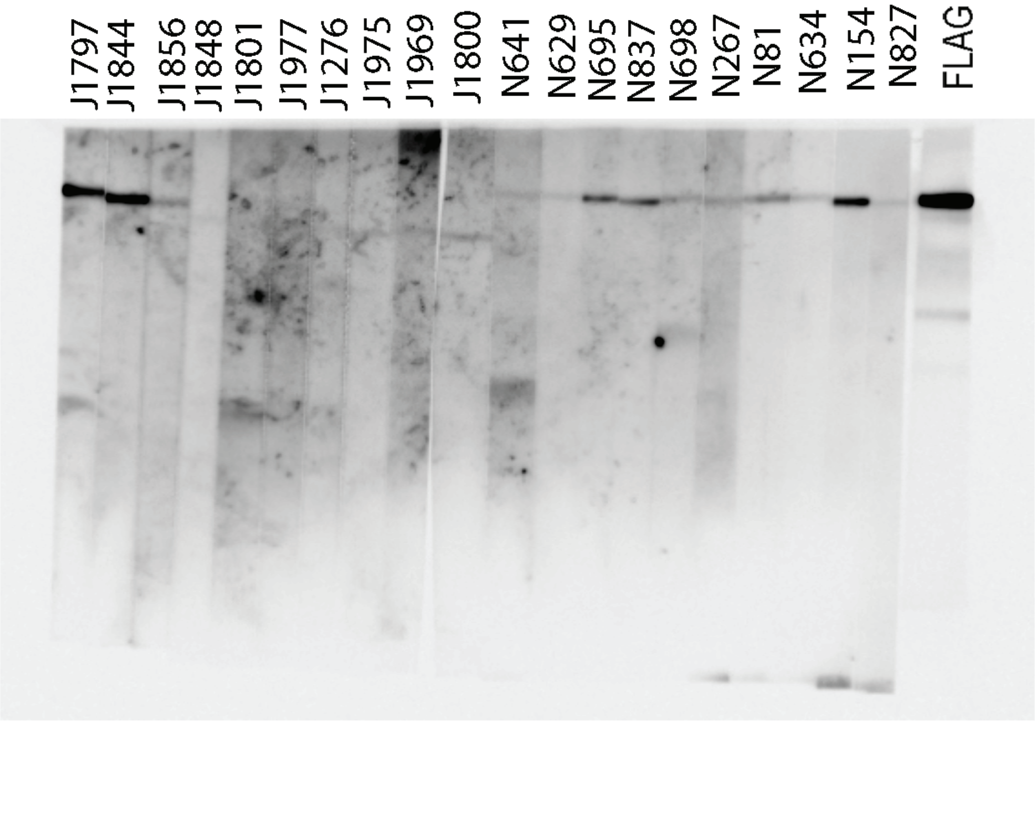
J1797
J1844
J1856
J1848
J1801
J1977
J1276
J1975
J1969
J1800
N641
N629
N695
N837
N698
N267
N81
N634
N154
N827
FLAG

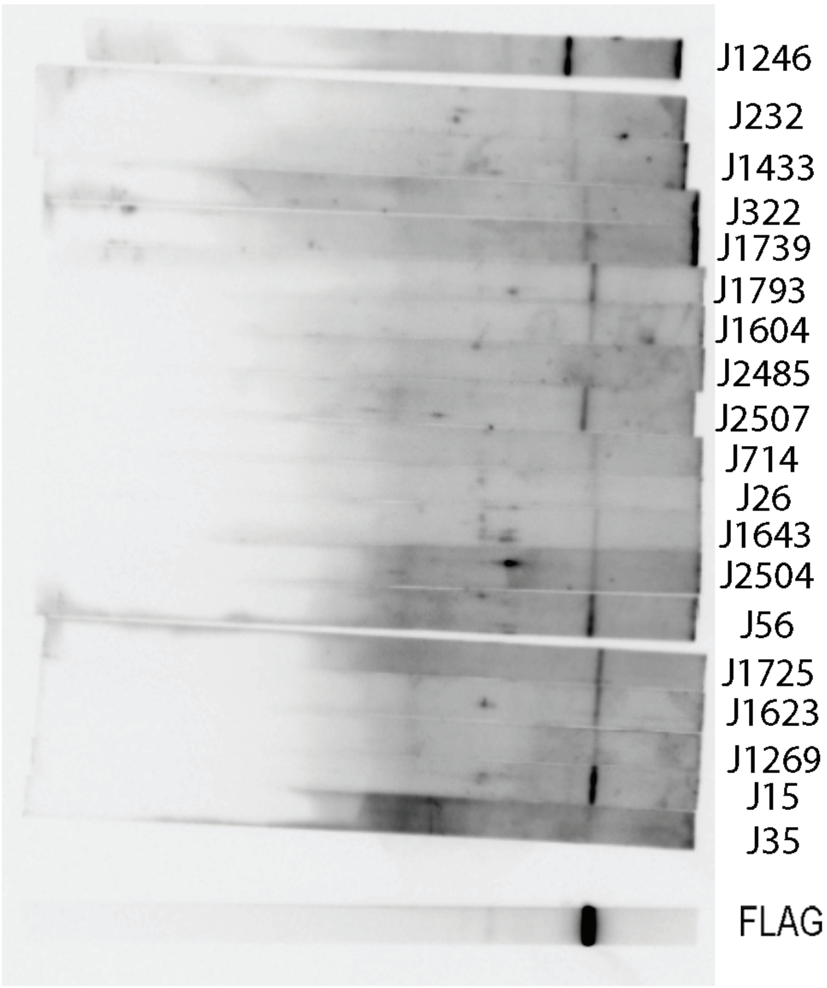
J1246
J232
J1433
J322
J1739
J1793
J1604
J2485
J2507
J714
J26
J1643
J2504
J56
J1725
J1623
J1269
J15
J35
FLAG

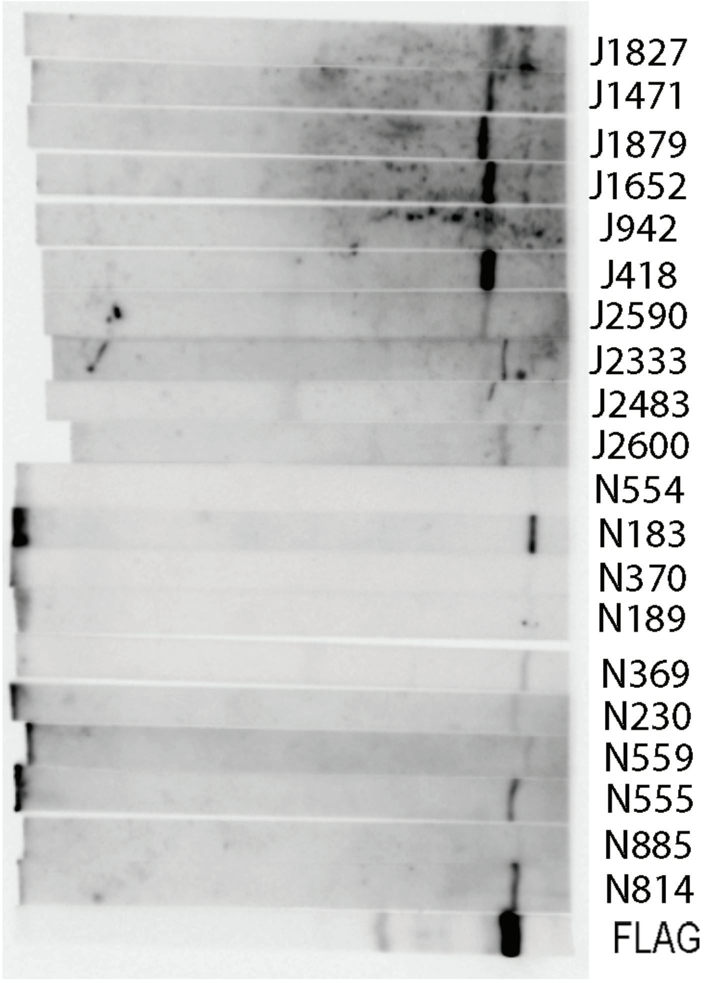
J1827
J1471
J1879
J1652
J942
J418
J2590
J2333
J2483
J2600
N554
N183
N370
N189
N369
N230
N559
N555
N885
N814
FLAG

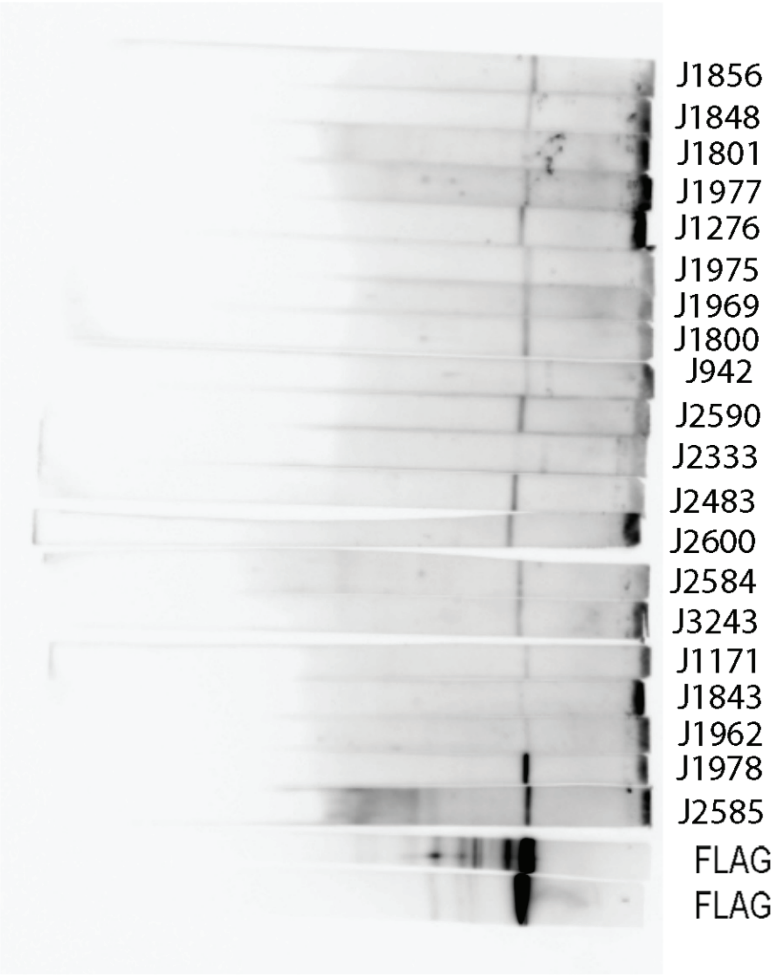
J1856
J1848
J1801
J1977
J1276
J1975
J1969
J1800
J942
J2590
J2333
J2483
J2600
J2584
J3243
J1171
J1843
J1962
J1978
J2585
FLAG
FLAG

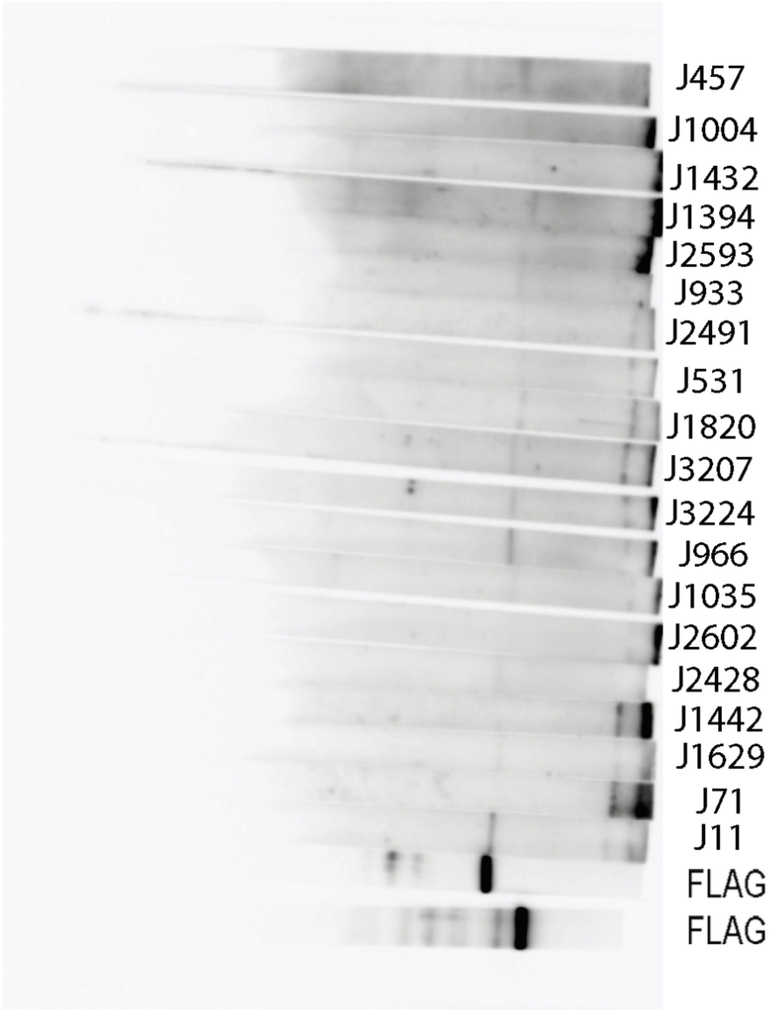
J457
J1004
J1432
J1394
J2593
J933
J2491
J531
J1820
J3207
J3224
J966
J1035
J2602
J2428
J1442
J1629
J71
J11
FLAG
FLAG

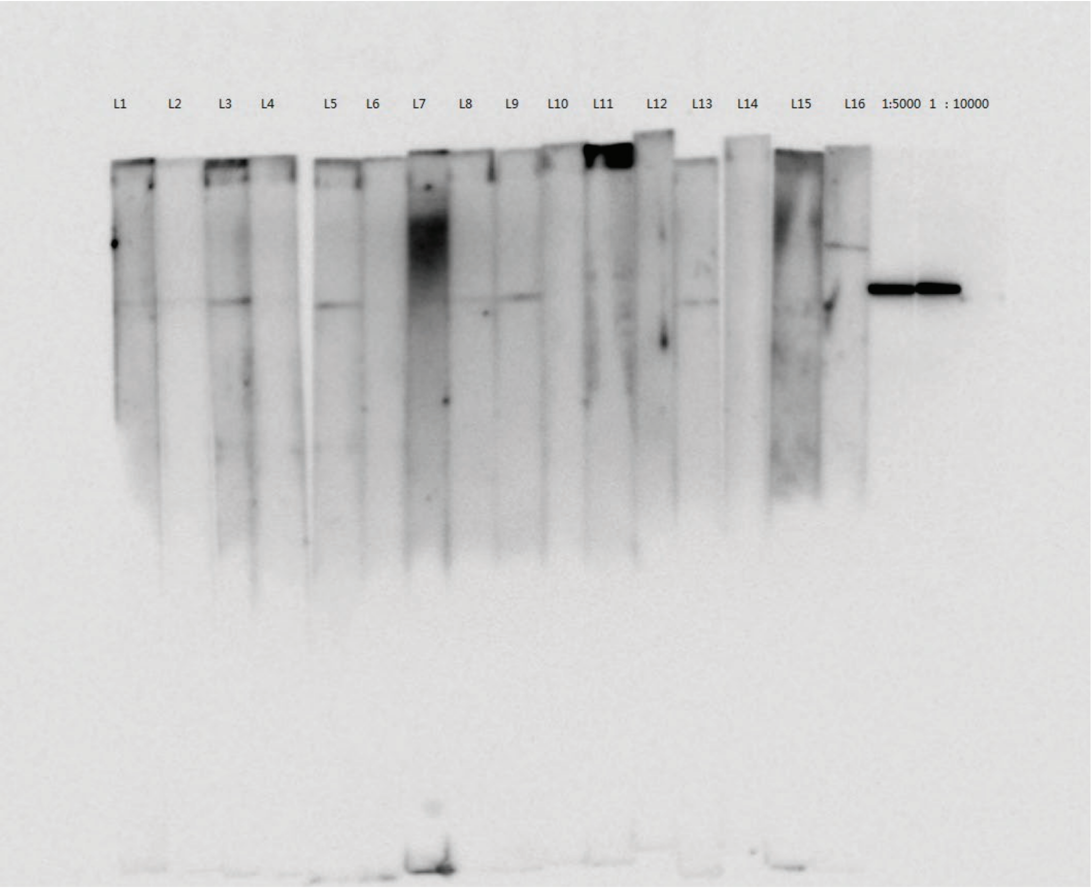
L1 L2 L3 L4 L5 L6 L7 L8 L9 L10 L11 L12 L13 L14 L15 L16 1:5000 1 : 10000

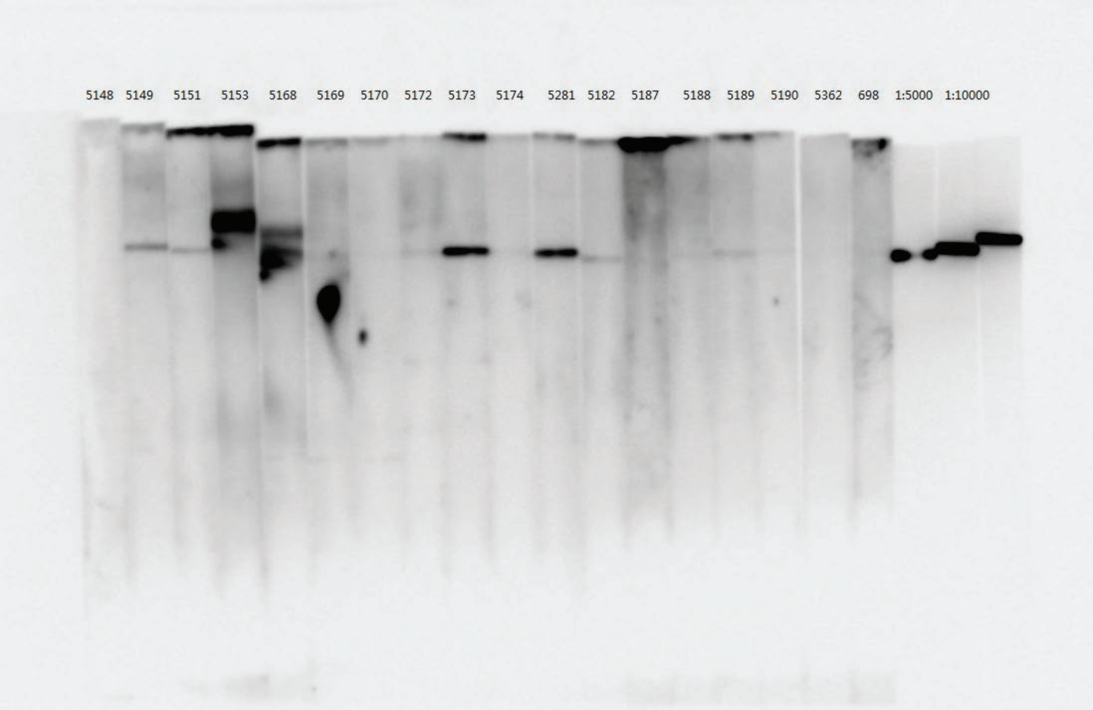
5148 5149 5151 5153 5168 5169 5170 5172 5173 5174 5281 5182 5187 5188 5189 5190 5362 698 1:5000 1:10000

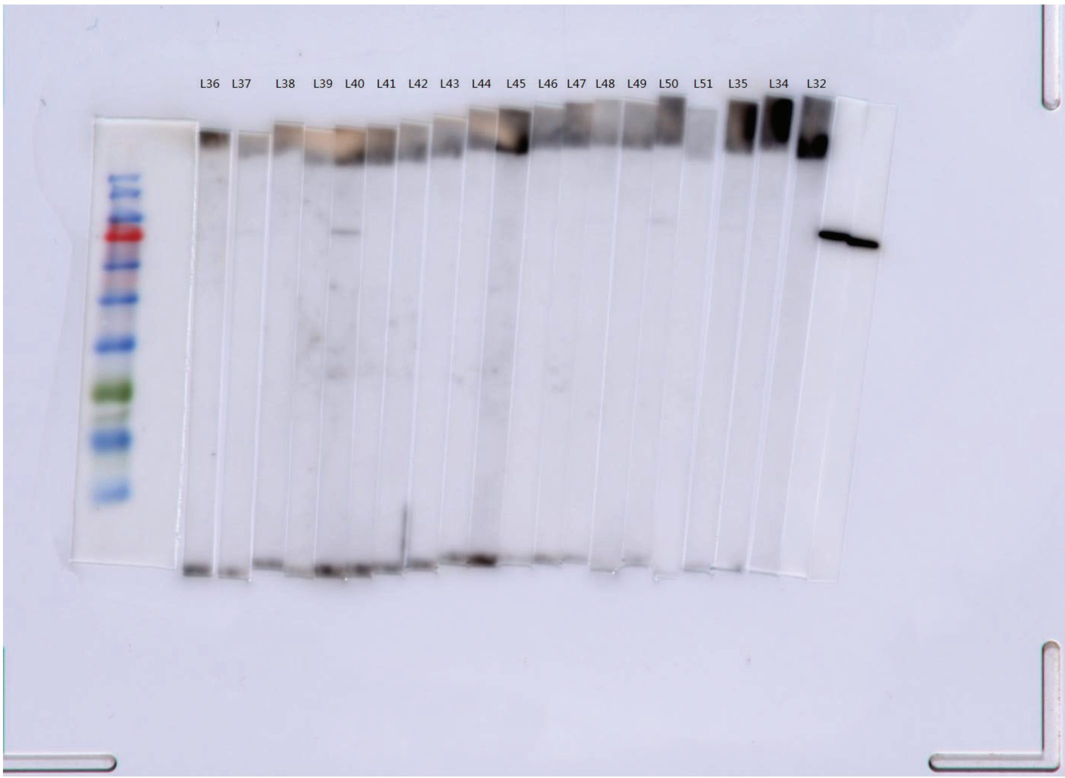
L36 L37 L38 L39 L40 L41 L42 L43 L44 L45 L46 L47 L48 L49 L50 L51 L35 L34 L32

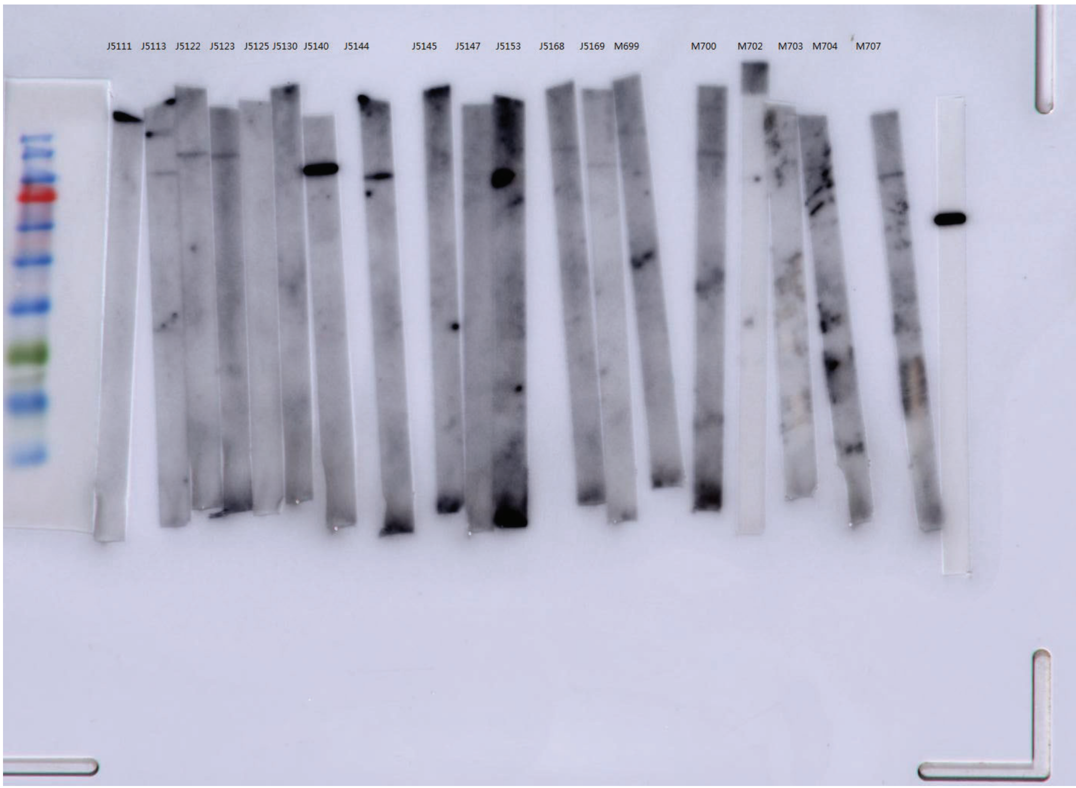
J5111 J5113 J5122 J5123 J5125 J5130 J5140 J5144 J5145 J5147 J5153 J5168 J5169 M699 M700 M702 M703 M704 M707

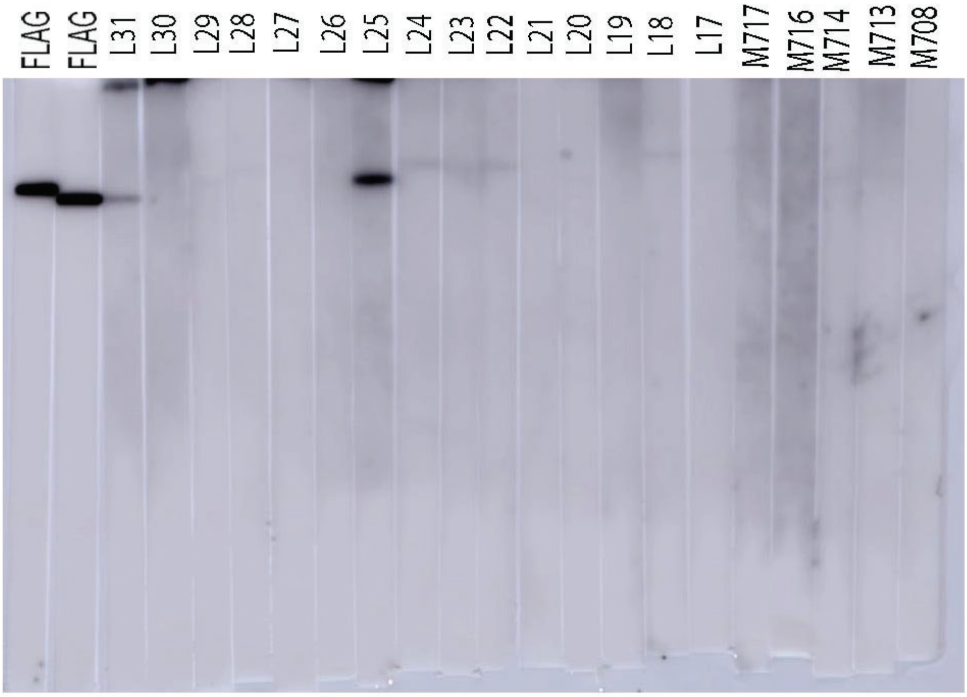
FLAG
FLAG
L31
L30
L29
L28
L27
L26
L25
L24
L23
L22
L21
L20
L19
L18
L17
M717
M716
M714
M713
M708

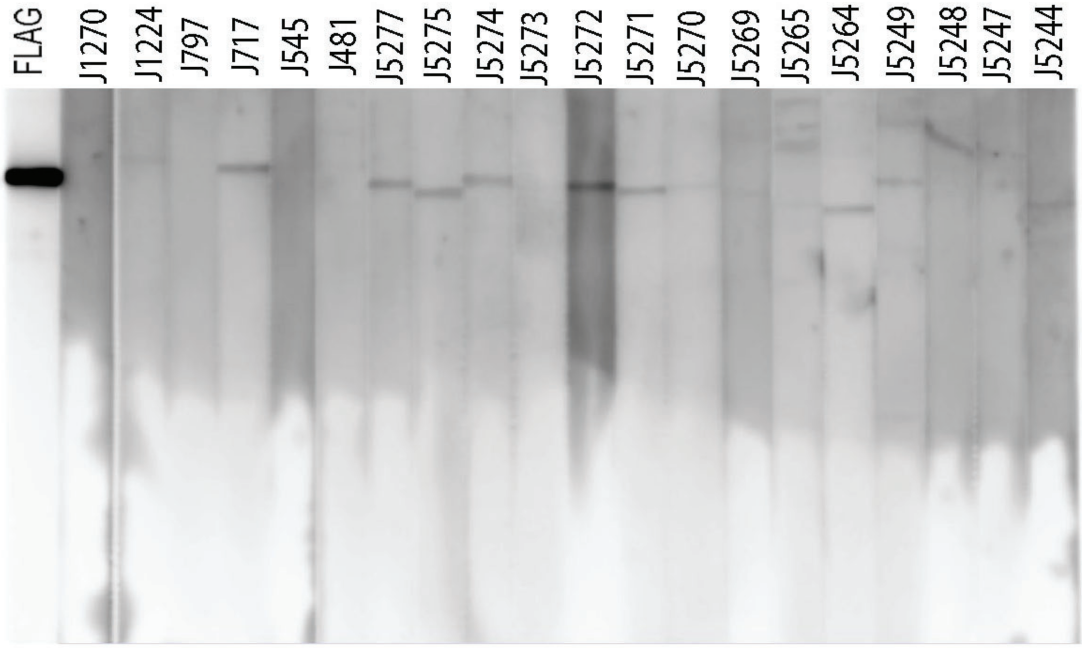
FLAG
J1270
J1224
J797
J717
J545
J481
J5277
J5275
J5274
J5273
J5272
J5271
J5270
J5269
J5265
J5264
J5249
J5248
J5247
J5244

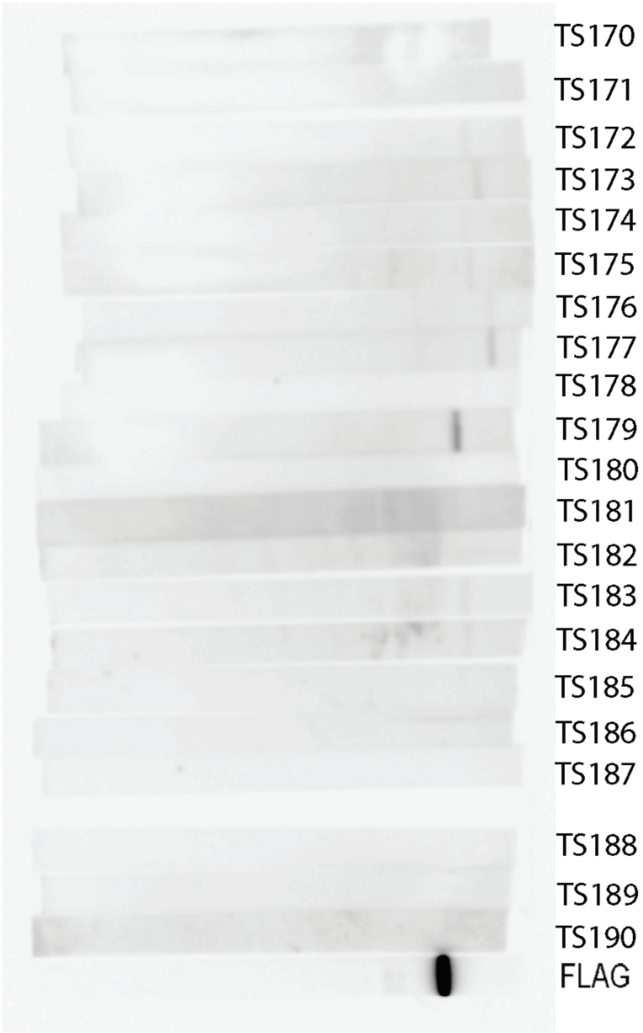
TS170
TS171
TS172
TS173
TS174
TS175
TS176
TS177
TS178
TS179
TS180
TS181
TS182
TS183
TS184
TS185
TS186
TS187
TS188
TS189
TS190
FLAG

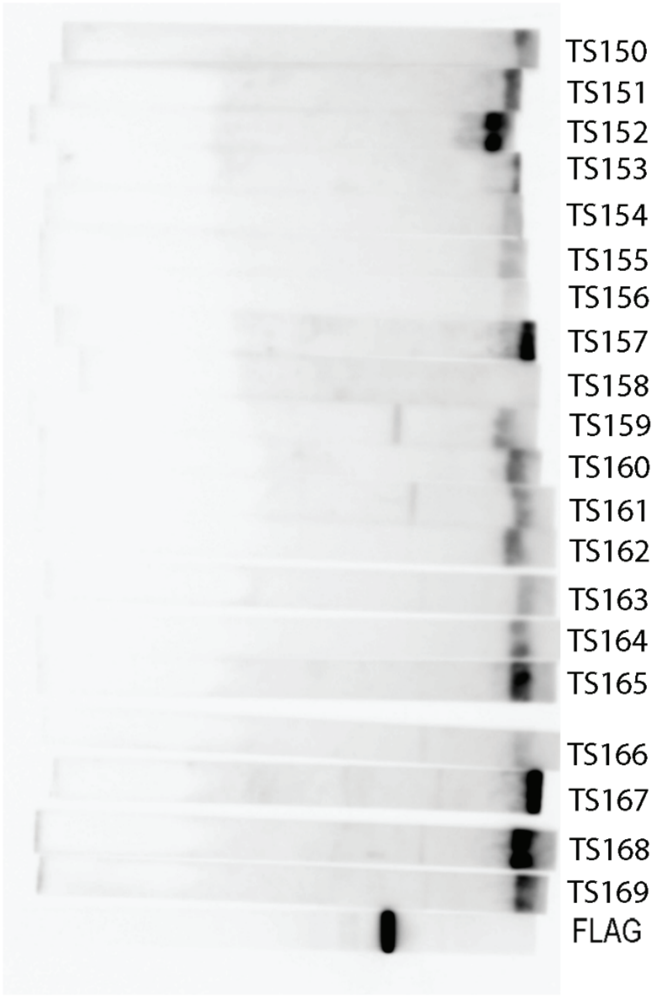
TS150
TS151
TS152
TS153
TS154
TS155
TS156
TS157
TS158
TS159
TS160
TS161
TS162
TS163
TS164
TS165
TS166
TS167
TS168
TS169
FLAG

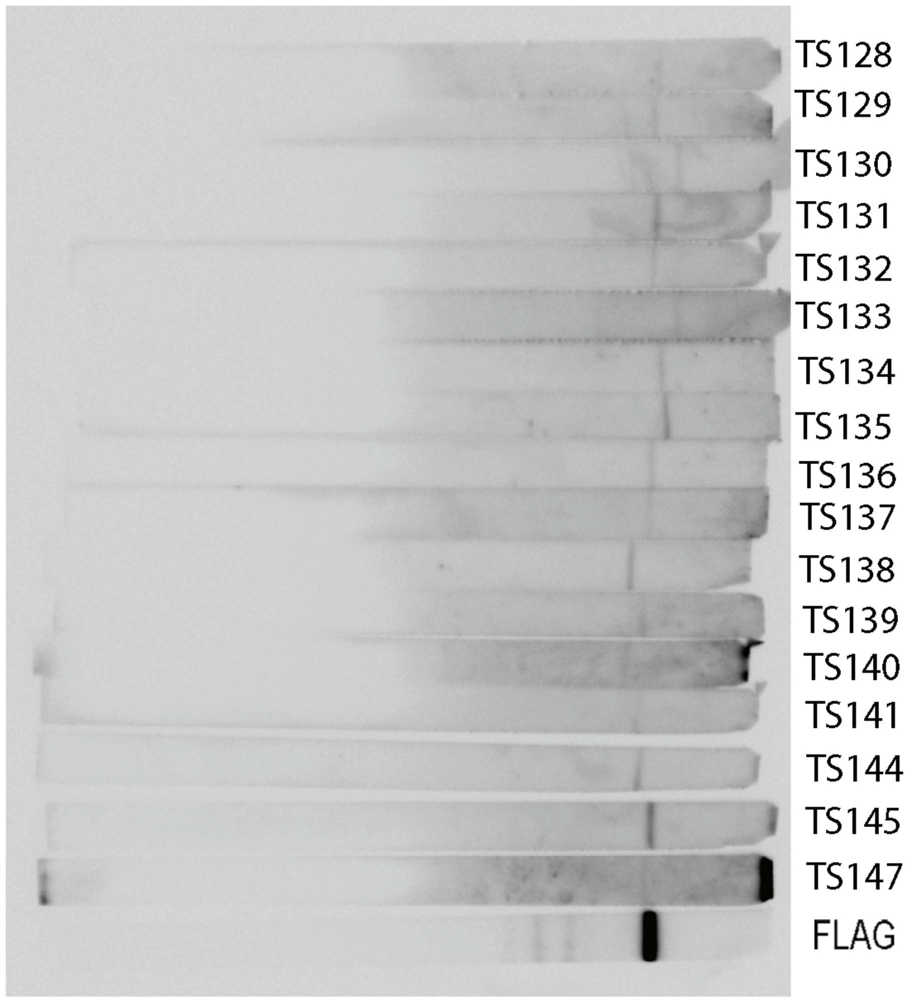
TS128
TS129
TS130
TS131
TS132
TS133
TS134
TS135
TS136
TS137
TS138
TS139
TS140
TS141
TS144
TS145
TS147
FLAG

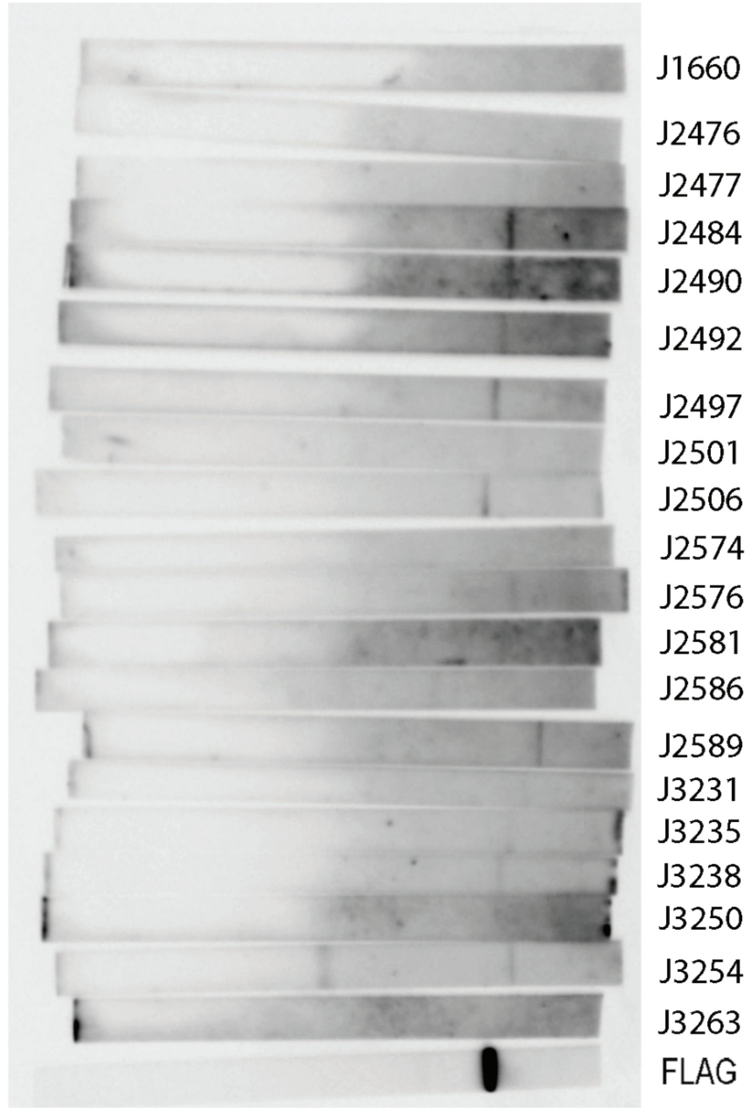
J1660
J2476
J2477
J2484
J2490
J2492
J2497
J2501
J2506
J2574
J2576
J2581
J2586
J2589
J3231
J3235
J3238
J3250
J3254
J3263
FLAG

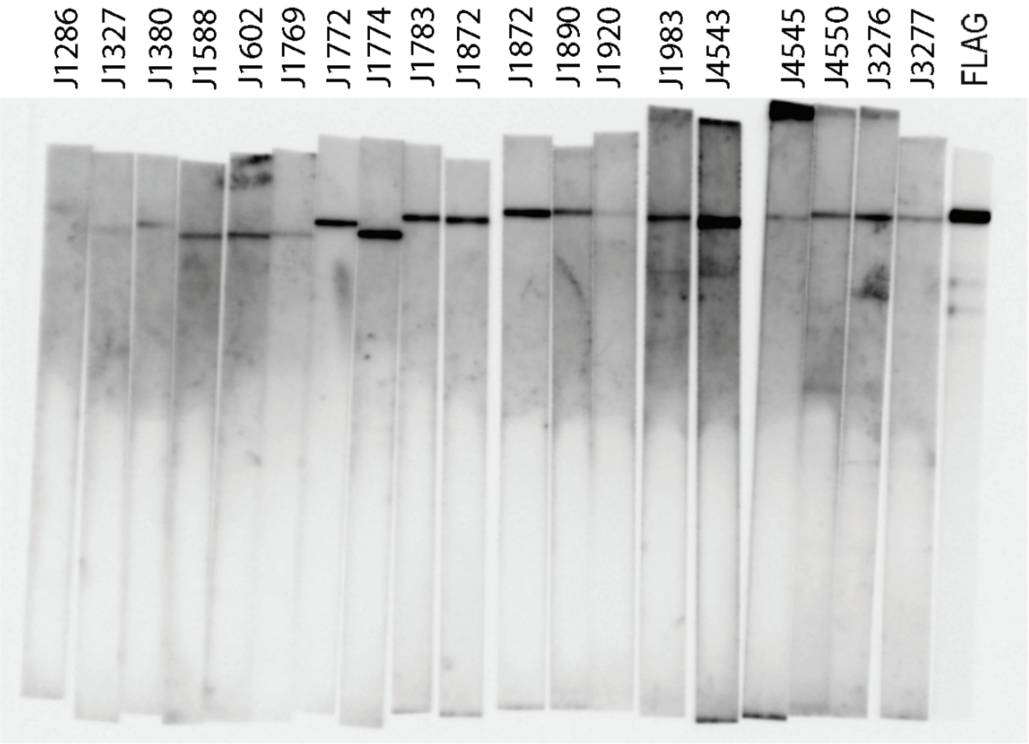
J1286
J1327
J1380
J1588
J1602
J1769
J1772
J1774
J1783
J1872
J1872
J1890
J1920
J1983
J4543
J4545
J4550
J3276
J3277
FLAG

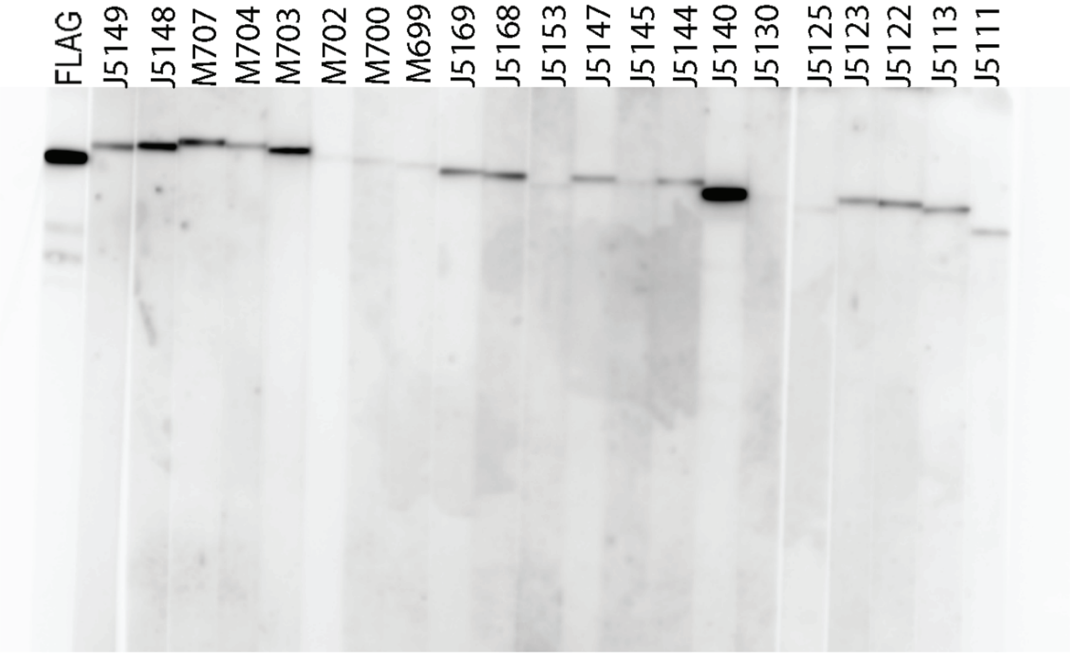
FLAG
J5149
J5148
M707
M704
M703
M702
M700
M699
J5169
J5168
J5153
J5147
J5145
J5144
J5140
J5130
J5125
J5123
J5122
J5113
J5111

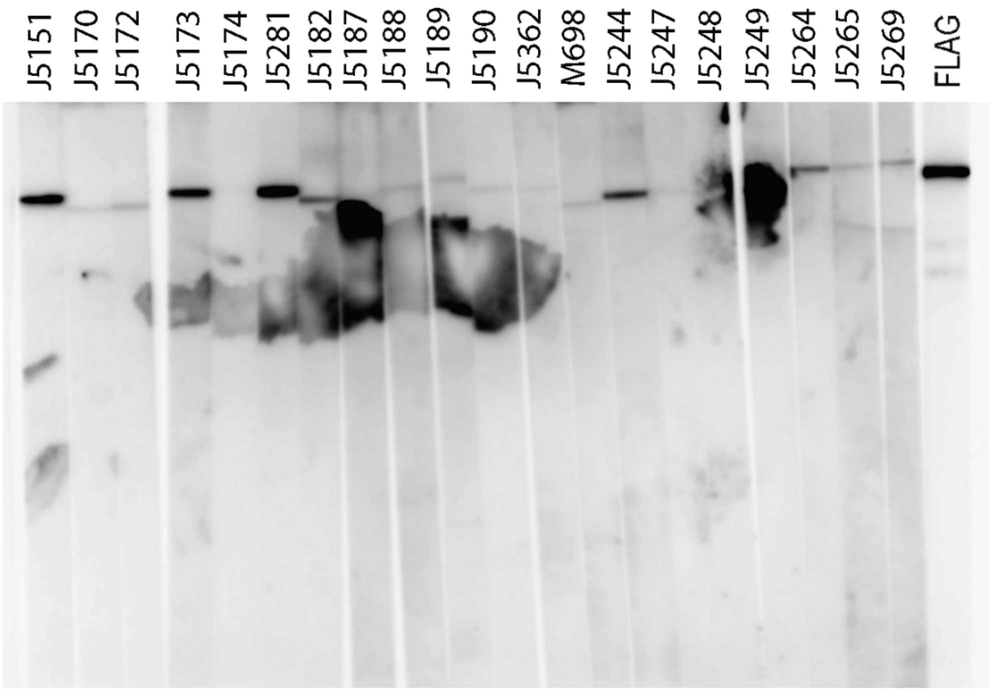
J5151
J5170
J5172
J5173
J5174
J5281
J5182
J5187
J5188
J5189
J5190
J5362
M698
J5244
J5247
J5248
J5249
J5264
J5265
J5269
FLAG

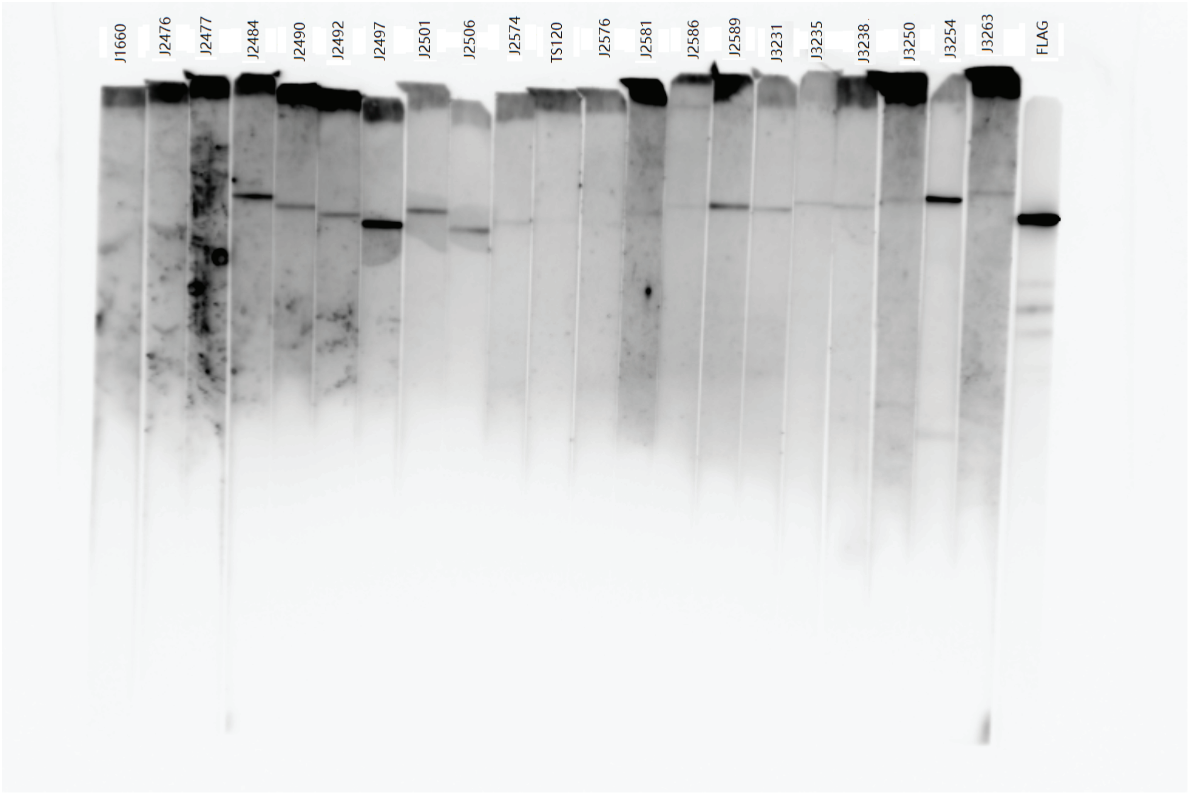
J1660
J2476
J2477
J2484
J2490
J2492
J2497
J2501
J2506
J2574
TS120
J2576
J2581
J2586
J2589
J3231
J3235
J3238
J3250
J3254
J3263
FLAG

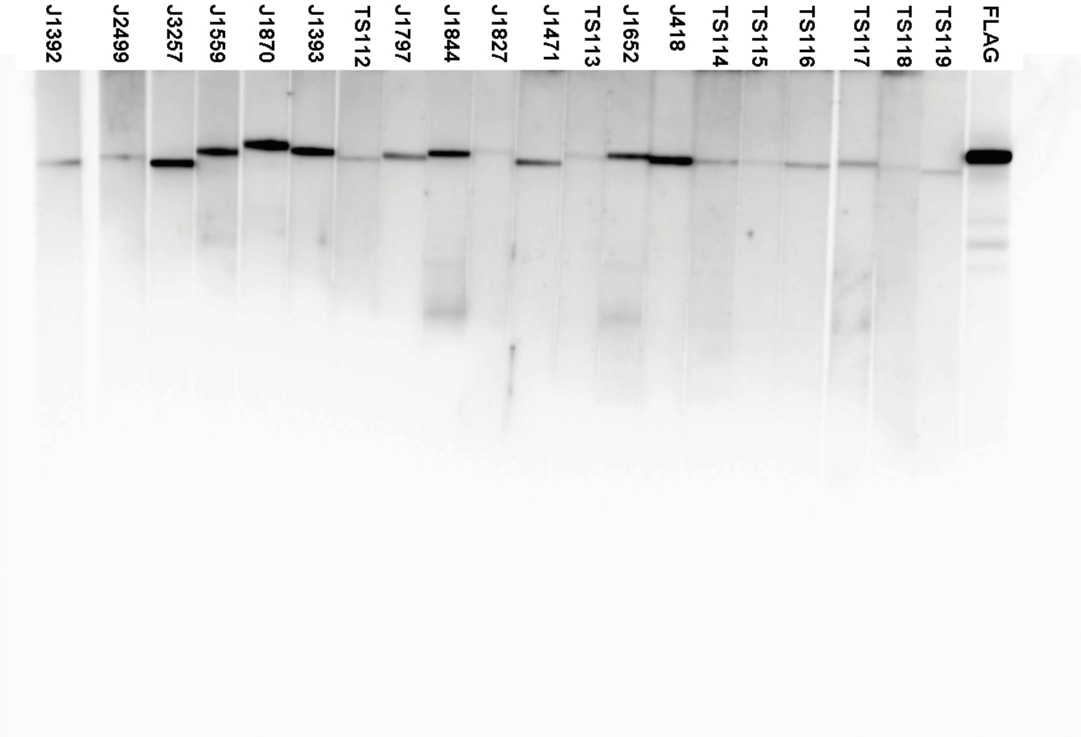
J1392
J2499
J3257
J1559
J1870
J1393
TS112
J1797
J1844
J1827
J1471
TS113
J1652
J418
TS114
TS115
TS116
TS117
TS118
TS119
FLAG

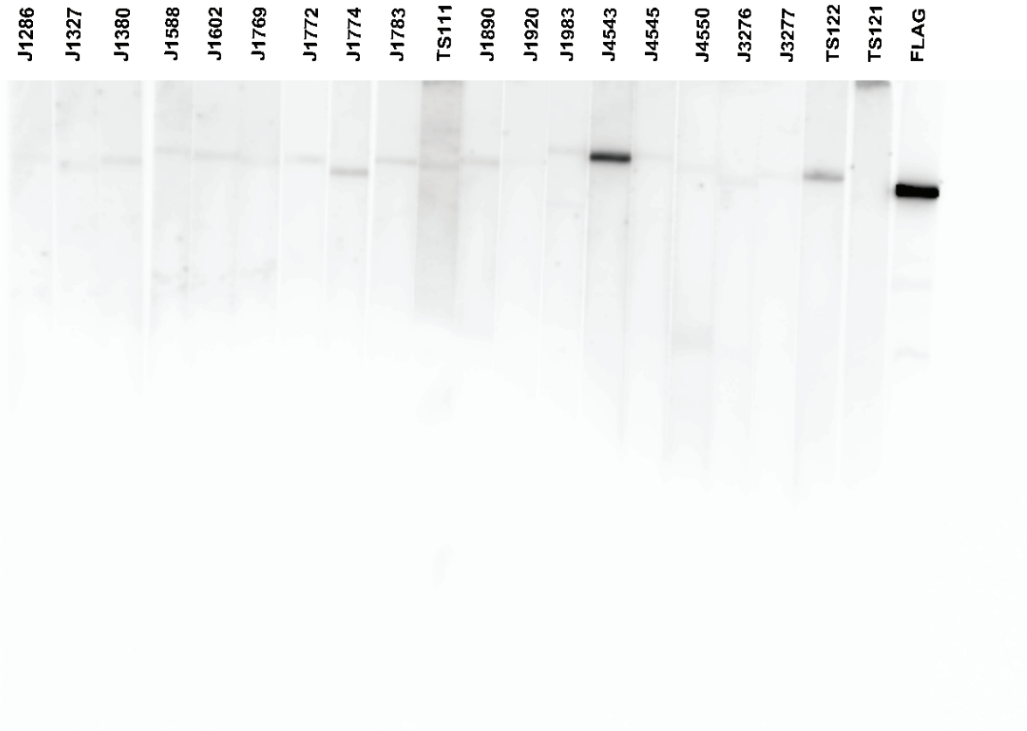
J1286
J1327
J1380
J1588
J1602
J1769
J1772
J1774
J1783
TS111
J1890
J1920
J1983
J4543
J4545
J4550
J3276
J3277
TS122
TS121
FLAG

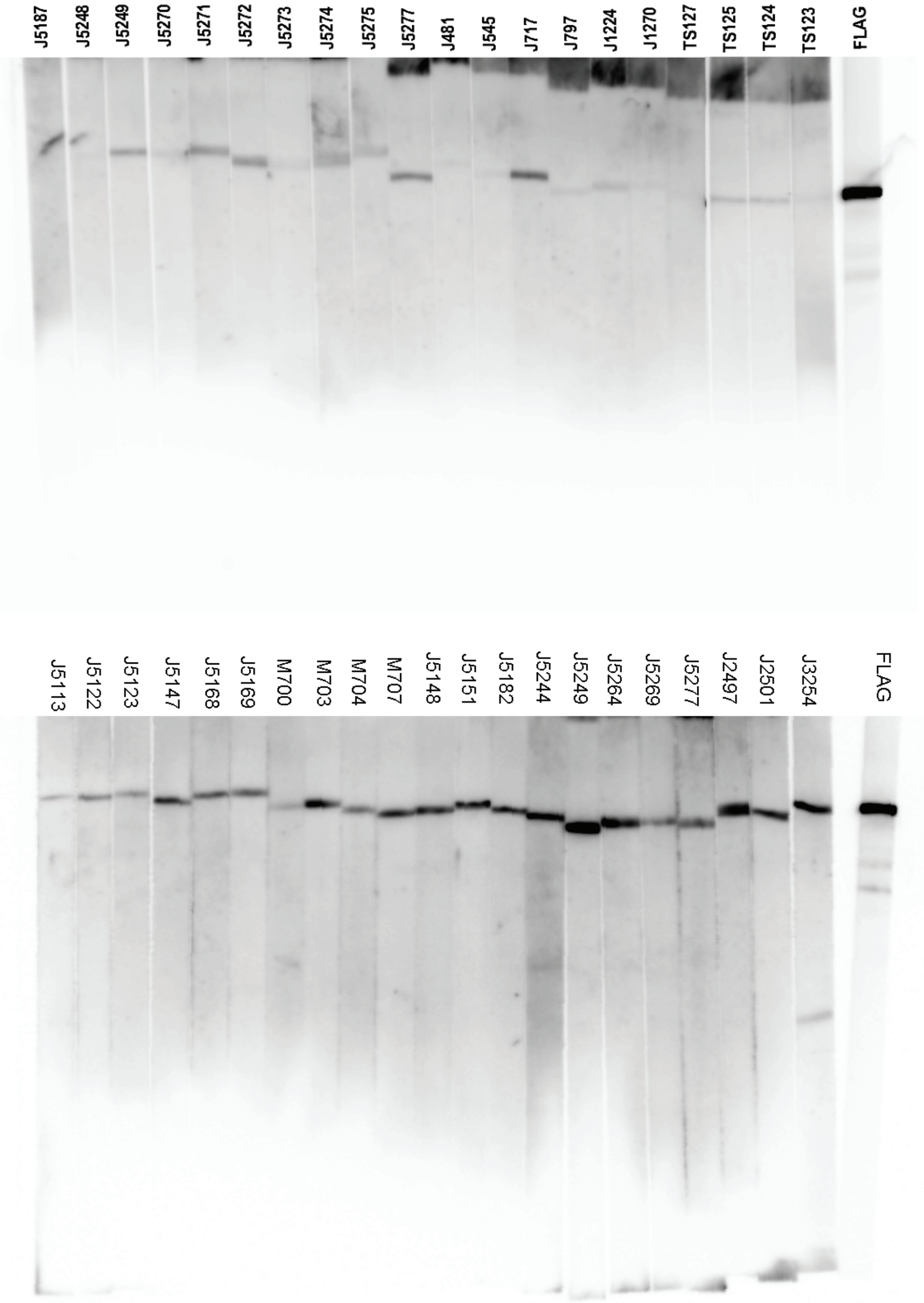
J5187
J5248
J5249
J5270
J5271
J5272
J5273
J5274
J5275
J5277
J481
J545
J717
J797
J1224
J1270
TS127
TS125
TS124
TS123
FLAG
J5113
J5122
J5123
J5147
J5168
J5169
M700
M703
M704
M707
J5148
J5151
J5182
J5244
J5249
J5264
J5269
J5277
J2497
J2501
J3254
FLAG

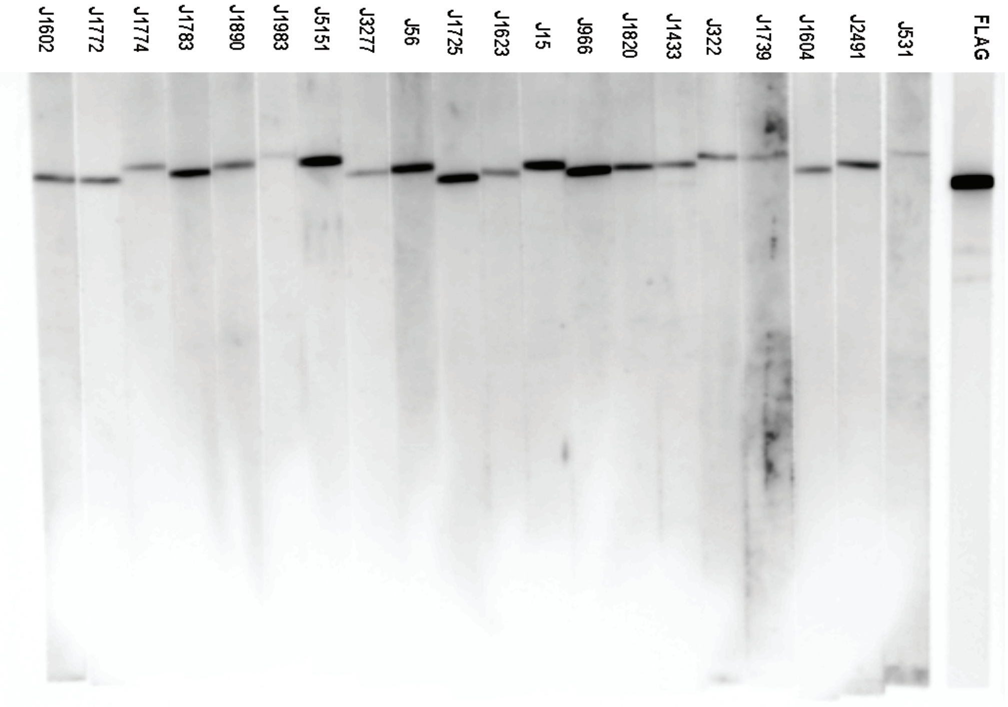
J1602
J1772
J1774
J1783
J1890
J1983
J5151
J3277
J56
J1725
J1623
J15
J966
J1820
J1433
J322
J1739
J1604
J2491
J531
FLAG

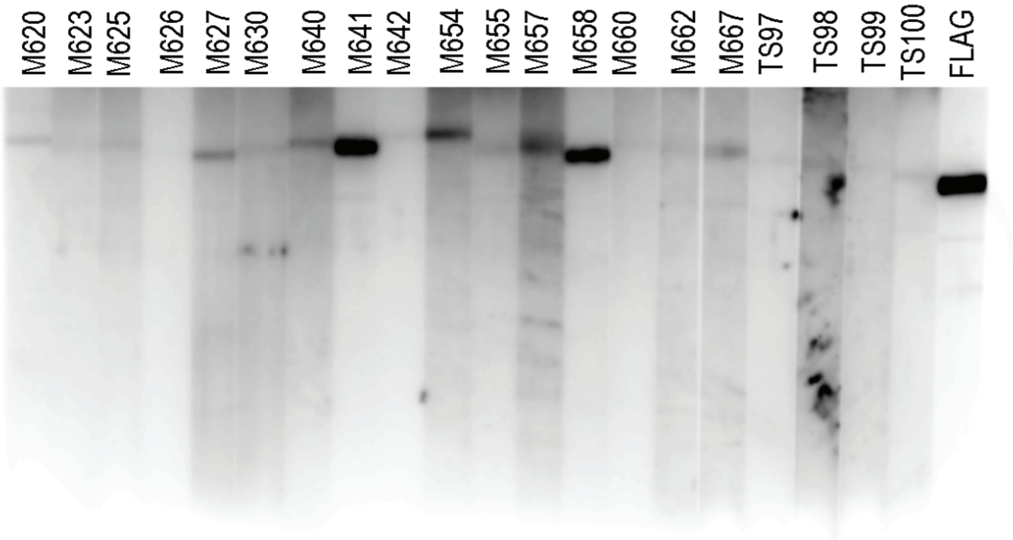
M620
M623
M625
M626
M627
M630
M640
M641
M642
M654
M655
M657
M658
M660
M662
M667
TS97
TS98
TS99
TS100
FLAG

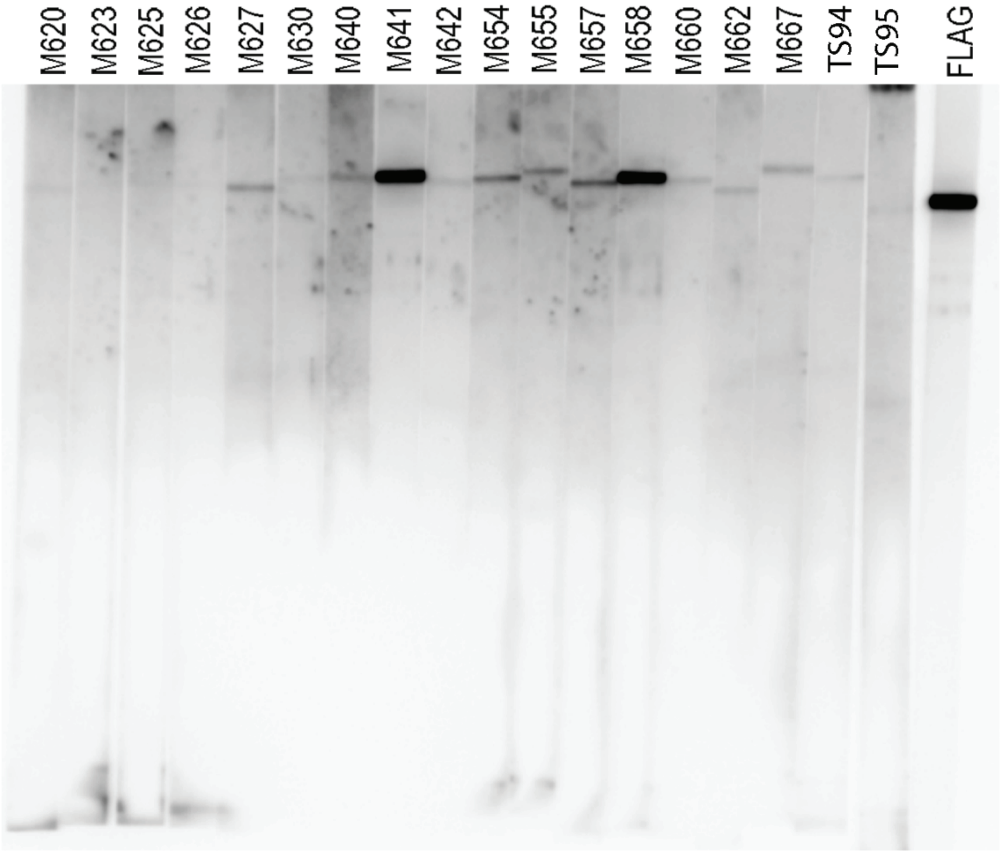
M620
M623
M625
M626
M627
M630
M640
M641
M642
M654
M655
M657
M658
M660
M662
M667
TS94
TS95
FLAG

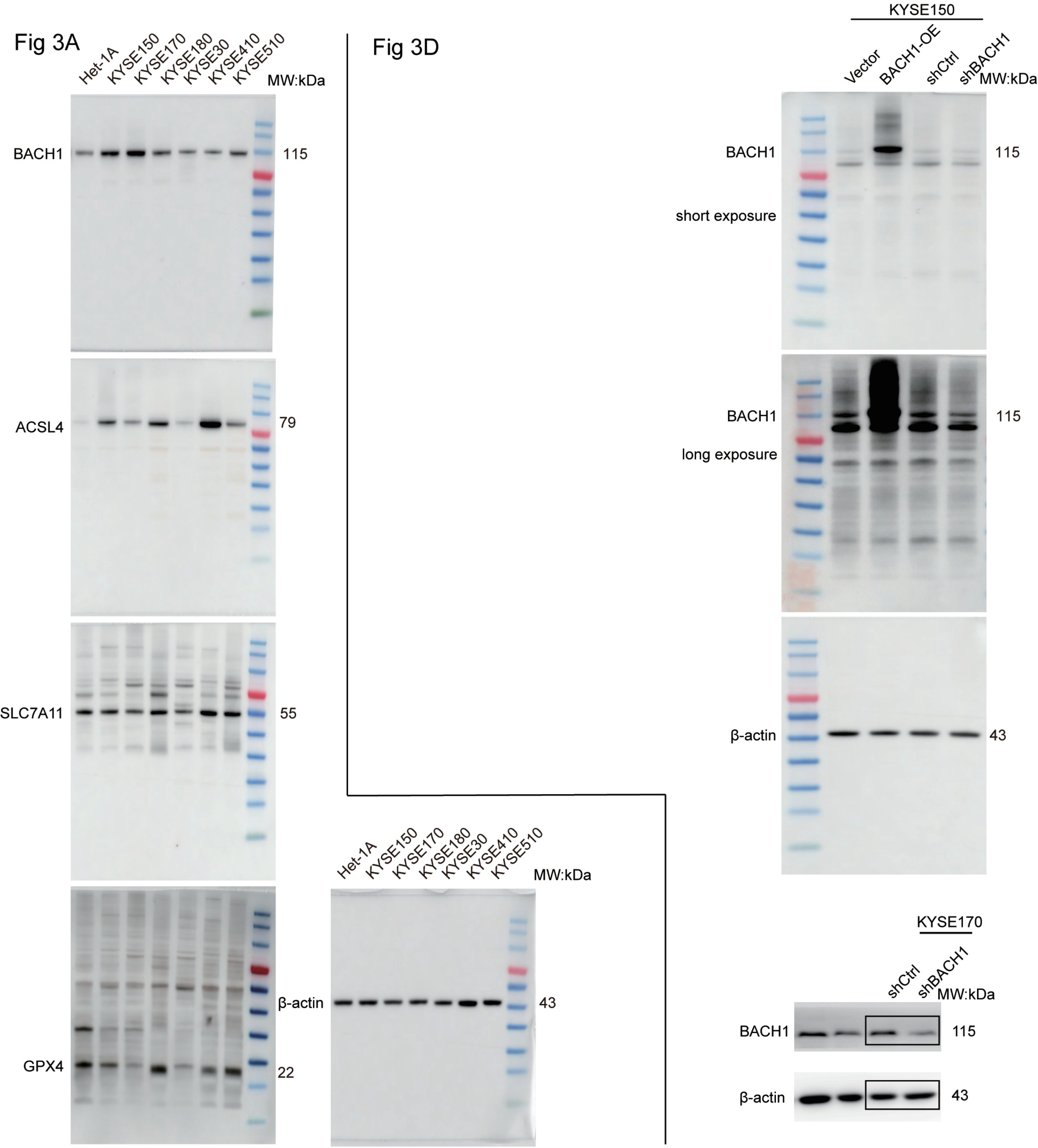
Fig 3A
Het-1A
KYSE150
KYSE170
KYSE180
KYSE30
KYSE410
KYSE510
MW:kDa
BACH1
115
ACSL4
79
SLC7A11
55
GPX4
22
β-actin
43
Fig 3D
KYSE150
Vector
BACH1-OE
shCtrl
shBACH1
MW:kDa
BACH1
short exposure
115
BACH1
long exposure
115
β-actin
43
KYSE170
shCtrl
shBACH1
MW:kDa
BACH1
115
β-actin
43

Fig 6D

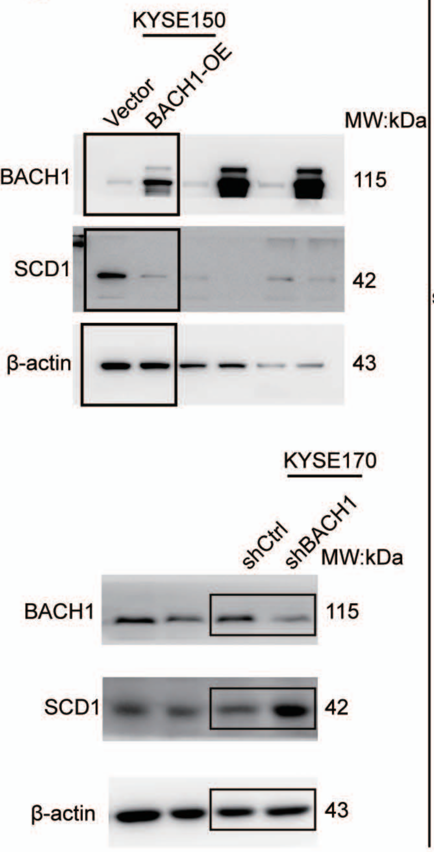

Fig 6D

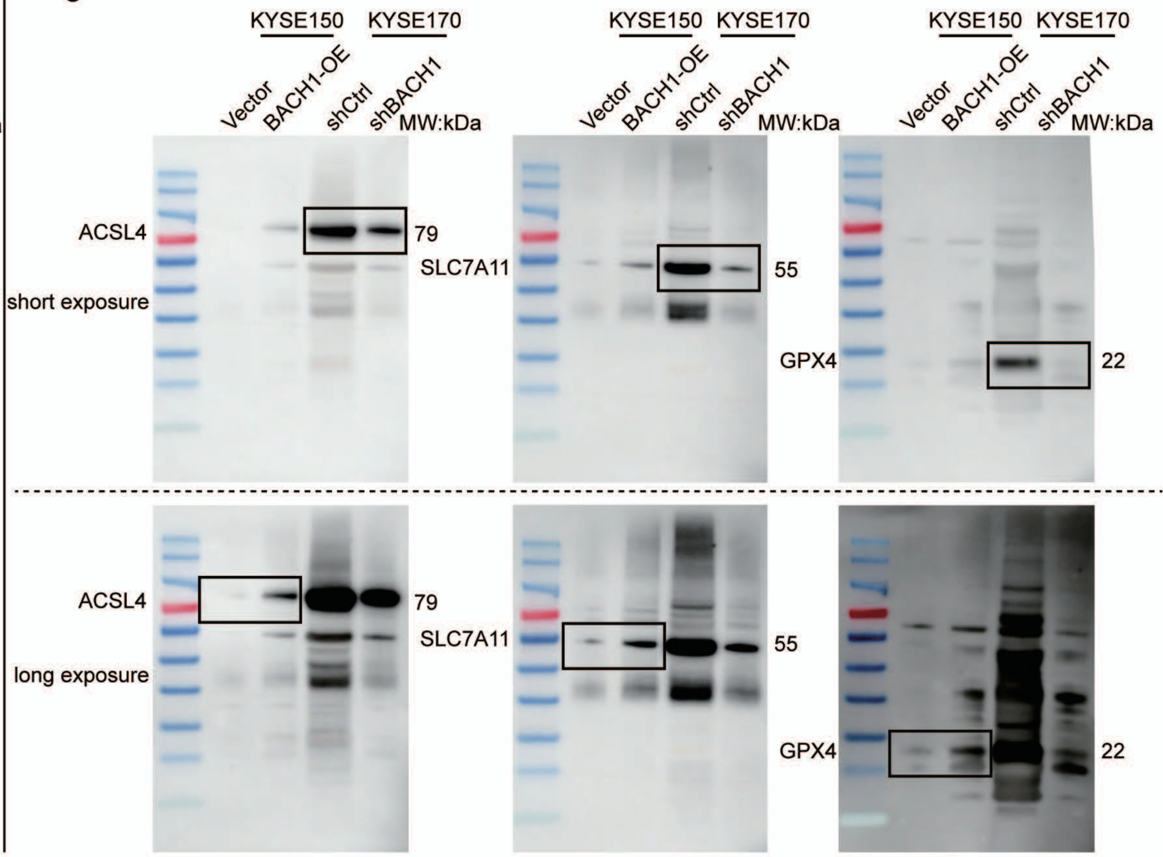

Fig 6G

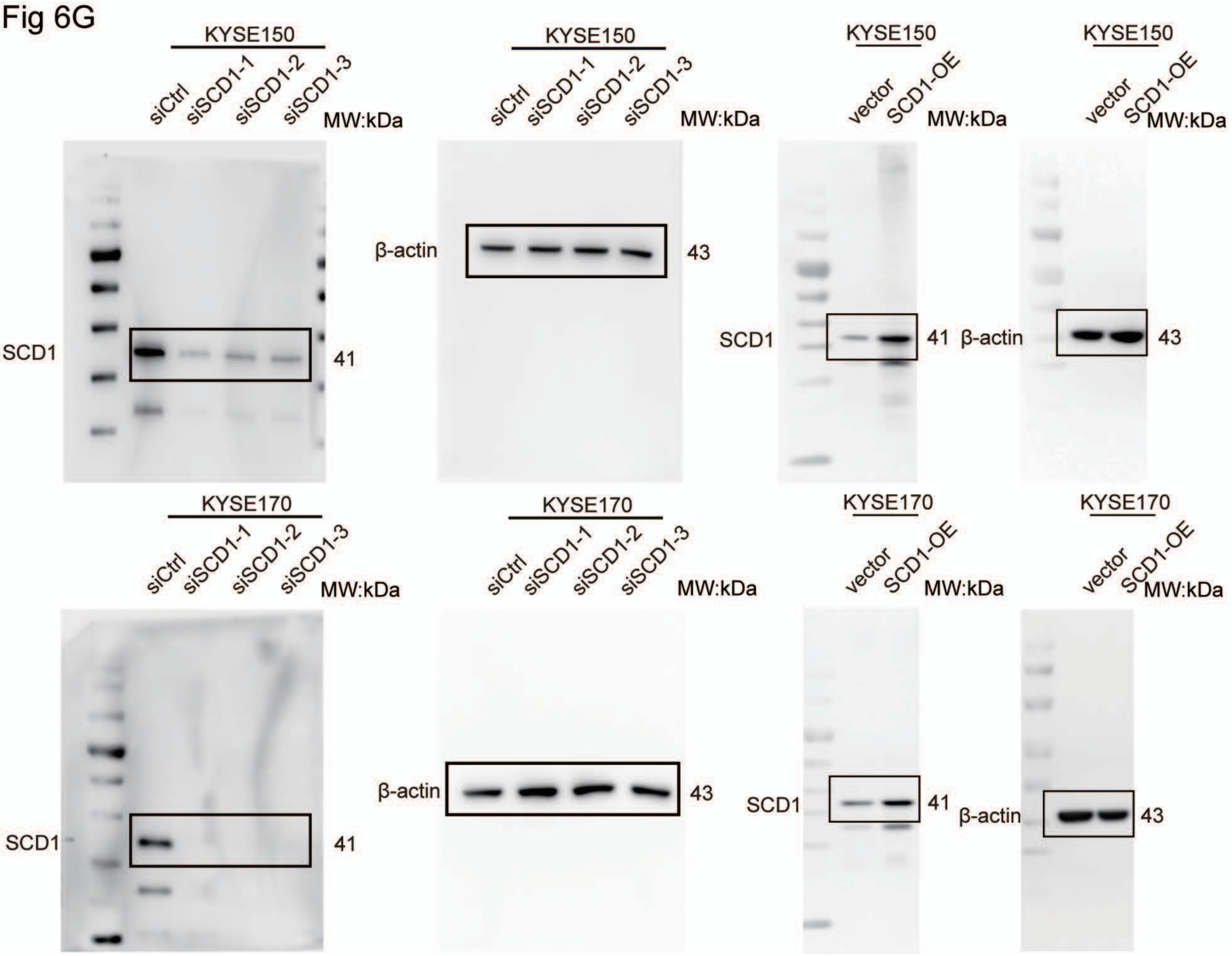

Supplement: Supplementary file 3 — Original Data File [file 41419_2023_5571_MOESM3_ESM.pdf]
